# Tunable Self-Assembly of Decanuclear Ni(II) Carbonato Clusters with a Hydroxyquinolinato Shell: Robust Porous Networks with Reversible Solvent-/Temperature-Driven Phase Transitions and Selective Gas Separation

**DOI:** 10.1021/jacs.5c04096

**Published:** 2025-05-27

**Authors:** Katarzyna Sołtys-Brzostek, Kamil Sokołowski, Iwona Justyniak, Aurelia Li, David Fairen-Jimenez, Alicja Supeł, Michał Terlecki, Janusz Lewiński

**Affiliations:** † Institute of Physical Chemistry, Polish Academy of Sciences, Kasprzaka 44/52, Warsaw 01-224, Poland; ‡ The Adsorption & Advanced Materials Laboratory (A^2^ML), Department of Chemical Engineering & Biotechnology, 2152 University of Cambridge, Philippa Fawcett Drive, Cambridge CB3 0AS, U.K.; § Faculty of Chemistry, 49566 Warsaw University of Technology, Noakowskiego 3, Warsaw 00-664, Poland

## Abstract

The utilization of molecular metal clusters as building units of noncovalent porous materials (NPMs) is a promising strategy, combining the versatile functionality of organic and inorganic subunits with the softness and flexibility of molecular solids controlled by noncovalent interactions. However, the development of robust porous functional frameworks based on self-assembly driven by noncovalent forces is still highly challenging. Herein, we report the synthesis and characterization of a discrete decanuclear Ni­(II) hydroxyquinolinato-carbonato cluster, [Ni_10_(μ_6_-CO_3_)_4_(L)_12_], which, depending on the crystallization conditions, self-assembles into either of two microporous frameworks: diamondoid **WUT-1­(Ni)** and pyrite **WUT-2­(Ni)**. The transitions between both polymorphs can also be selectively triggered by temperature or exposure to vapors of a particular organic solvent, which is accompanied by the easy recovery of crystallinity by the materials from the noncrystalline phase. Moreover, both materials show excellent robustness toward various chemical environments, including air/moisture and water stability, and demonstrate interesting gas adsorption properties. Remarkably, **WUT-1­(Ni)** exhibits significant enhancement in gas uptake compared to the previously reported isostructural Zn­(II) analogue, **WUT-1­(Zn)**, representing one of the highest H_2_ uptakes among NPMs. In turn, tighter voids of the ultramicroporous **WUT-2­(Ni)** framework facilitate selective interactions with gas molecules, resulting in outstanding selectivity in the adsorption of CO_2_ over CH_4_ and N_2_. The presented studies demonstrate the profound role of the character of metal centers on the self-assembly of isostructural nanoclusters as well as properties of the resulting microporous frameworks.

## Graphical abstract

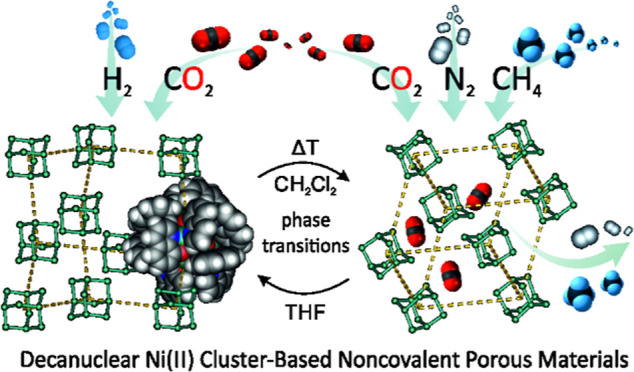

## Introduction

Substantial progress has been achieved in the rational design and modular construction of ordered porous solids in the past decades.^1^ Voids in porous materials provide a unique environment for encapsulating species, and the proper adjustment and functionalization of pores ensure control over host–guest interactions. Thus, the fine-tuning of pores may not only facilitate the adsorption properties of the material, increasing guest uptake,^2,3^ but also pave the way for novel guest-specific applications such as enantioseparation processes,^3,4^ stoichiometric and catalytic transformations,^5,6^ and the stabilization of reactive species and unusual reaction intermediates.^7,8^ For decades, the interest has mainly focused on porous materials based on extensive networks of covalent or coordination bonds such as naturally occurring zeolites,^9^ hybrid metal–organic frameworks (MOFs),^10–12^ and, more recently, covalent organic frameworks (COFs).^13,14^ In particular, the development of MOFs at the turn of the 20th–21st centuries gave substantial momentum to the rational design of porous, supramolecular frameworks,^10^ introducing unprecedented control over the supramolecular topology and structure of the void spaces. Recently, increasing attention has been paid to structurally adaptable molecular systems^15–17^ and robust noncovalent microporous materials (NPMs)—also known as molecular porous materials (MPMs) or porous molecular crystals (PMCs)—based on the self-assembly of discrete molecules driven by noncovalent interactions.^18–20^ Among this family, the majority of materials are based on organic molecules, which involve hydrogen-bonded organic frameworks (HOFs)^21–25^ or soft organic frameworks (SOFs).^19,26,27^ In recent years, less common examples of porous molecular crystals based on noncovalent interactions-driven self-assembly of molecular metal complexes or clusters have also been developed.^28–39^ The porous properties of these materials result either form internal cavities within the structure of molecular building units, like in metal–organic cages,^30,31,36–39^ from specific nonclosed packing of molecules in the crystal lattice,^28,29,32–35^ or from adaptive guest accommodation abilities.^15–17^ Hence, these hybrid organic–inorganic materials combine the versatile functionality of MOFs provided by the presence of both metal centers and organic subunits with the softness and flexibility of noncovalent frameworks. The framework flexibility facilitates the development of stimuli-responsive materials, like nanovalves and crystalline sponges.^40–42^ In turn, the tunable host–guest interactions in NPMs enable their application in guest-specific processes like enantioseparation of small molecules,^15,38^ selective gas adsorption,^43–47^ sensing,^48,49^ or catalysis.^50,51^ Notably, these materials have been successfully used in adsorption-based separation of the principal component of flue gas (which typically consists of N_2_, CO_2_, and SO_2_), often with better efficiency than the established porous materials,^25,47,49–52^ providing new opportunities for developing unconventional and superior porous materials for different types of gas separation and purification. Strikingly, the solution processability of NPMs allows for their easy characterization and purification or facile regeneration and reuse by simple recrystallization.

The design of foreseeable porous functional frameworks based on the self-assembly driven by noncovalent forces is highly challenging due to the cooperative mode of action of multiple weak interactions, which do not always follow the expected roadmap.^55^ Moreover, molecular building units exhibit a tendency for dense packing in the solid state, usually forming structures with minimal void volume. Thus, ensuring the permanent porosity of NPMs is demanding as their frameworks are often very fragile, which results in the collapse of the porous structure during the removal of guest/solvent molecules or upon heating. Most applications for porous materials also require chemical stability in air and in the presence of moisture or high temperature.^56^ Although the synthetic variability of NPMs enables a large design space for novel materials with improved stability, it is still challenging to obtain noncovalent porous frameworks that are robust enough for their potential applications.

During our investigations on molecular metal complexes as building blocks for various structurally adaptable molecular systems^17,57,58^ and microporous materials,^15,59,60^ for example, we have developed a series of chiral aluminum–cinchonine complexes with excellent capability for noncovalent interactions-driven self-assembly into novel NPMs,^15,60^ which achieved outstanding results in the enantioselective inclusion of 2-MeTHF and selective adsorption of H_2_ and CO_2_ over N_2_. Succeeding investigations led us to the development of a novel permanently porous NPM, denoted **WUT-1** (hereafter **WUT-1­(Zn)**; WUT–Warsaw University of Technology),^61,62^ which was constructed from discrete molecular building blocks of highly luminescent decanuclear zinc carbonato-hydroxyquinolinato nanoclusters [Zn_10_(μ_6_-CO_3_)_4_(L)_12_] (where L = 8-hydroxyquinolinate ligand), derived from the fixation of CO_2_ by a predesigned alkylzinc hydroxide precursor. The discrete molecular [Zn_10_(μ_6_-CO_3_)_4_(L)_12_] clusters self-assembled into a 3D, permanently microporous structure within a diamondoid crystal lattice with a Brunauer–Emmett–Teller (BET) area of 1225 m^2^ g^–1^, one of the highest of all known NPMs. The experimental gas adsorption isotherms confirmed significant adsorption of selected gases N_2_, H_2_, CO_2_, and CH_4_ at ambient and higher pressures. Additionally, **WUT-1­(Zn)** exhibited luminescent properties with a very high quantum yield of 65%. Although this material was stable upon guest removal, it showed significant moisture sensitivity in the solid state as well as in solution. In the meantime, analogous hydroxyquinolinato decanuclear Co­(II) and Mn­(II) carbonate clusters, [Co_10_(μ_6_-CO_3_)_4_(L)_12_] and [Mn_10_(μ_6_-CO_3_)_4_(L)_12_], were serendipitously obtained.^63,64^ However, contrary to the **WUT-1­(Zn)** material, these complexes adopt the pyrite-type supramolecular structure and were described by the authors as being close-packed and nonporous.

As part of our ongoing research in the systematic modification of the metal center of carbonate-based NPMs, herein we describe the synthesis and characterization of a discrete decanuclear Ni­(II) hydroxyquinolinato cluster, [Ni_10_(μ_6_-CO_3_)_4_(L)_12_] (**1**). Remarkably, this nanocluster as a building block exhibits distinct assembly modes compared to diamondoid microporous **WUT-1­(Ni)** or densely packed pyrite **WUT-2­(Ni)** frameworks depending on the crystallization conditions, and reversible phase transitions between both polymorphs can be induced by temperature or solvent treatment. Moreover, **WUT-1­(Ni)** and **WUT-2­(Ni)** molecular solids display vapor-solvent-triggered tunability combined with crystallinity recovery ability. Both frameworks also reveal excellent thermal and chemical stability, both under aerobic and aqueous conditions, and interesting microporous properties, facilitating either high gas uptake in extended pores of **WUT-1­(Ni)** or high gas adsorption selectivity in tighter voids of ultramicroporous **WUT-2­(Ni)**. Furthermore, comparison of the developed Ni­(II)-based materials with the previously reported isostructural assemblies of [M_10_(μ_6_-CO_3_)_4_(L)_12_]-type clusters (M = Zn­(II), Mn­(II), Co­(II)) demonstrates the profound role of the character of metal centers on assembly modes and properties of the resulting microporous frameworks.

## Results and Discussion

### Synthesis, Structure, and Phase Stability of **WUT-1­(Ni)** and **WUT-2­(Ni)**

#### Synthesis

Initially, in control experiments, 10 equiv Ni­(OAc)_2_·4H_2_O was reacted with 12 equiv 8-hydroxyquinoline (L–H) in dimethylformamide (DMF) with different amounts of NaOH_aq_ in the presence of carbon dioxide (Figure S6). We found that the chemical fixation of CO_2_ molecules is sensitive to alkaline conditions, shifting the reaction equilibrium into carbonate species, and the amount and concentration of the used base influence the phase composition and purity of the resulting crude solids. The reaction with a small amount of NaOHaq (10:12:1.5 molar ratio of Ni­(OAc)_2_·4H_2_O/L–H/NaOH) afforded a nickel hydroxyquinolinato-carbonato cluster [Ni_10_(μ_6_-CO_3_)_4_(L)_12_] (**1**) in high yield (Figure 1). Notably, these reaction conditions repeatedly and selectively led to a diamondoid microporous lattice **WUT-1­(Ni)** (in ca. 50% yield), which was confirmed by powder X-ray diffraction (PXRD) analysis (Figure S6). In turn, the presence of a higher amount of NaOH_aq_ in the analogous reaction (10:12:10 molar ratio of Ni­(OAc)_2_·4H_2_O/L-H/NaOH) also led to cluster **1** but in a mixture of two crystal phases: the diamondoid **WUT-1­(Ni)** and a pyrite **WUT-2­(Ni)**, as judged from the PXRD analysis (Figures S6 and S8). The character of the base also affects the reaction outcome. For example, the utilization of N­(CH_3_)_4_OH instead of NaOH as the source of OH^–^ selectively led to the diamondoid phase **WUT-1­(Ni)** in high yield (75%), even with a higher amount of the base, e.g., Ni­(OAc)_2_·4H_2_O/L–H/N­(CH_3_)_4_OH and a molar ratio of 10:12:12 (Figure S7). Interestingly, the pure **WUT-2­(Ni)** phase can be obtained by prolonged heating of **WUT-1­(Ni)** at 270 °C (vide infra). Selected dark green octahedral crystals of **WUT-1­(Ni)** (Figure S24a) and green triangular crystals of **WUT-2­(Ni)** (Figure S24c) were analyzed by single-crystal X-ray diffraction (SC-XRD), and both bulk **WUT-1­(Ni)** and **WUT-2­(Ni)** materials were characterized by PXRD, FTIR spectroscopy, and sorption analysis (for details, see Supporting Information).

**Figure 1:**
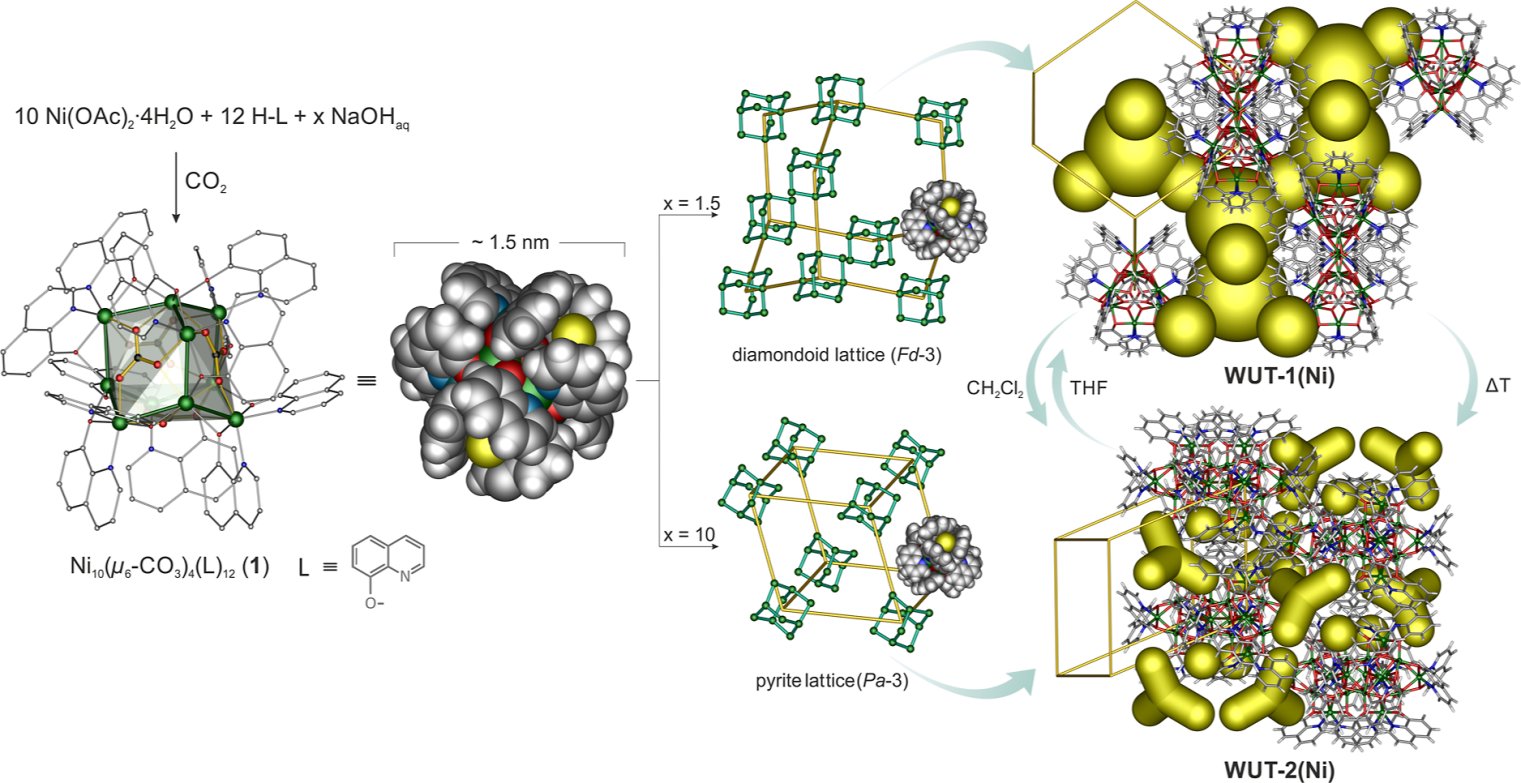
Synthesis and molecular structure of **1** and a schematic illustration of the dia ↔ pyr interconversion of the polymorphic forms of **1** induced by solvent or temperature.

#### Structure Characterization

The nickel cluster **1** is isostructural with the previously reported zinc hydroxyquinolinato-carbonato [Zn_10_(μ_6_-CO_3_)_4_(L)_12_].^61^ Its decanuclear [Ni_10_(μ_6_-CO_3_)_4_]^12+^ core resembles the structural motif of a diamondoid lattice composed of four fused hexanuclear {Ni_6_} macrocycles with the boat conformation, each containing a centrally coordinated μ_6_–μ_2_/μ_2_/μ_2_ carbonate anion. The respective intramolecular Ni–Ni distance is 3.120 Å, and the Ni–Ni–Ni angles are 100.33° and 113.63°, indicating only small geometrical deviations from the ideal cubic diamond structure. The inorganic core is decorated by an organic shell of 12 monoanionic 8-hydroxyquinolinate ligands. Each carbonato nickel {Ni_6_(CO_3_)} macrocycle is coordinated by six μ_2_-κ^2^(*N*,*O*)/κ^1^(*O*) ligand-bridging Ni­(II) centers at the edges, three in equatorial and three in axial positions respective to the macrocycle. The aromatic rings of three axial quinolinate ligands form triangular micropockets (ca. 3.5 Å in diameter) above each {Ni_6_} macrocycle, introducing intrinsic porosity to the organic ligand shell of **1** (Figure 1). Interestingly, these spherical decanuclear Ni­(II)-carbonato nanoclusters self-assemble through CH_Ar_···π cooperative interactions (Figures S4 and S5) into two types of frameworks: a diamondoid **WUT-1­(Ni)** and a pyrite **WUT-2­(Ni)**, depending on the crystallization conditions. In the former supramolecular structure **WUT-1­(Ni)**, the nanoclusters are packed into the microporous diamondoid lattice with two types of interconnected voids of ∼6 Å and ∼10 Å in diameter, which result in an accessible void fraction of 0.44 of the unit cell volume (based on CrystalExplorer^65^ calculations). The voids in the **WUT-1­(Ni)** framework are filled with DMF molecules, which in the crystal structure are located in the intrinsic micropockets of the nanocluster shell. We note that the supramolecular structure of **WUT-1­(Ni)** is essentially the same as that previously reported for **WUT-1­(Zn)**, but the latter framework featured a slightly higher accessible void fraction of 0.46 of the unit cell volume.^61^ In turn, in the **WUT-2­(Ni)** framework, the nanoclusters of **1** form the pyrite lattice with a more densely packed framework, which is similar to the previously serendipitously isolated isostructural Co­(II) and Mn­(II) hydroxyquinolinato-carbonato clusters, [Co_10_(μ_6_-CO_3_)_4_(L)_12_] and [Mn_10_(μ_6_-CO_3_)_4_(L)_12_]. Specifically, the supramolecular structures of the Co­(II) and Mn­(II) were described as essentially nonporous.^63,64^ Remarkably, a careful examination of the crystal structure of **WUT-2­(Ni)** revealed the presence of isolated voids with a complex shape resembling two centrally fused three-leaf clovers (Figure S3), which are formed by the assembly of the internal micropockets of the six neighboring nanoclusters. These separated voids, constituting about 0.20 of the unit cell volume (based on CrystalExplorer^65^ calculations), are accessible for small gas molecules, which is attributed to the thermal vibrations of the discrete decanuclear clusters in the framework of **WUT-2­(Ni)**.

#### Chemical and Thermal Stability of **WUT-1­(Ni)** and **WUT-2­(Ni)**

In view of practical applications, we have investigated the effects of water, organic solvents, and temperature on the structures of **WUT-1­(Ni)** and **WUT-2­(Ni)**, and the study demonstrated excellent stability of both materials. Particularly, PXRD analysis shows that the microporous **WUT-1­(Ni)** and **WUT-2­(Ni)** frameworks remained intact under atmospheric conditions at room temperature for months (Figures S10 and S14). They also exhibit robustness toward water both under acidic and basic aqueous conditions. The immersion of **WUT-1­(Ni)** crystals in water or aqueous solutions of CH_3_COOH (0.01 M, 0.1 M, 1 M) and NaOH (0.01 M, 0.1 M) for 48 h did not induce any structure disorder despite slight changes in the relative intensities of some peaks in the PXRD pattern (Figure S11a). A similar trend was noticed under moderately acidic aqueous conditions of 0.01 M HCl, while the higher concentrations degraded the sample. **WUT-2­(Ni)** exhibits even better stability of its crystal structure, as it remains stable in 1 and 2 M NaOH aqueous solutions for 48 h and in water for up to 2 weeks (Figure S15a). **WUT-1­(Ni)** is essentially insoluble in most common organic solvents, and its immersion in toluene, acetonitrile, THF, acetone, or EtOH (as a protic solvent) for 2 weeks did not result in significant changes in the PXRD patterns (Figure S11b), indicating that the crystal structure was unaffected. In turn, the treatment of **WUT-1­(Ni)** samples with CHCl_3_ or CH_2_Cl_2_ for a couple of days resulted in noticeable PXRD pattern changes, which were associated with a slow phase transformation to **WUT-2­(Ni)** (Figure S12 and S13) (vide infra). While the immersion of **WUT-2­(Ni)** in solvents such as acetonitrile, hexane, or water for 2 weeks caused no alterations in its crystal structure, exposing **WUT-2­(Ni)** to EtOH, acetone, THF, or toluene led to the appearance of distinct reflections characteristic of **WUT-1­(Ni)** (Figures S15b–S17). Cluster **1** exhibits good thermal stability, which was evidenced by thermogravimetric analysis (TGA) and variable temperature PXRD (VT-PXRD) analyses. Heating this material to 200 °C resulted in a 30.6% weight loss related to the release of the residual solvent (DMF) from the voids without collapsing the diamondoid **WUT-1­(Ni)** lattice (Figures 2a and S20). This value was in good agreement with the SC-XRD experiment, indicating an approximately 1:12 molar ratio of **1** to DMF in the as-synthesized **WUT-1­(Ni)** crystals. Further heating causes an irreversible phase transition to the **WUT-2­(Ni)** framework, which starts above 250 °C (Figures 2a and S19). The resulting material retains its crystal structure up to 400 °C and after cooling to 25 °C (Figure S19), which makes it exceptionally stable among all NPMs.

**Figure 2:**
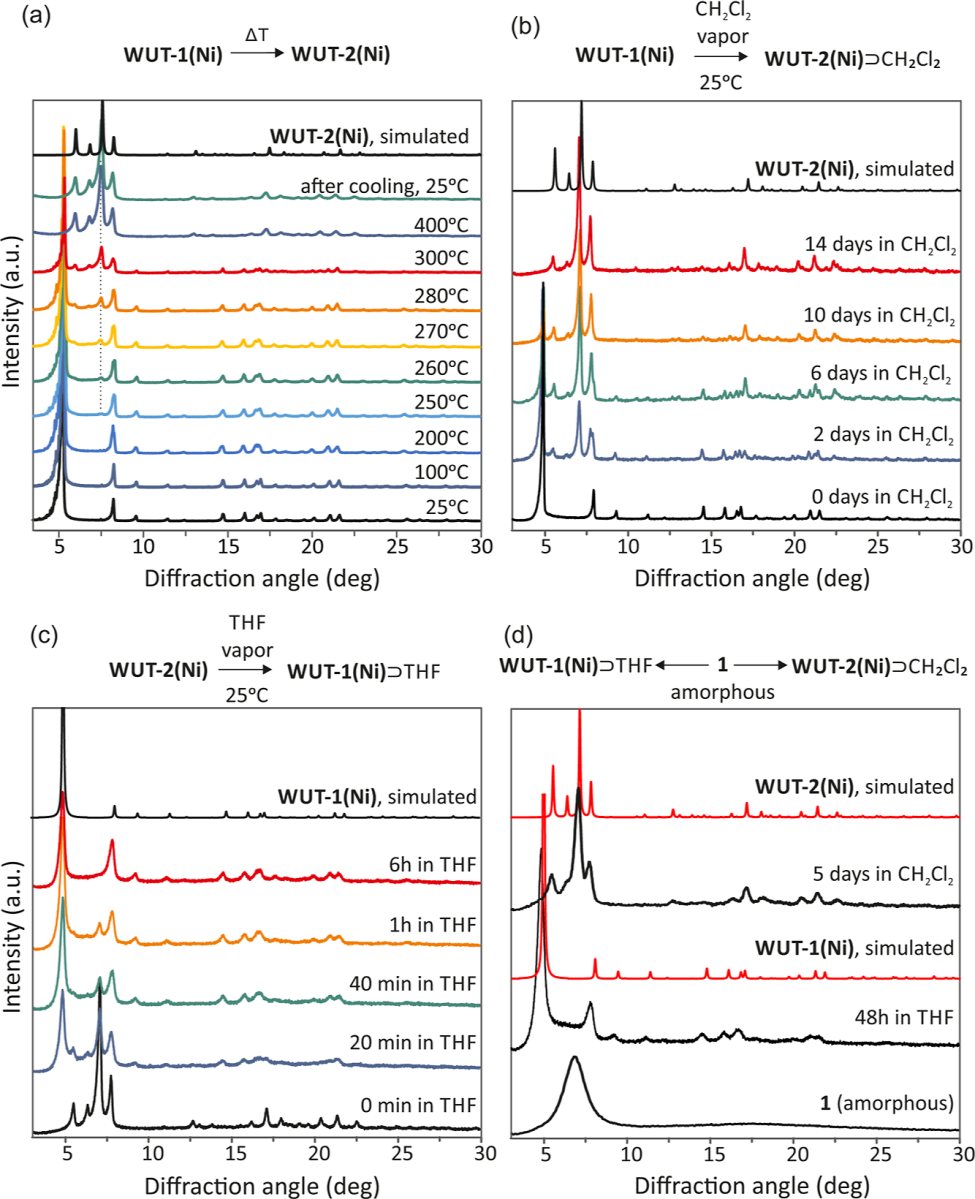
(a) Variable temperature PXRD diffraction of **WUT-1­(Ni)**. Beyond 250 °C, an irreversible phase change occurs. (b,c) PXRD patterns of phase transformation between **WUT-1­(Ni)** and **WUT-2­(Ni)** upon THF or CH_2_Cl_2_ vapor treatment. (d) PXRD profiles of an amorphous sample of **1** after exposure to THF and CH_2_Cl_2_ vapor.

#### Thermally and Chemically Induced Phase Transitions between **WUT-1­(Ni)** and **WUT-2­(Ni)**

The experiments presented above show that cluster **1** can easily transform between **WUT-1­(Ni)** and **WUT-2­(Ni)** frameworks. Therefore, we investigated the effect of chemical and thermal stimuli on the direction of these transformations. Prolonged heating of a sample of **WUT-1­(Ni)** to 270 °C results in its complete transformation to the **WUT-2­(Ni)** framework (Figure S9), which is a single crystal to polycrystalline material transition, as evidenced by SCXRD analysis. Moreover, we have performed differential scanning calorimetry (DSC) analysis for the activated **WUT-1′(Ni)**, which revealed a broad endothermic peak during the heating in the temperature range of 204–305 °C (Figure S23). This observation is associated with the phase transition to the higher density and lower symmetry phase, which induces strains crushing the single-crystal sample and resulting in polycrystalline powder. These observations collaborate well with the PXRD data.

The thermally induced process is irreversible, and the resulting **WUT-2­(Ni)** phase is stable after being cooled to room temperature. In turn, the **WUT-1­(Ni)** → **WUT-2­(Ni)** phase transition can be easily reversed by vapor solvent treatment. Under THF vapor, we observe guest-induced structural reorganization of **WUT-2­(Ni)** and retrieve its parent **WUT-1­(Ni)** diamond framework at ambient temperature, which was evidenced by time-resolved PXRD (Figure 2c). The complete recovery of the original **WUT-1­(Ni)** phase required 6 h. On the other hand, treatment of a **WUT-1­(Ni)** sample with a CH_2_Cl_2_ vapor leads to its rearrangement into **WUT-2­(Ni)** within 14 days (Figure 2b). Remarkably, an amorphous bulk material, obtained via fine grinding of a **WUT-1­(Ni)** sample, exhibits crystallinity recovery to the **WUT-1­(Ni)** or **WUT-2­(Ni)** phase when treated by THF or CH_2_Cl_2_ vapor, respectively, at 25 °C (Figure 2d). This is a rare example of the solvent-triggered crystal self-repair process in NPMs, which usually exhibit nonreversible microporous structure collapse events.^66^ Furthermore, the phase transitions between **WUT-1­(Ni)** and **WUT-2­(Ni)** can be accelerated by soaking the crystalline samples in a selected solvent. The immersion of **WUT-2­(Ni)** in THF leads to polymorphic interconversion to the **WUT-1­(Ni)** framework within 1 h (7 days in toluene) (Figures S16 and S17), while immersion of **WUT-1­(Ni)** in CH_2_Cl_2_ leads to its rearrangement into **WUT-2­(Ni)** in 14 days (7 days in CHCl_3_) (Figures S12 and S13). Due to the absolute insolubility of **1** in THF or CH_2_Cl_2_, it is unlikely that the observed transformations occur via the dissolution–recrystallization process.

### Adsorption Properties of **WUT-1­(Ni)** and **WUT-2­(Ni)**

We examined the gas adsorption properties of **WUT-1­(Ni)** and **WUT-2­(Ni)** experimentally and computationally, as molecular simulations are useful tools for investigating the porous properties of structurally adaptable molecular systems. The as-synthesized **WUT-1­(Ni)** material was activated by a supercritical process employing liquid carbon dioxide and dried under high vacuum (for details, see Supporting Information). Alternatively, a sample of **WUT-1­(Ni)** can be washed three times with ethanol and heated under high vacuum at 110 °C. In turn, **WUT-2­(Ni)** material obtained by thermal transformation of **WUT-1­(Ni)** was activated by heating the sample at 160 °C under high vacuum for 16 h. The crystallinity and phase identity of the as-prepared activated materials, further denoted as **WUT-1′(Ni)** and **WUT-2′(Ni)**, were confirmed by PXRD analysis (Figures S10 and S14) and TGA measurements (Figures S21 and S22), which showed the successful removal of the residual solvent molecules. Samples of **WUT-1′(Ni)** and **WUT-2′(Ni)** were used in adsorption experiments of N_2_, H_2_, CH_4_, and CO_2_ under pressures up to 1 and 10 bar. In parallel, we used grand canonical Monte Carlo (GCMC) simulations to computationally calculate the corresponding isotherms.

The details of the simulations are provided in the Supporting Information. Importantly, the structures used for the simulations are assumed rigid and thus provide a good reference point to understand the potential flexible structures. Interestingly, the results show that **WUT-1′(Ni)** exhibits significantly enhanced affinity to CO_2_ in comparison to that observed previously for the isostructural **WUT-1′(Zn)** ^61^ material, despite the similar porosity of both materials.

#### **WUT-1′(Ni)** Nitrogen Adsorption

The N_2_ adsorption isotherm at 77 K of **WUT-1′(Ni)** shows, similarly to **WUT-1′(Zn)**, the general Type I character of microporous materials with no hysteresis and a complex shape at low pressure indicating the stepwise filling of different pores and adsorption sites (Figure 3a). Figure 3b shows snapshots of the simulated isotherms at three different loadings. The measured **WUT-1′(Ni)** saturation uptake of N_2_ at 77 K is 335 cm^3^ g^–1^ at *p*/*p*_0_ = 0.99, and the calculated BET area is 1322 m^2^ g^–1^ (calculated using BETSI^67^), with a total pore volume of 0.51 cm^3^ g^–1^ (Figure S29). These are comparable to the values obtained for **WUT-1′(Zn)** (1225 m^2^ g^–1^ and 0.48 cm^3^ g^–1^). The simulated isotherm matches well the experimental shape but underpredicts the final uptake for *p*/*p*_0_ values above 10^–3^. The semilog-scale subplot shows the simulated and experimental isotherms match perfectly in shape and values below *p*/*p*_0_ = 10^–3^, indicating the model used for the simulations correctly captures the interactions between N_2_ and **WUT-1′(Ni)**. However, above 10^–3^, the experimental isotherm increases to reach a plateau higher than the calculated one. It is usually expected that the simulated isotherm overpredicts the experimental isotherm, as the simulations are performed on ideal structures, whereas experimental materials can contain defects or residual solvents. It has been shown in the MOF field that underprediction of simulations implies either missing linkers in the structure or a flexible material.^68^ In the present case, a missing cluster introduces mesoporosity, which is not observed in the experimental isotherm. Therefore, the underprediction could be due to an adaptability of **WUT-1′(Ni)** (due to the lack of strong interactions between clusters, one may expect some flexibility of the title network; however, control PXRD experiments for **WUT-1′(Ni)** at 195 K under vacuum and CO_2_ atmosphere did not provide further evidence of the phase transition upon gas adsorption, see Figure S18). Importantly, both the simulated and experimental isotherms suggest a filling behavior similar to the one observed previously for **WUT-1′(Zn)**: the nitrogen molecules first occupy the narrow pores within each molecule of **WUT-1′(Ni)** (step I) before being adsorbed between the clusters and within the central pore of each discrete molecule (step II) and finally saturating all the cavities (step III). Snapshots and the pore size distribution (PSD) were obtained computationally (Figures 3b and S22) and suggest that the pores of sizes below 10 Å are filled during step I and the pores of sizes around 10 Å are filled during steps II and III. The simulated and experimental isotherms match well in step II and differentiate in step III at *p*/*p*_0_ = 10^–3^.

**Figure 3:**
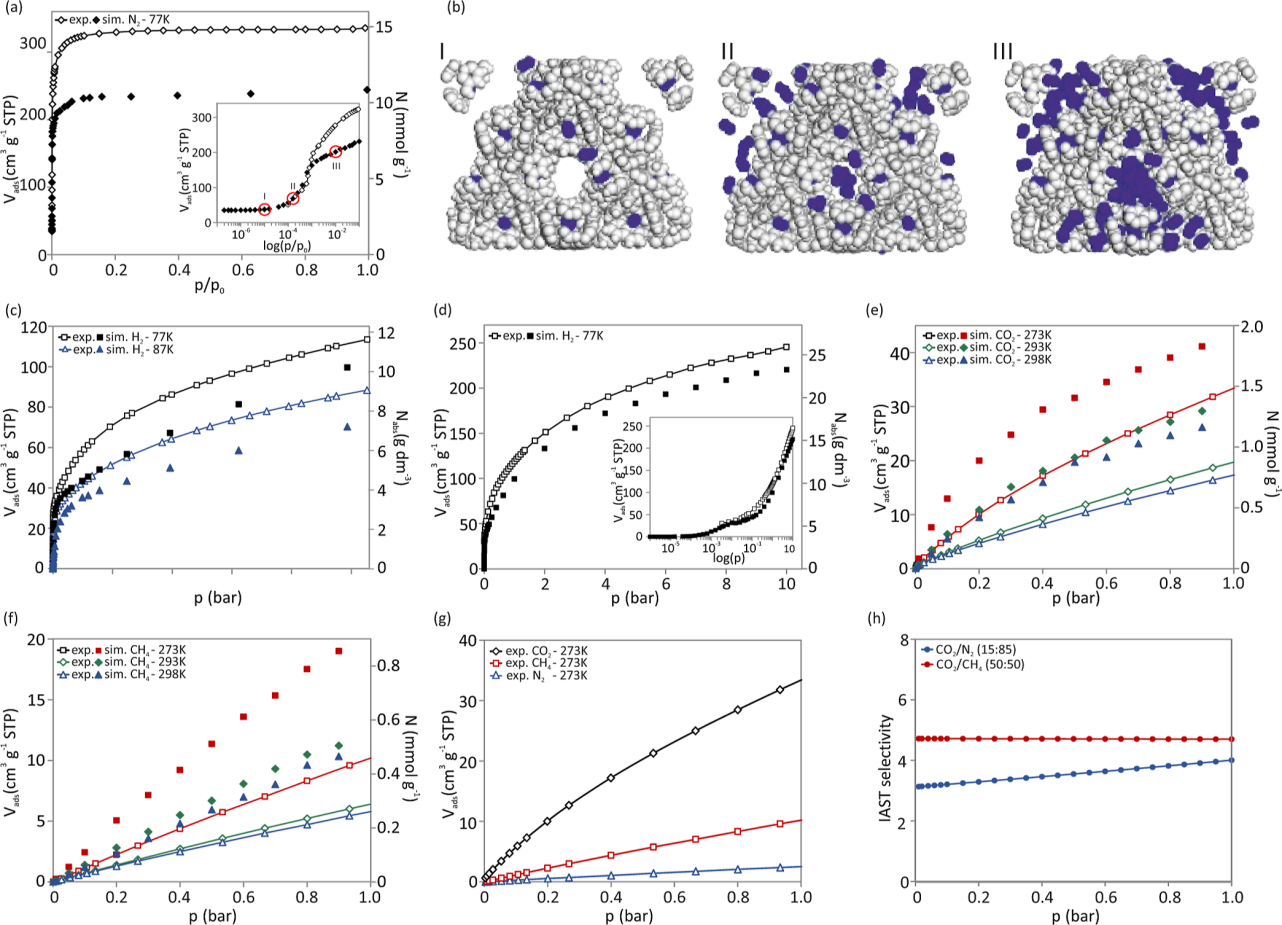
(a) Experimental (open symbols) and simulated (closed symbols) N_2_ adsorption isotherms for **WUT-1′(Ni)** in 77 K up to 1 bar using standard and semilogarithmic scales; circled data points indicate the isotherm points for the snapshots. (b) The adsorption mechanism of N_2_ on **WUT-1′(Ni)** at 77 K using snapshots at three different loadings, as indicated on the semilogarithmic-scale isotherm. Experimental (open symbols) and simulated (closed symbols) adsorption isotherms: (c) H_2_ up to 1 bar at 77 and 87 K; (d) H_2_ isotherm up to 10 bar at 77 K using standard and semilogarithmic scales; (e) CO_2_ at 273, 293, and 298 K; (f) CH_4_ at 273, 293, and 298 K; (g) CO_2_, CH_4_, and N_2_ adsorption isotherms at 273 K under 1 bar. (h) IAST selectivity curves of the binary mixtures: CO_2_/N_2_ (15:85) and CO_2_/CH_4_ (50:50) at 273 K and 1 bar.

#### **WUT-1′(Ni)** Hydrogen Adsorption

Evaluation of the H_2_ sorption shows that **WUT-1′(Ni)** exhibits sorption abilities similar to those of **WUT-1′(Zn)** at 1.1 bar, with an adsorption capacity of 116 cm^3^ g^–1^ (1.04 wt %, 12.1 g L^–1^, 77 K). However, at the higher pressure of 10 bar, the adsorption capacity of **WUT-1′(Ni)** is significantly higher than that observed for **WUT-1′(Zn)** (200 cm^3^ g^–1^), reaching 246 cm^3^ g^–1^ (2.16 wt %, 25.9 g L^–1^, 77 K) (Figure 3c,d). The volumetric H_2_ adsorption values are among the highest reported for NPM materials.^39^ The simulated isotherm matches once again the experimental shape but slightly underpredicts the uptake. The semilog subplot shows a step around 10^–3^ bar, where the simulated and measured isotherms match almost perfectly. The good predictions at low pressures suggest that underprediction at higher pressures could be due to structural adaptability.

#### **WUT-1′(Ni)** CO_2_ Adsorption

Similarly, **WUT-1′(Ni)** shows a high affinity toward CO_2_. The total uptake of CO_2_ at 195 K reaches 247.8 cm^3^ g^–1^ (11.0 mmol g^–1^, 1 bar), which is a slightly higher value compared to **WUT-1′(Zn)**’s with an uptake of 235 cm^3^ g^–1^ (Figure S28). Furthermore, **WUT-1′(Ni)** displays notable CO_2_ adsorption amounts at higher temperatures as well, with 35.0 cm^3^ g^–1^ (1.6 mmol g^–1^, 1 bar) and 153 cm^3^ g^–1^ (6.8 mmol g^–1^, 10 bar) at 273 K, and 18.2 cm^3^ g^–1^ (0.8 mmol g^–1^, 1 bar) and 124 cm^3^ g^–1^ (5.6 mmol g^–1^,10 bar) at 298 K (Figures 3e and S38). These values are significantly higher than those of **WUT-1′(Zn)** (for example, CO_2_ uptake in **WUT-1′(Zn)** is 24.4 cm^3^ g^–1^ at 273 K, 1 bar, which is about 43% less than in **WUT-1′(Ni)**), which shows that the exchange of metal ions in isoreticular frameworks can significantly affect the interactions with guest molecules. This is very interesting considering that both materials are composed of molecules with coordinatively saturated metal centers that likely do not directly participate in the binding of guest molecules. A more in-depth analysis of the CO_2_ uptakes at low temperatures reveals a characteristic stepwise adsorption isotherm that likely results from the gradual filling of different sorption sites in **WUT-1′(Ni)** (Figure S28), which was also observed in **WUT-1′(Zn)**. The simulated isotherms capture the shape of the experimental isotherms and the difference in uptakes at the different temperatures but overpredict the overall uptakes. This is usually to be expected, but the pressure range used here does not allow us to conclude whether this is due to overestimated molecular interactions or differing pore volumes.

#### **WUT-1′(Ni)** Methane Uptake

The CH_4_ uptake reaches 6.1 cm^3^ g^–1^ (0.27 mmol g^–1^, 1 bar) and 42 cm^3^ g^–1^ (1.86 mmol g^–1^, 10 bar) at 298 K (Figures 3f and S39) and only 2.7 cm^3^ g^–1^ (0.12 mmol g^–1^, 1 bar) for N_2_ at 273 K and 1.6 cm^3^ g^–1^ (0.07 mmol g^–1^, 1 bar) at 298 K (Figures 3g and S32). The simulated isotherms once again capture the right shape and uptake difference but overpredict the overall uptakes. Similarly to the CO_2_ case, it is not clear whether this is due to the interactions model or differences in pore volume.

#### **WUT-1′(Ni)** Water Uptake

PXRD analysis confirmed the excellent stability of the **WUT-1­(Ni)** crystalline structure in the presence of water (Figure S10). To further evaluate its performance under humid conditions, water adsorption/desorption measurements were conducted at 298 K on the activated **WUT-1′(Ni)** material. The adsorption isotherm of **WUT-1′(Ni)** follows type V behavior, indicative of hydrophobic channels, and exhibits a low water uptake of 10.87 cm^3^ g^–1^ (4.86 mg g^–1^) at a pressure of 0.6 bar (Figure S33). Such small water adsorption is highly advantageous for gas separation applications in humid environments, as it reduces competitive adsorption effects and preserves the material’s selectivity. Notably, the framework retained its structural postadsorption stability, as confirmed by PXRD (Figure S35).

#### Gas Adsorption Selectivity in **WUT-1′(Ni)**

**WUT-1′(Ni)**’s significant superior affinity to CO_2_ over CH_4_ and N_2_ was also confirmed by the isosteric heats of adsorption (*Q*_st_) calculated from the virial equation. The *Q*_st_ values at zero coverage are 26.1, 14.9, and 18.1 kJ mol^–1^ for CO_2_, CH_4_, and N_2_, respectively (Figure S48). The calculated value of *Q*_st_ for CO_2_ adsorption at zero coverage is slightly higher than the one observed for **WUT-1′(Zn)** (24.5 kJ mol^–1^), and gradually decreases to 22.4 kJ mol^–1^ at 1.0 mmol g^–1^ loading of CO_2_. Here, we draw the reader’s attention to the isosteric heat of adsorption of H_2_: with an initial high value of 12.2 kJ mol^–1^, it gradually decreases to 10.1 kJmol^–1^ at 1 mmol g^–1^ loading of H_2_. Importantly, such high *Q*_st_ values have been observed in **WUT-1­(Zn)** and also in MOFs with coordinatively unsaturated metal sites, whereas values of 3.8–9.5 kJ mol^–1^ are typically reported for MOFs without open metal sites.^69^ To estimate the potential separation capability of **WUT-1′(Ni)**, we applied the Ideal Adsorbed Solution Theory (IAST) method combined with the Langmuir–Freundlich fitting based upon the experimental pure component isotherms at 273 and 298 K (Table S7, Figure S49). Calculated selectivities at 273 K and 1 bar in the CO_2_/CH_4_ (50:50) and CO_2_/N_2_ (15:85) mixtures are 4.7 and 4.0, respectively (Figure 3h).

Notably, the **WUT-1′(Ni)** framework exhibited excellent stability during adsorption processes, as demonstrated by multiple cycles of N_2_ adsorption–desorption experiments at 77 K (Figure S34). The material largely retained its adsorption properties after immersion in water or buffer solutions at pH 7 and 10 for 24 h, with no change in BET surface area (Figures S35 and S36). However, exposure to a pH 4 buffer resulted in a slight decrease in BET surface area, approximately 10%, indicating a minor impact on adsorption capacity under more acidic conditions.

#### **WUT-2′(Ni)** Nitrogen Adsorption

The N_2_ sorption isotherm of the **WUT-2′(Ni)** measured at 77 K shows Type II character with a saturation uptake of 190 cm^3^ g^–1^ (8.5 mmol g^–1^, 1 bar) (Figure 4a). This type of isotherm is characteristic of nonporous materials, which suggests that the closed cavities within the **WUT-2′(Ni)** framework are inaccessible for N_2_ molecules. However, diffusion is not taken into account in GCMC simulations: all pores are assumed to be accessible by default assumed accessible. Therefore, the resulting isotherm is Type I and does not match the experimental data. The BET area derived from the experimental N_2_ adsorption isotherm is 94 m^2^ g^–1^, with a total pore volume of 0.21 cm^3^ g^–1^ (Figure S42). To consider the inaccessibility of certain pockets in **WUT-2′(Ni)** in simulations, we computationally mapped out the structure’s pores and porous channels and identified pockets that, due to their kinetic diameters, are physically inaccessible by H_2_, CO_2_, and CH_4_. We then artificially blocked these pockets.

**Figure 4:**
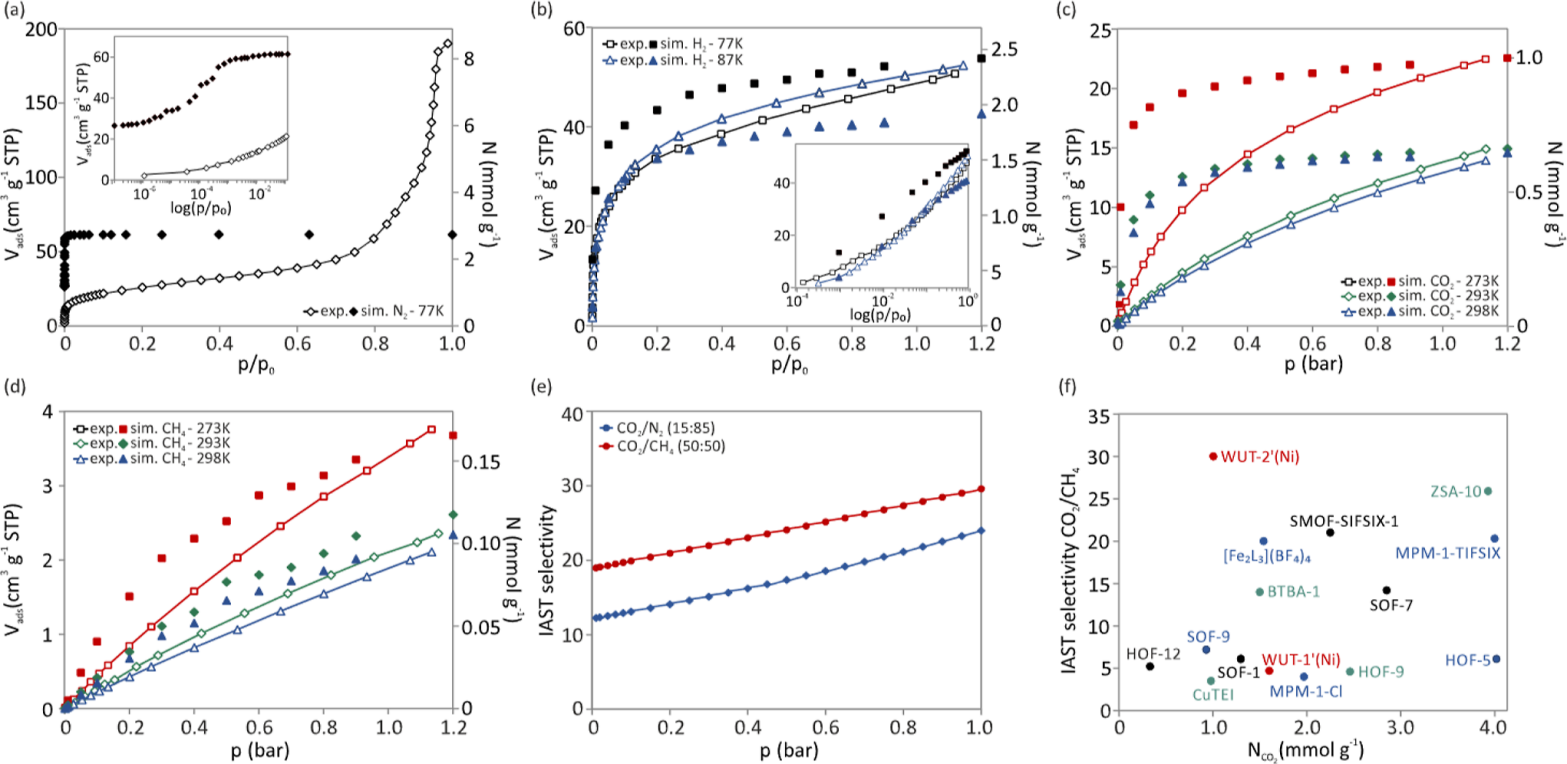
Experimental (open symbols) and simulated (closed symbols) data for **WUT-2′(Ni)**: (a) N_2_ adsorption in 77 K up to 1 bar using standard and semilogarithmic scales; (b) H_2_ adsorption isotherms at 77 and 87 K; (c) CO_2_ at 273, 293, and 298 K; (d) CH_4_ at 273, 293, and 298 K. (e) IAST selectivity curves of the binary mixtures: CO_2_/N_2_ (15:85) and CO_2_/CH_4_ (50:50) at 273 K and 1.0 bar; (f) comparison of CO_2_/CH_4_ (50:50) selectivity at 273 and 298 K under 1 bar for NPM materials.^21,28,52–54,70–75^

#### **WUT-2′(Ni)** Hydrogen Adsorption

The H_2_ uptakes at 77 K and 87 K are 50 cm^3^ g^–1^ (2.26 mmol g^–1^, 1.1 bar) and 52 cm^3^ g^–1^ (2.33 mmol g^–1^, 1.1 bar) (Figure 4b), respectively, which shows that H_2_ penetrates the **WUT-2′(Ni)** framework better than N_2_ at this temperature. The simulated isotherm at 77 K reproduces the shape of the experimental isotherm but overpredicts the experimental uptakes over the entire pressure range. The lower experimentally measured uptake could be due to kinetic effects. On the other hand, the simulated isotherm at 87 K reproduces the shape of the experimental one, with a perfect match below *p*/*p*_0_ = 10^–3^. Above 10^–3^, the simulated isotherm underpredicts again the experimental uptake, which could be due to the presence of mesoporosity. Interestingly, the calculated uptakes at 87 K are lower than those at 77 K, whereas it is the opposite for the measured uptakes. The increased measured uptake of H_2_ with temperature likely indicates that access to the separated voids is enabled by thermal vibrations of the molecules, which is associated with a flexible behavior of the crystal lattice of **WUT-2′(Ni)**. Notably, the **WUT-2′(Ni)** framework remains stable during adsorption processes, as demonstrated by multiple cycles of H_2_ adsorption–desorption experiments at 77 K (Figure S45).

#### **WUT-2′(Ni)** CO_2_ and CH_4_ Adsorption

The CO_2_ uptake capacity for **WUT-2′(Ni)** up to 1 bar was estimated at 22.0 cm^3^ g^–1^ (0.98 mmol g^–1^, 273 K), 14.3 cm^3^ g^–1^ (0.64 mmol g^–1^, 293 K), and 13.4 cm^3^ g^–1^ (0.60 mmol g^–1^, 298 K) (Figure 4c), while the CH_4_ adsorption amounts are 3.6 cm^3^ g^–1^ (0.16 mmol g^–1^, 273 K), 2.2 cm^3^ g^–1^ (0.10 mmol g^–1^, 293 K), and 2.0 cm^3^ g^–1^ (0.09 mmol g^–1^, 298 K) (Figure 4d). The simulated isotherms predict the same uptakes at high pressures but steeper curves at lower pressures, which indicates that the interactions between the CO_2_ and CH_4_ molecules and the structure are overestimated.

#### Gas Adsorption Selectivity in **WUT-2′(Ni)**

**WUT-2′(Ni)** shows relatively low adsorption amounts of N_2_: 1.1 cm^3^ g^–1^ (0.05 mmol g^–1^, 1 bar) at 273 K and 0.6 cm^3^ g^–1^ (0.02 mmol g^–1^, 1 bar) at 298 K (Figure S44). The heats of adsorption *Q*_st_ for CH_4_, N_2_, and H_2_ in **WUT-2′(Ni)** at zero coverage were estimated to be 19.5, 20.1, and 7.2 kJmol^–1^, respectively (Figure S48). The heats of adsorption *Q*_st_ for CO_2_ uptakes in **WUT-2′(Ni)** were calculated to be a moderate 34.6 kJmol^–1^ at zero coverage and then gradually decreased to 30.5 kJmol^–1^ at about 1.0 mmol·g^–1^ loading of CO_2_. This value is much higher than those in **WUT-1′(Ni)** and **WUT-1′(Zn)**, which indicates the strong interactions between CO_2_ and the pore surface.

The calculated gas adsorption selectivities are much higher for **WUT-2′(Ni)** than for **WUT-1′(Ni)**. Particularly, CO_2_/CH_4_ (50:50) and CO_2_/N_2_ (15:85) IAST selectivities at 273 K for **WUT-2′(Ni)** are 29.6 and 24.0, six times higher than those of **WUT-1′(Ni)** (Figure 4e and Table S7). Indeed, the CO_2_/CH_4_ IAST selectivity in **WUT-2′(Ni)** is the highest for the CO_2_/CH_4_ mixture for all NPMs based on metal clusters at ambient conditions (Figure 4f, Table S8). In turn, the CO_2_/N_2_ selectivity is comparable with previously reported NPMs and MOFs under similar conditions, such as MPM-1-Cl,^28^ HOF-5,^74^ HOF-9,^75^ ZIF-79,^76^ Ni-MOF-74,^77^ and SIFSIX-2-Cu^78^ (Table S8). The increased gas adsorption selectivities in **WUT-2′(Ni)** compared to **WUT-1′(Ni)** likely result from much tighter closed voids, which facilitate selective interactions with gas molecules. Transient breakthrough simulations were also conducted to evaluate the feasibility of using **WUT-1­(Ni)** and **WUT-2­(Ni)** in a fixed bed for the separation of CO_2_/N_2_ and CO_2_/CH_4_ mixtures (Figure S51). Both materials demonstrate selective CO_2_ adsorption with CO_2_ breaking through significantly later than did N_2_ or CH_4_.

## Conclusions

In summary, we synthesized and characterized the novel decanuclear Ni­(II) carbonato nanocluster **1** featuring a hydroxyquinolinato shell, which acts as building blocks capable of forming completely different self-assembly modes: diamondoid **WUT-1­(Ni)** and pyrite **WUT-2­(Ni)**, depending on the processing conditions. For example, the transitions between both polymorphs can be selectively triggered by the temperature or exposure to a particular organic solvent. Interestingly, the solvent-assisted transformations are likely of a nature different from that of a simple dissolution–recrystallization process, and they are accompanied by the recovery of crystallinity by the bulk materials without passing through a melting or dissolving state. Both developed microporous molecular solids exhibit excellent thermal and chemical stability under aerobic as well as aqueous conditions and demonstrate interesting gas adsorption properties. Indeed, **WUT-1­(Ni)** shows one of the highest H_2_ uptakes among NPMs and almost two times higher CO_2_ uptakes than that of the previously reported isostructural Zn­(II) analogue **WUT-1­(Zn)**.^61^ In turn, tighter closed voids of the ultramicroporous **WUT-2­(Ni)** framework facilitate selective interactions with gas molecules, which results in outstanding selectivity in the adsorption of CO_2_ over N_2_ and CH_4_, making this material a good candidate for the separation of CO_2_ from flue gas to mitigate environmental pollution and biogas purification.

Control over the noncovalent interaction-driven self-assembly of molecular blocks, ensuring permanent porosity of the resulting crystal lattices, is still a great challenge, especially in the case of complicated and dynamic hybrid organic–inorganic systems. Most of the studies on the self-assembly of metal complexes were focused on the manipulations of ligands in the secondary coordination sphere, which directly participates in intermolecular interactions.^79^ In turn, our results demonstrated the profound role of the character of metal centers on both self-assembly modes of hydroxyquinolinato-carbonato [M_10_(μ_6_-CO_3_)_4_(L)_12_]-type clusters and the properties of the resulting microporous frameworks. While the **WUT-1**- and **WUT-2**-type frameworks were previously isolated exclusively for Zn­(II)^61^ and Co­(II) and Mn­(II)^63,64^ clusters, respectively, the herein presented Ni­(II) analogue exhibits supramolecular structure versatility with selective intertransitions between both supramolecular phases. Moreover, a simple substitution of Zn­(II) by Ni­(II) in isostructural clusters not only increased their chemical stability but also significantly enhanced the interactions of the **WUT-1**-type framework with gas molecules, which is highly intriguing considering the coordinative structuration of the metal centers that likely do not directly participate in the binding of guest molecules. Moving forward, the insight gained should aid in the development of advanced porous solid-state materials.

## Supplementary Material



## Notes

Materials, synthesis and characterization methods, experimental details of SC-XRD, PXRD, and VT PXRD studies, IR spectra, TGA and DSC data, SEM images, sorption data, isotherm fit parameters, heat of adsorption calculations, computational GCMC simulation, IAST simulation, and breakthrough simulations (PDF)
