# Supplementary material for: Tunable Self-Assembly of Decanuclear Ni(II) Carbonato Clusters with a Hydroxyquinolinato Shell: Robust Porous Networks with Reversible Solvent-/Temperature-Driven Phase Transitions and Selective Gas Separation
Source: J Am Chem Soc. 2025 May 27;147(22):19073–83. doi: 10.1021/jacs.5c04096 (PMC12147118; doi:10.1021/jacs.5c04096)
Supplement: Supplementary file 1 [file ja5c04096_si_001.pdf]

Supporting Information

Tunable self-assembly of decanuclear Ni(II) carbonato clusters with hydroxyquinolinato shell: Robust porous networks with reversible solvent/temperature-driven phase transitions and selective gas separation

Katarzyna Sołtys-Brzostek[†], Kamil Sokołowski[†], Iwona Justyniak[†], Aurelia Li[‡], David Fairen-Jimenez[‡*], Alicja Supeł[§], Michał Terlecki[§], and Janusz Lewiński[†,§*]

[†] Institute of Physical Chemistry, Polish Academy of Sciences, Kasprzaka 44/52, Warsaw 01-224 (Poland)

[‡] The Adsorption & Advanced Materials Laboratory (A^{2}ML), Department of Chemical Engineering & Biotechnology, University of Cambridge, Philippa Fawcett Drive, Cambridge CB3 0AS (United Kingdom)

[§] Faculty of Chemistry, Warsaw University of Technology, Noakowskiego 3, Warsaw 00-664 (Poland)

[*] Correspondence to Janusz Lewiński (janusz.lewinski@pw.edu.pl)

Table of contents

1. Experimental Section S2
2. FTIR S3
3. Single Crystal X-Ray Diffraction S4
4. Powder X-Ray Diffraction Analysis S10
5. Variable-temperature Powder X-ray Diffraction Analysis S18
6. Thermal Analysis S19
7. Differential scanning calorimetry of WUT-1'(Ni) S20
8. Optical Microscope images S21
9. Scanning Electron Microscopy S22
10. Gas adsorption experiments and adsorption simulations S23
11. The isosteric heats of adsorption S35
12. Ideal Adsorbed Solution Theory calculations S39
13. Breakthrough simulations S42
14. Adsorption Information Files S44

1. Experimental Section

Materials: 8-hydroxyquinoline (Sigma Aldrich), $Ni(CH_3COO)_2{\cdot}4H_2O$ (POCH), $N(CH_3)_4OH{\cdot}5H_2O$ (TCI) were used without further purification; NaOH (POCH, 0,35M solution in water), CO_2 (Multax, ≥ 99,99%). Solvents: N,N-Dimethylformamide anhydrous, dried over molecular sieves 4Å (POCH), dichloromethane (POCH), THF (POCH).

Synthesis of WUT-1(Ni):

Method 1: $Ni(CH_3COO)_2{\cdot}4H_2O$ (0.026 g, 0.124 mmol) was added to a solution of 8-hydroxyquinoline (0.018 g, 0.124 mmol) in DMF (1 mL) at room temperature. Then, a solution of NaOH in water (0.05 mL, 0.34 M) was added to the reaction mixture, and the solution was stirred for an additional 15 minutes. The obtained suspension was filtered and diluted in 1 mL of DMF. The resultant solution was exposed to a CO_2 atmosphere at ambient temperature without stirring. Compound **WUT-1(Ni)** was obtained as green crystals after crystallization from the parent mixture at room temperature after 1 day. The obtained microcrystalline solid was decanted and washed three times with DMF (1 mL) and THF (2 mL). The washed product was dried under a vacuum. Product **WUT-1(Ni)** is stable in the air atmosphere, with an isolated yield of 50%. Elemental analysis (%) calc for $C_{112}H_{72}N_{12}O_{24}Ni_{10}{\cdot}9.4C_3H_7NO$ (3244.0 g mol^{-1}): C 51.91, H 4.28, N 9.24, O 16.47, Ni 18.10 (%). Found: C 54.90, H 4.59, N 9.87 (%).

Method 2: $N(CH_3)_4OH{\cdot}5H_2O$ (0.018 g, 0.124 mmol) was added to a solution of 8-hydroxyquinoline (0.018 g, 0.124 mmol) in DMF (3 mL) at room temperature. The reaction was carried out for 20 minutes until the base had reacted completely. Then, a solution of $Ni(CH_3COO)_2{\cdot}4H_2O$ (0.026 g, 0.104 mmol) in DMF (1 mL) was added to the reaction mixture and filtered. The resultant solution was exposed to a CO_2 atmosphere at ambient temperature without stirring. Compound **WUT-1(Ni)** was obtained as green crystals after crystallization from the parent mixture at room temperature after 1 day. The obtained microcrystalline solid was decanted and washed three times with DMF (1 mL) and THF (2 mL). The washed product was dried under a vacuum. Product **WUT-1(Ni)** is stable in the air atmosphere, with an isolated yield of 75%.

Preparation of activated WUT-1'(Ni):

The as-synthesized sample of **WUT-1(Ni)** was activated using supercritical CO_2. Elemental analysis (%) calc for $C_{112}H_{72}N_{12}O_{24}Ni_{10}$ (2556.8 g mol^{-1}): C 52.61, H 2.84, N 6.57, O 15.02, Ni 22.96 (%). Found: C 53.23, H 2.74, N 6.76 (%).

Synthesis of a WUT-1(Ni)/WUT-2(Ni) mixture:

$Ni(CH_3COO)_2{\cdot}4H_2O$ (0.026 g, 0.104 mmol) was added to a solution of 8-hydroxyquinoline (0.018 g, 0.124 mmol) in DMF (1 mL) at room temperature. Then, a solution of NaOH in water (0,33 mL, 0.34 M) was added to the reaction mixture, and the solution was stirred for an additional 15 minutes. The obtained suspension was filtered and diluted in 1 mL of DMF. The resultant solution was exposed to a CO_2 atmosphere at ambient temperature without stirring. The mixture of two crystal phases: **WUT-2(Ni)** and **WUT-1(Ni),** with different crystallite shapes, were obtained after crystallization from the parent mixture at room temperature for

2 days. The obtained microcrystalline solid was decanted and washed three times with DMF (1 mL) and THF (2 mL). The washed product was dried under vacuum.

Preparation of pure WUT-2(Ni) phase:
The freshly synthesized **WUT-1(Ni)** material (200 mg) was washed three times with DMF (1 mL) and dried under vacuum. Next, it was heated at 270°C under atmospheric pressure for 20 h, leading to **WUT-2(Ni)** in quantitative yield. The phase purity of the resulting product was confirmed by PXRD analysis.

Preparation of activated WUT-2'(Ni):
The sample of **WUT-2(Ni)** was heated at 160°C under high vacuum for 16 h to obtain a fully evacuated framework of **WUT-2'(Ni)**. Alternatively, a sample of WUT-1(Ni) can be directly transformed to **WUT-2'(Ni)** by heating at 270°C under high vacuum for 20 h. Elemental analysis (%) calc for $C_{112}H_{72}N_{12}O_{24}Ni_{10}$ (2556.8 g mol^{-1}): C 52.61, H 2.84, N 6.57, O 15.02, Ni 22.96 (%). Found: C 53.27, H 2.76, N 6.79 (%).

2. FTIR

Fourier Transform Infrared Attenuated Total Reflectance (FTIR ATR) were recorded (500-4000 cm^{-1} region) on a Bruker Tensor apparatus equipped with an ATR accessory.

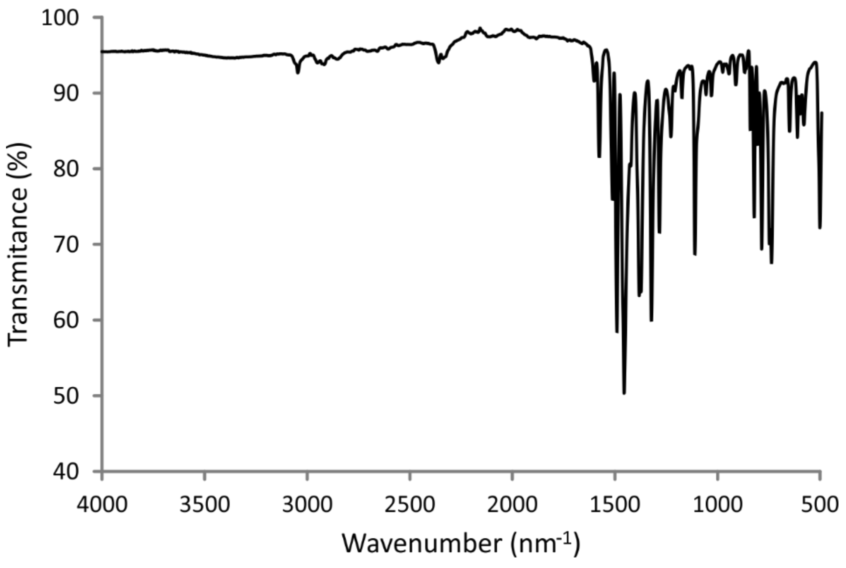

Figure S1. FT-IR spectrum of **WUT-1(Ni)**.

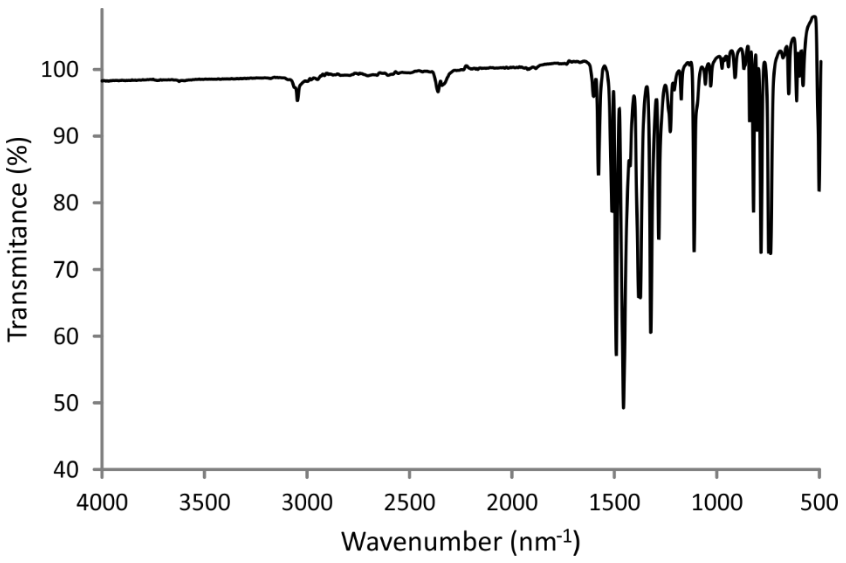

Figure S2. FT-IR spectrum of activated **WUT-2(Ni)**.

3. Single Crystal X-Ray Diffraction

The crystals of all complexes were selected under Paratone-N oil, mounted on the nylon loops and positioned in the cold stream on the diffractometer. The X-ray data for complexes **WUT-1(Ni), WUT-1'(Ni), WUT-2(Ni) and WUT-2'(Ni)** were collected at 100(2)K on a SuperNova Agilent diffractometer using graphite monochromated Mo$K\alpha$ radiation (λ = 0.71073 Å). The data were processed with CrysAlisPro.[1] The structures **WUT-1(Ni), WUT-1'(Ni), WUT-2(Ni)** and **WUT-2'(Ni)** were solved by direct methods using the SHELXT program and were refined by full matrix least–squares on F^2 using the program SHELXL[2] implemented in the OLEX2[3] or WinGX[4] suite**.** All non-hydrogen atoms were refined with anisotropic displacement parameters. Hydrogen atoms were added to the structure model at geometrically idealised coordinates and refined as riding atoms.

For the **WUT-1(Ni)** structure, the solvent was partially modeled using a restrained DMF model molecule localized at a special position with 3-fold symmetry. The remaining solvent content was estimated based on the squeezed results. A solvent mask was calculated, revealing 2631 electrons in a volume of 9222 Å^3 in one void per unit cell. This indicates the presence of an additional 64 DMF molecules per unit cell, accounting for 2560 electrons per unit cell. The resulting molecular formula for **WUT-1(Ni)** is **1**·12DMF.For the **WUT-2(Ni)** structure, the solvent was refined with partial occupancy, yielding the final molecular formula of **1**·0.8DMF. Crystallographic data (excluding structure factors) for the structure reported in this paper have been deposited with the Cambridge Crystallographic Data Centre as a supplementary publication. Copies of the data can be obtained free of charge on application to CCDC, 12 Union Road, Cambridge CB21EZ, UK (fax: (+44)1223-336-033; e-mail: deposit@ccdc.cam.ac.uk). CCDC: 2372134 WUT-1(Ni); 2372136 WUT-1'(Ni), 2372137 WUT-2(Ni); 2372138 WUT-2'(Ni).

Table S1. Crystallographic data and structure refinement parameters for WUT-1(Ni)

Empirical formula	$C_{112}H_{72}N_{12}Ni_{10}O_{24}$ 12(C_3H_7NO)	
Formula weight	2768.41	
Temperature	100(2) K	
Wavelength	0.71073 Å	
Crystal system	Cubic	
Space group	*F*d-3	
Unit cell dimensions	*a* = 31.1351(6) Å	*α* = 90°.
	b = 31.1351(6) Å	*β* = 90°.
	c = 31.1351(6) Å	*γ* = 90°.
Volume	30182.2(10) $Å^3$	
Z	8	
Density (calculated)	1.218 Mg/m^3	
Absorption coefficient	1.281 mm^{-1}	
F(000)	11246	
Theta range for data collection	3.400 to 26.497 °.	
Index ranges	-23<=*h*<=23, -38<=*k*<=10, -4<=*l*<=35	
Reflections collected	9156	
Independent reflections	2609 [R(int) = 0.0235]	
Completeness to theta = 25.242°	99.5 %	
Refinement method	Full-matrix least-squares on F^2	
Data / restraints / parameters	2609 / 0 / 145	
Goodness-of-fit on F^2	1.181	
Final R indices [I>2sigma(I)]	R_1 = 0.0658, wR_2 = 0.1893	
R indices (all data)	R_1 = 0.0902, wR_2 = 0.2214	
Largest diff. peak and hole	0.893 and -0.504 e.$Å^{-3}$	

Table S2. Crystallographic data and structure refinement parameters for WUT-1'(Ni)

Empirical formula	$C_{112}H_{72}N_{12}Ni_{10}O_{24}$	
Formula weight	2556.91	
Temperature	100(2) K	
Wavelength	0.71073 Å	
Crystal system	Cubic	
Space group	*F*d-3	
Unit cell dimensions	*a* = 31.1351(6) Å	*α* = 90°.
	b = 31.1351(6) Å	*β* = 90°.
	c = 31.1351(6) Å	*γ* = 90°.
Volume	30182.2(17) $Å^3$	
Z	8	
Density (calculated)	1.125 Mg/m^3	
Absorption coefficient	1.274 mm^{-1}	

F(000)	10400
Theta range for data collection	3.400 to 28.822°.
Index ranges	-23<=*h*<=23, -38<=*k*<=10, -4<=*l*<=35
Reflections collected	9697
Independent reflections	2836 [R(int) = 0.0242]
Completeness to theta = 25.242°	99.4 %
Refinement method	Full-matrix least-squares on F^2
Data / restraints / parameters	2836 / 0 / 120
Goodness-of-fit on F^2	1.102
Final R indices [I>2sigma(I)]	R_1 = 0.0455, wR_2 = 0.1185
R indices (all data)	R_1 = 0.0686, wR_2 = 0.1310
Largest diff. peak and hole	0.631 and -0.463 e.Å^{-3}

Table S3. Crystallographic data and structure refinement parameters for WUT-2(Ni)

Empirical formula	$C_{112}H_{69}N_{12}Ni_{10}O_{24}$ 0.8(C_3H_7NO)	
Formula weight	2727.60	
Temperature	100(2) K	
Wavelength	0.71073 Å	
Crystal system	Cubic	
Space group	*P*a-3	
Unit cell dimensions	*a* = 27.7063(6) Å	*α* = 90°.
	b = 27.7063(6) Å	*β* = 90°.
	c = 27.7063(6) Å	*γ* = 90°.
Volume	21268.4(14) Å^3	
Z	8	
Density (calculated)	1.704 Mg/m^3	
Absorption coefficient	1.815 mm^{-1}	
F(000)	11136	
Theta range for data collection	3.119 to 24.496 °.	
Index ranges	-30<=*h*<=17, -37<=*k*<=14, -25<=*l*<=16	
Reflections collected	9144	
Independent reflections	5884 [R(int) = 0.0631]	
Completeness to theta = 25.242°	99.4 %	
Refinement method	Full-matrix least-squares on F^2	
Data / restraints / parameters	5884 / 77 / 563	
Goodness-of-fit on F^2	1.066	
Final R indices [I>2sigma(I)]	R_1 = 0.0701, wR_2 = 0.1606	
R indices (all data)	R_1 = 0.1121, wR_2 = 0.1811	
Largest diff. peak and hole	0.703 and -0.710 e.Å^{-3}	

Table S4. Crystallographic data and structure refinement parameters for WUT-2'(Ni)

Empirical formula	$C_{112}H_{69}N_{12}Ni_{10}O_{24}$	
Formula weight	2558.28	
Temperature	100(2) K	
Wavelength	0.71073 Å	
Crystal system	Cubic	
Space group	*P*a-3	
Unit cell dimensions	*a* = 27.7063(6) Å	α = 90°.
	b = 27.7063(6) Å	β = 90°.
	c = 27.7063(6) Å	γ = 90°.
Volume	21268.4(14) $Å^3$	
Z	8	
Density (calculated)	1.598 Mg/m^3	
Absorption coefficient	1.807 mm^{-1}	
F(000)	10411	
Theta range for data collection	3.119 to 24.496°.	
Index ranges	-30<=*h*<=17, -37<=*k*<=14, -25<=*l*<=14	
Reflections collected	5884	
Independent reflections	5884 [R(int) = 0.0631]	
Completeness to theta = 25.242°	99.6 %	
Refinement method	Full-matrix least-squares on F^2	
Data / restraints / parameters	5884 / 126 / 512	
Goodness-of-fit on F^2	1.065	
Final R indices [I>2sigma(I)]	R_1 = 0.0678, wR_2 = 0.1607	
R indices (all data)	R_1 = 0.1051, wR_2 = 0.1788	
Largest diff. peak and hole	0.700 and -0.503 e.$Å^{-3}$	

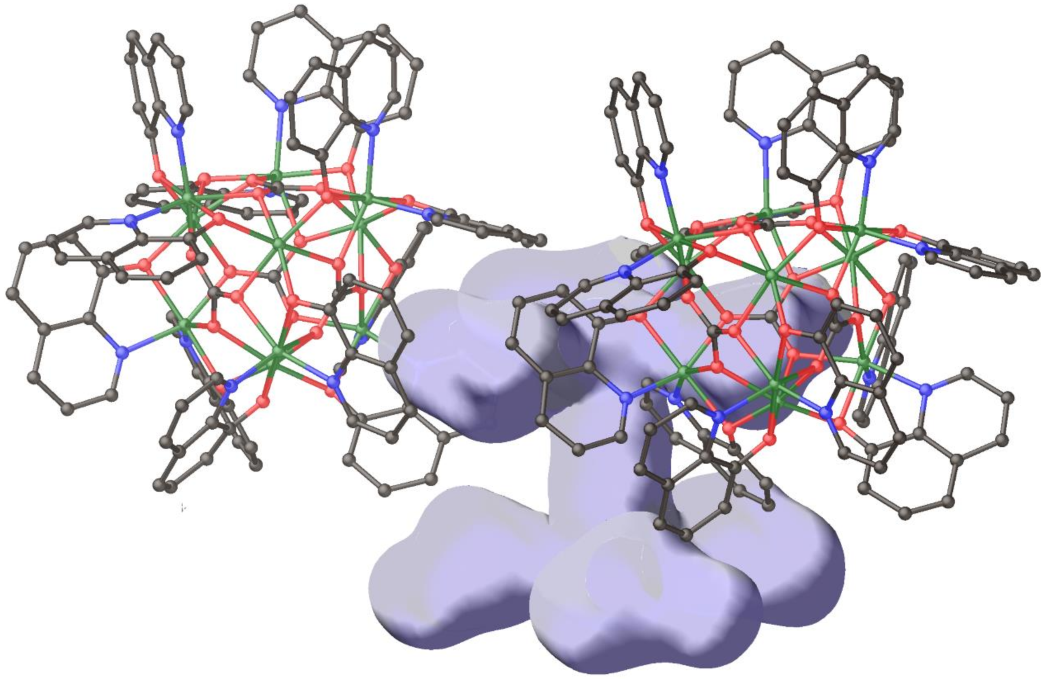

Figure S3. Void space of **WUT-2(Ni)** based on single crystal data where blue represent isolated pores with a complex shape resembling two centrally fused three-leaf clovers space within structure.

*Analysis of the intermolecular interactions in **WUT-1(Ni)** and **WUT-2(Ni)** frameworks:* The aromatic rings of the three axial quinolinate ligands, which form respective micropockets, collectively create four triangular layouts with a tetrahedral spatial arrangement around the Ni(II) carbamato core, resulting in a specific shape of clusters **1** (Figure S4). These clusters self-assemble in crystals through a 3D network of cooperative intermolecular CH-π interactions, in which we can distinguish two types of Supramolecular synthons formed between neighboring interacting molecules. Supramolecular synthon I is highly symmetric, exhibiting nearly perfect interlocking of clusters through shape complementarity among the three quinolinate layouts of each cluster (Figure S4a). The formation of this synthon is associated with a series of six cooperative CH-π interactions (distances between the center of C_6 ring center and H atom are about 2.79 Å) between hydroxyquinolinate rings, which simultaneously act as both H-donor for one interaction and H-acceptor for the other. In contrast, Supramolecular synthon II exhibits less efficient interlocking, utilizing shape complementarity between only two quinolinate layouts per cluster (Figure S4b). The formation of this synthon involves a series of less specific cooperative CH-π interactions (distances between the center of C_6 ring center and H atom are in the range 2.80-3.10 Å), in which individual hydroxyquinolinate rings can act solely as H-donors, solely as H-acceptors, or as both H-donors and H-acceptors simultaneously. The framework of **WUT-1(Ni)** is constructed from clusters of **1** connected exclusively through Supramolecular Synthon I, forming an ideal diamondoid network of interactions (Figure S5a). In contrast, the framework of **WUT-2(Ni)** features a more complex topology, predominantly governed by Supramolecular synthon II, with only a few connections via Supramolecular synthon I (Figure S5b).

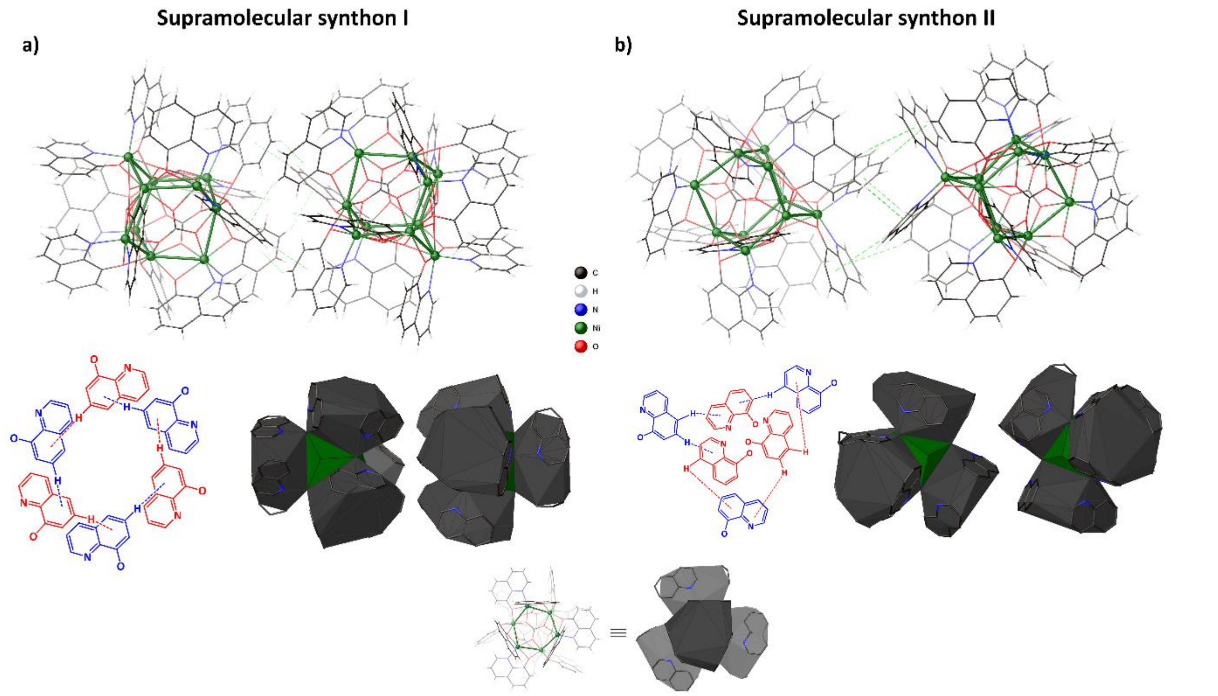

Figure S4. Structural and schematic representations of the supramolecular synthons found in **WUT-1(Ni)** and **WUT-2(Ni)** frameworks**.**

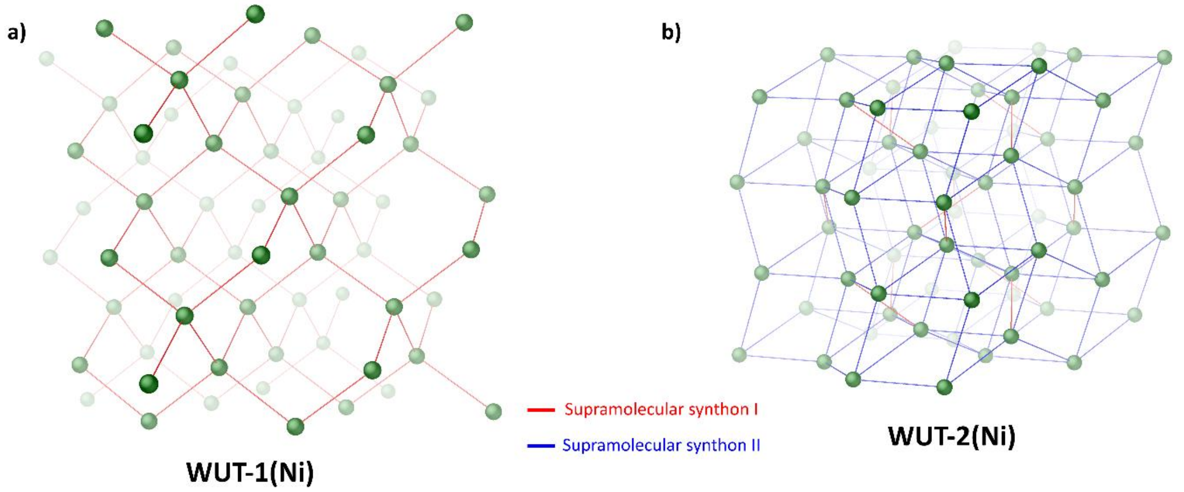

Figure S5. Topological representation of the intermolecular interactions in **WUT-1(Ni)** and **WUT-2(Ni)** frameworks.

4. Powder X-Ray Diffraction Analysis

Powder X-ray diffraction (PXRD) data were collected on Empyrean diffractometer (PANalytical). Measurements employed Ni-filtered Cu Kα radiation of a copper sealed tube charged with 40 kV voltage and 40 mA current and Bragg Brentano geometry and a Si zero-background holder. Diffraction patterns were measured in the range of 2θ = 3–50° of scattering angle by step scanning with step of 0.02°.

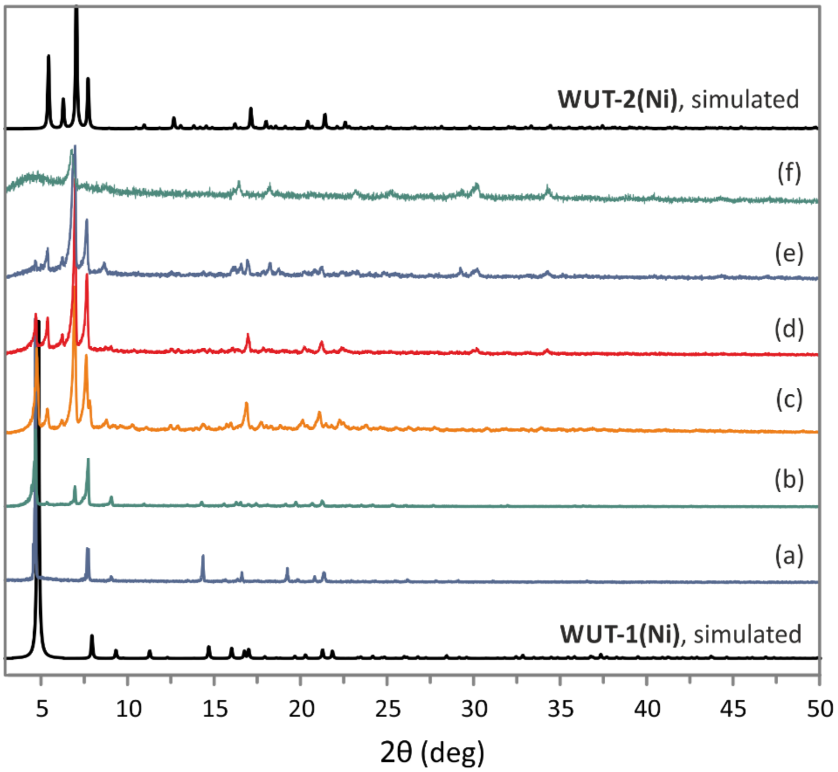

Figure S6. PXRD patterns of product of reaction system $Ni(CH_3COO)_2 \cdot 4H_2O$/L-H/NaOH/$CO_2$ in molecular ratio: (a) 10:12:1,5; (b) 10:12:3; (c) 10:12:5; (d) 10:12:10; (e) 10:12:15; (f) 10:12:20 with reference pattern of **WUT-1(Ni)** and **WUT-2(Ni)** generated from the .cif file (black lines).

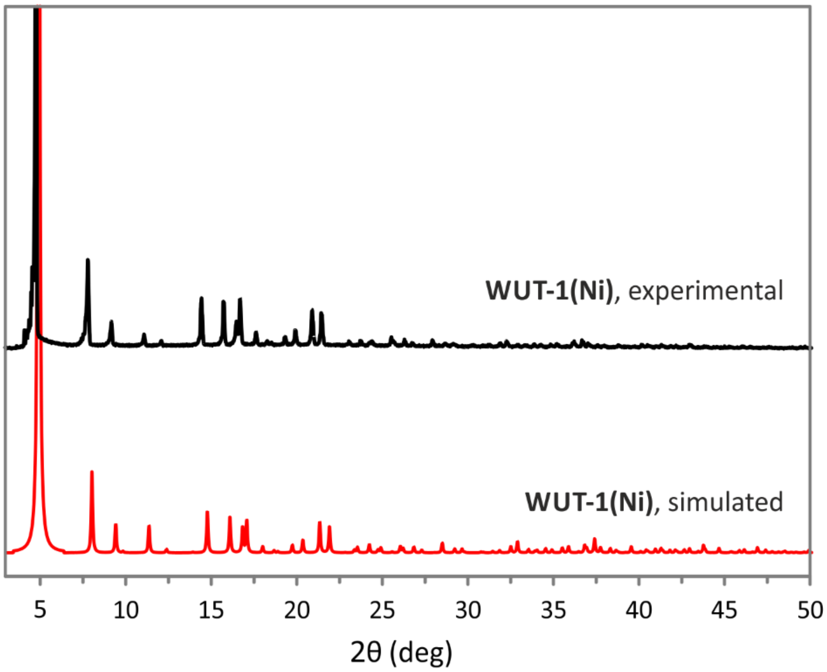

Figure S7. PXRD pattern of a bulk sample of as-synthesized **WUT-1(Ni)** (black line) in reaction system $Ni(CH_3COO)_2{\cdot}4H_2O$/L-H/($N(CH_3)_4OH$)/$CO_2$ and the reference PXRD pattern of **WUT-1(Ni)** generated from the .cif file (red line).

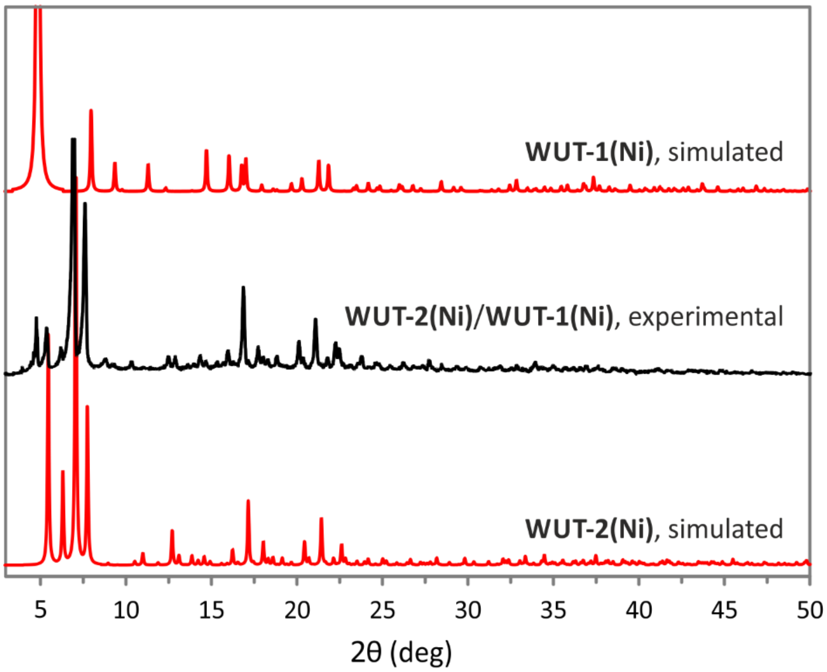

Figure S8. PXRD pattern of a bulk sample of a **WUT-2(Ni)/WUT-1(Ni)** mixture after direct synthesis (black line) and reference PXRD patterns of **WUT-1(Ni)** and **WUT-2(Ni)**, generated from the .cif file (red line).

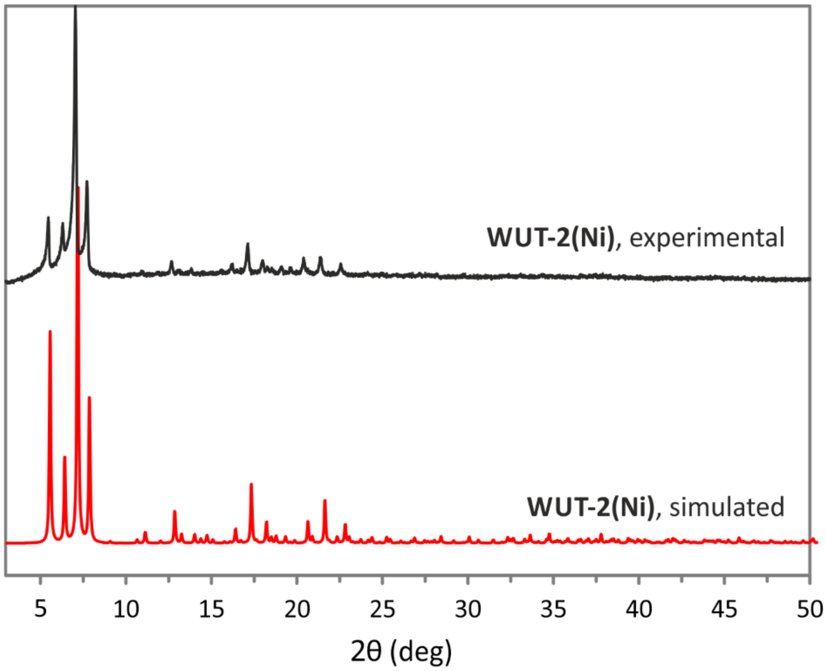

Figure S9. PXRD pattern of a bulk sample of **WUT-2(Ni)** (black line) obtain via transformation of **WUT-1(Ni)** at 270°C and reference PXRD pattern of **WUT-2(Ni)**, generated from the .cif file (red line).

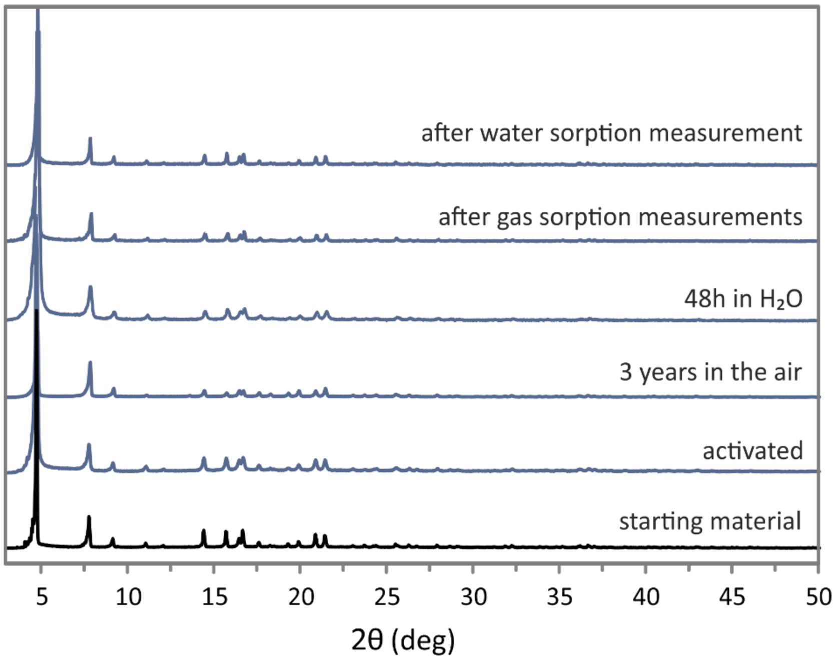

Figure S10. PXRD patterns of **WUT-1(Ni)**, after different treatments in comparison to the starting material sample pattern.

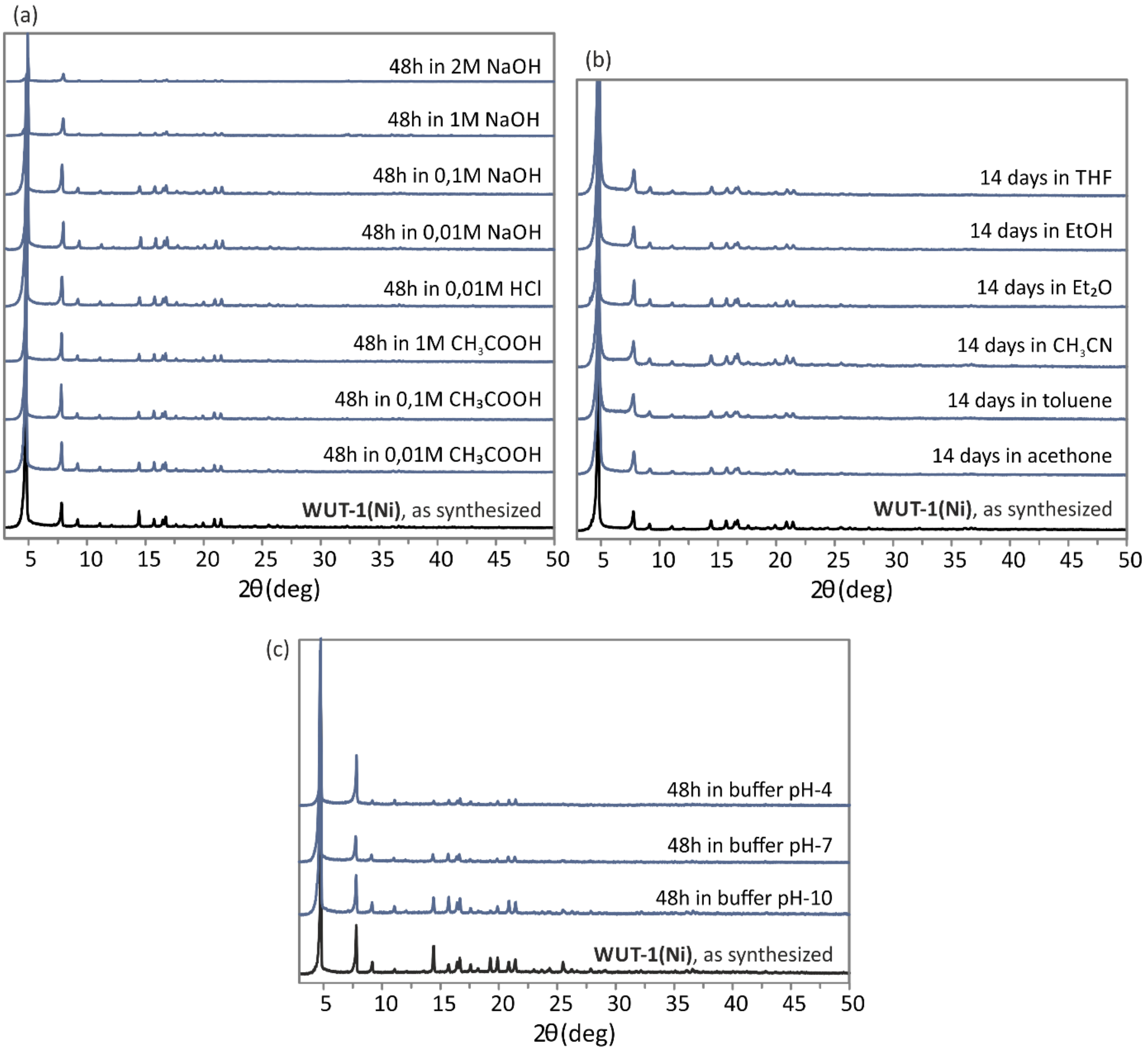

Figure S11. PXRD patterns of **WUT-1(Ni)**; (a) after immersion for 48h in CH_3COOH, HCl, and NaOH of varying concentration, (b) after immersion for 14 days in various solvents, (c) after immersion for 48h in buffers pH = 4, pH = 7, pH = 10 in comparison to the as-synthesized sample pattern.

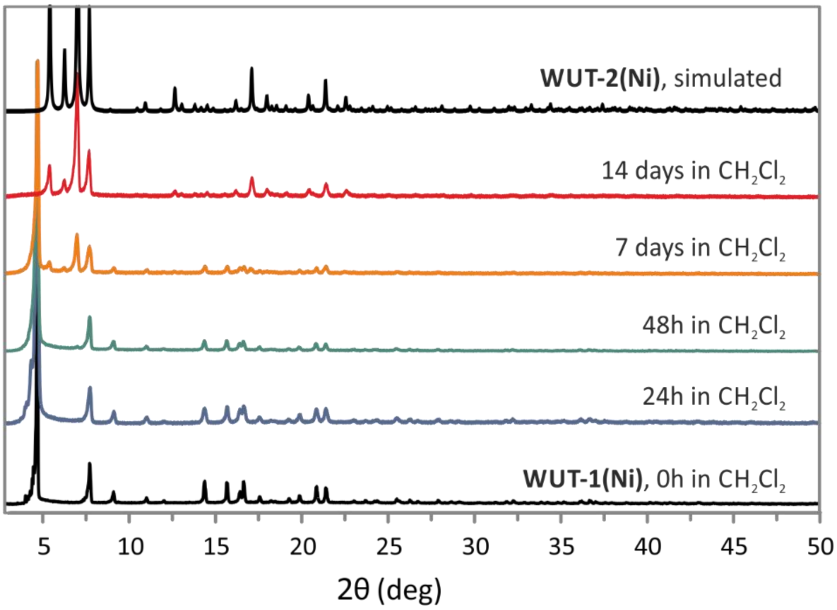

Figure S12. PXRD patterns of phase transformation between **WUT-1(Ni)** and **WUT-2(Ni)** upon immersion in CH_2Cl_2.

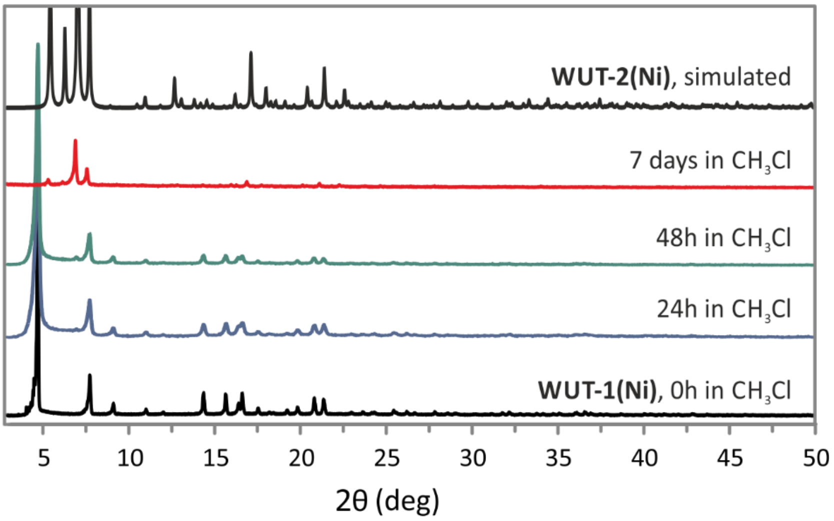

Figure S13. PXRD patterns of phase transformation between **WUT-1(Ni)** and **WUT-2(Ni)** upon immersion in CH_3Cl.

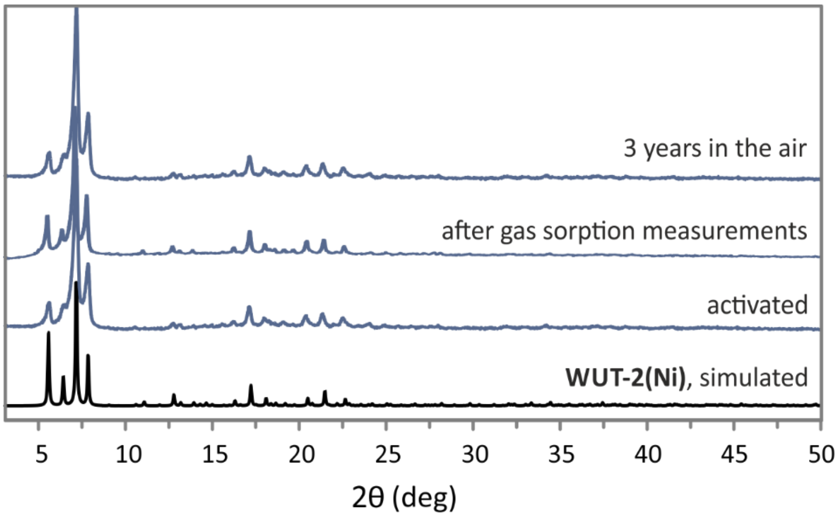

Figure S14. PXRD patterns of **WUT-2'(Ni)**, after different treatments with a reference pattern of **WUT-2(Ni)**, generated from the .cif file.

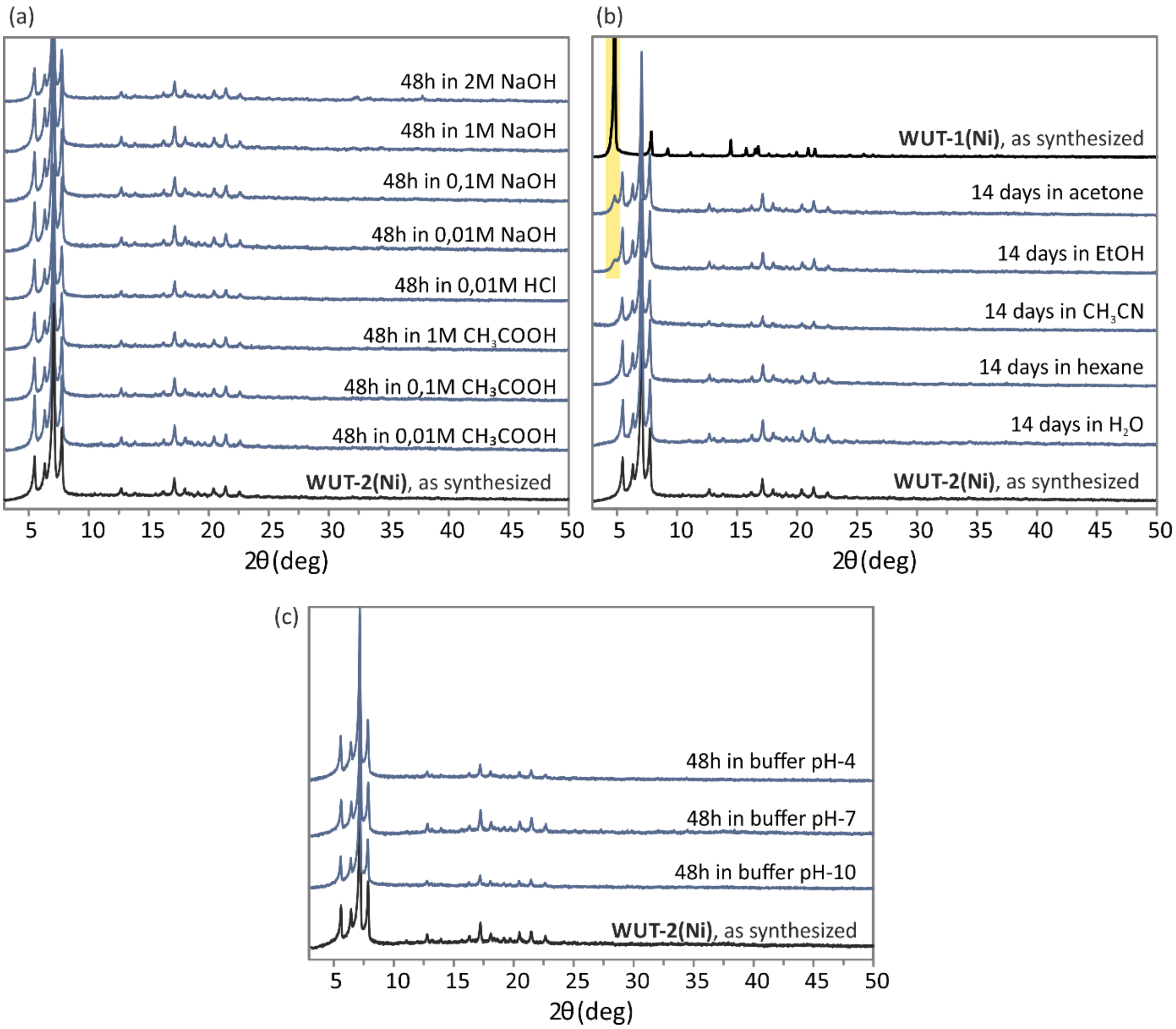

Figure S15. PXRD patterns of **WUT-2(Ni)**; (a) after immersion for 48h in CH_3COOH, HCl and NaOH of varying concentration, (b) after immersion for 14 days in various solvents, (c) after immersion for 48h in buffers pH = 4, pH = 7, pH = 10 in comparison to the as-synthesized **WUT-2(Ni)** and **WUT-1(Ni)** samples pattern.

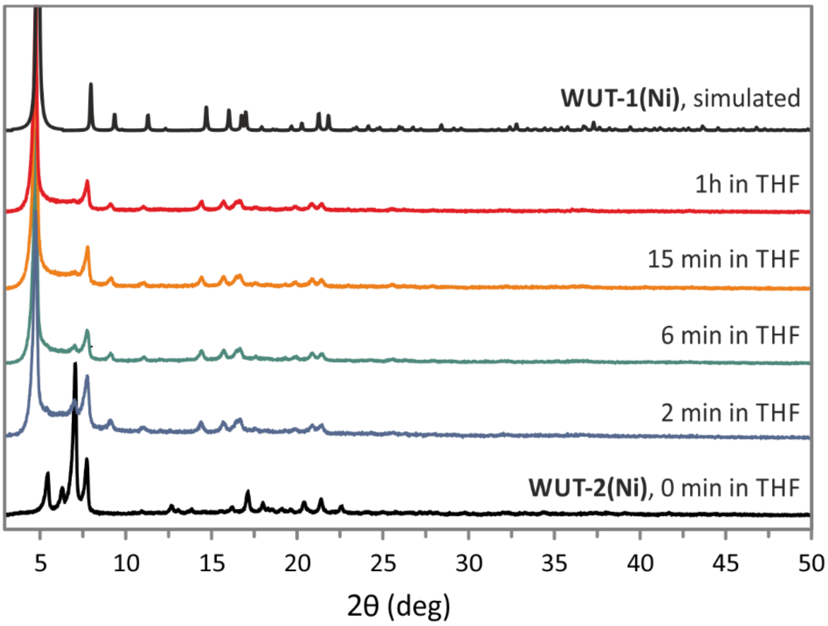

Figure S16. PXRD patterns of phase transformation between **WUT-2(Ni)** and **WUT-1(Ni)** upon immersion in THF.

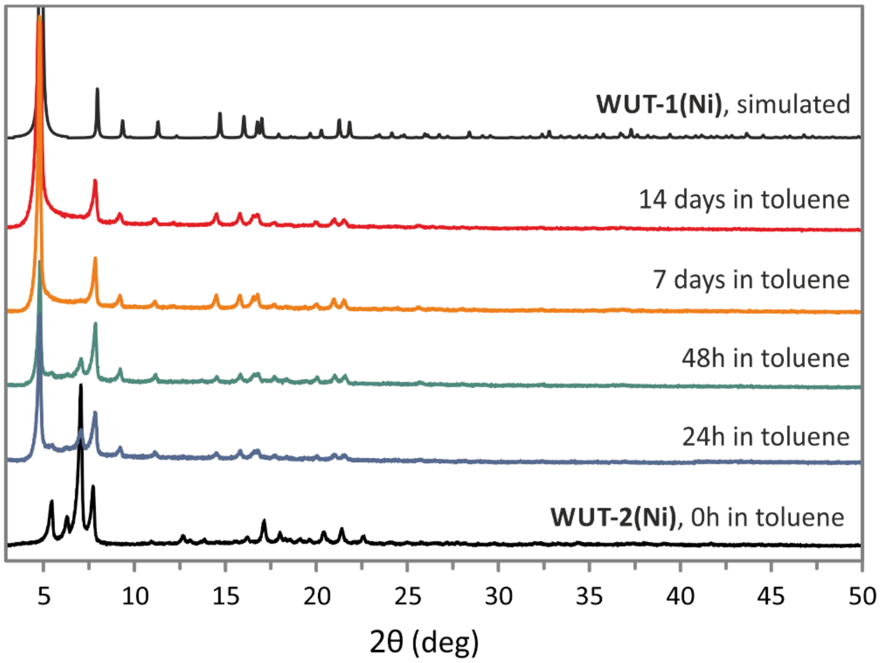

Figure S17. PXRD patterns of phase transformation between **WUT-2'(Ni)** and **WUT-1(Ni)** upon immersion in toluene.

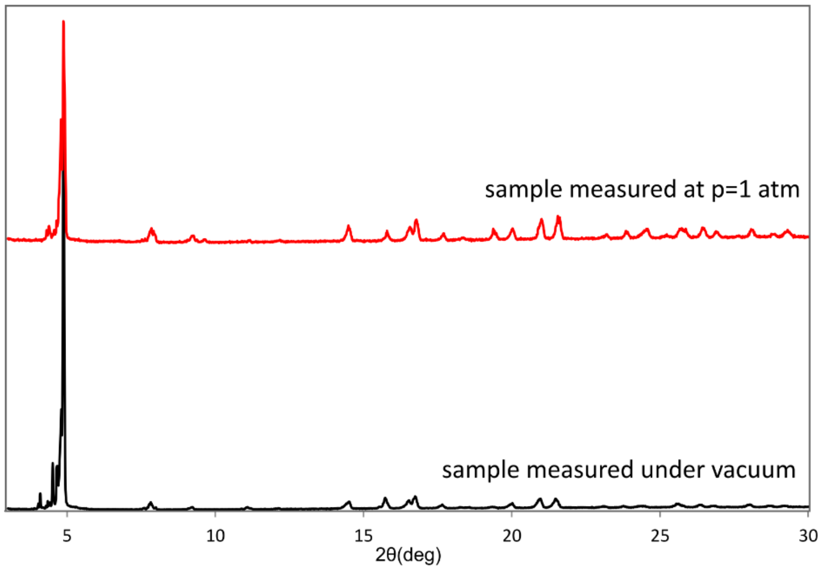

Figure S18. PXRD patterns of **WUT-1'(Ni)** sample measured at 194 K under 1 atm of CO_2 and under vacuum.

5. Variable-temperature Powder X-ray Diffraction Analysis

Variable-temperature X-ray powder diffraction patterns were recorded with Empyrean diffractometer (PANalytical) equipped with a copper lamp (40 kV, 40 mA). Samples were mounted inside the Anton Paar TTK-450 sample chamber for nonambient X-ray diffraction experiments. For the measurements, Ni-filtered Cu-Kα (λ = 0.154 nm) radiation was used and was detected with a X'Celerator 1D detector in Bragg-Brentano θ-θ geometry. The XRD patterns were recorded over a 2θ range of 3° to 50° with a step size of 0.0084. The samples were not rotated. The samples were heated at 20°C/min rate and thermostated for 30 min prior to each measurement.

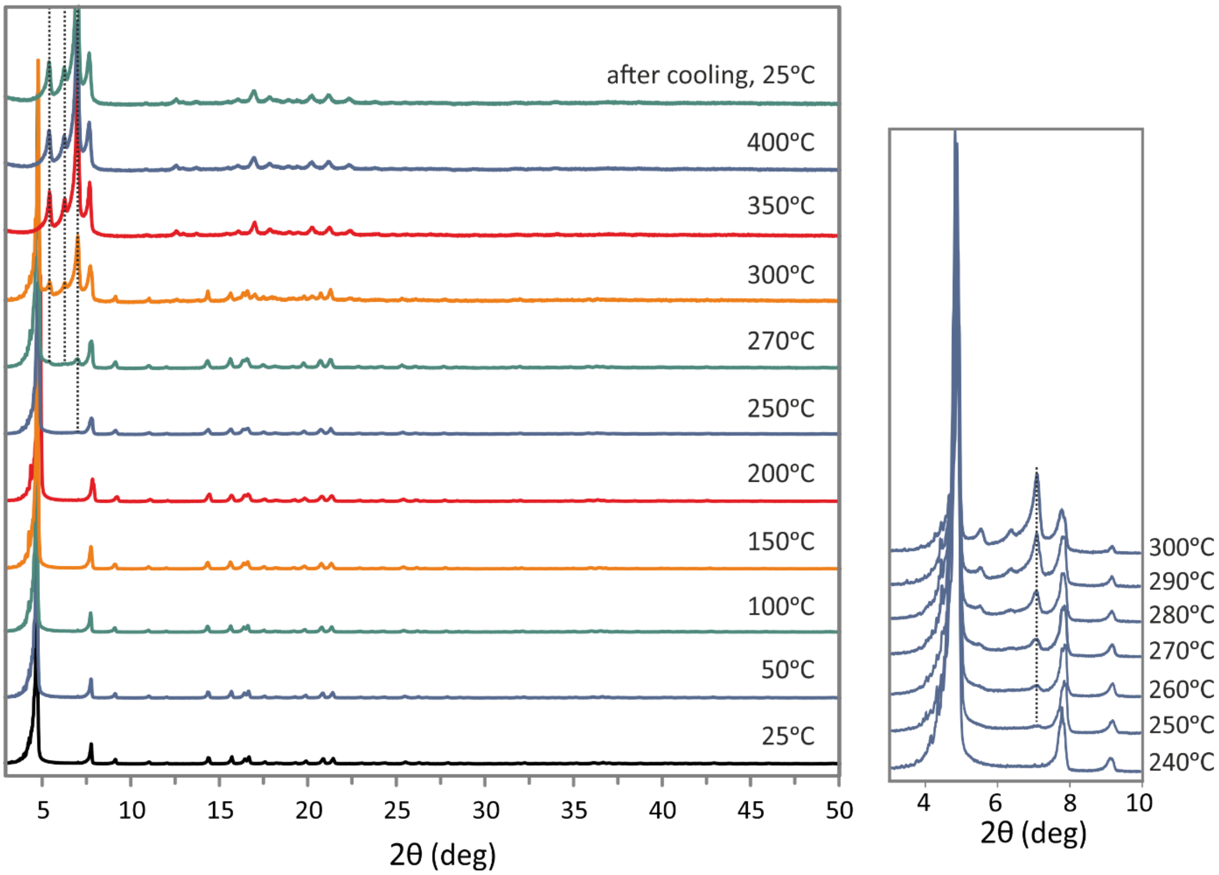

Figure S19. VT PXRD profiles of the microcrystalline sample of **WUT-1(Ni)** in vacuum. The dotted line highlight the apparition of new reflections indicative of the formation of a dense crystal phase **WUT-2(Ni).**

6. Thermal Analysis

Thermogravimetric Analysis (TGA)

Thermogravimetric and derivative thermogravimetric analyses (TGA/DTG) were recorded on TA Instruments Q600 apparatus. Samples for thermogravimetric characterization were placed in alumina crucibles in argon atmosphere (flow rate: 100ml/min) at heating rate of 5°C/min. All samples were studied in the temperature range of 30 - 800 °C.

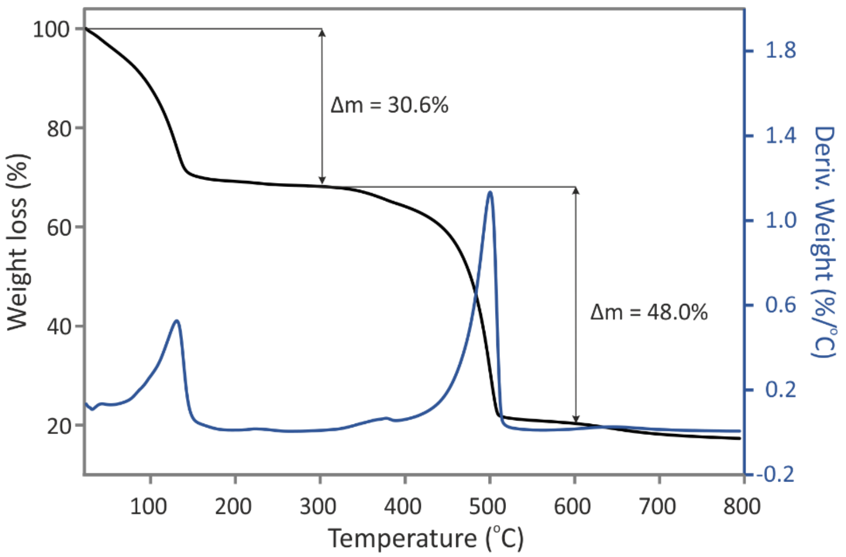

Figure S20. TGA and DTG profiles of the as-synthesized sample of **WUT-1(Ni)** under argon atmosphere.

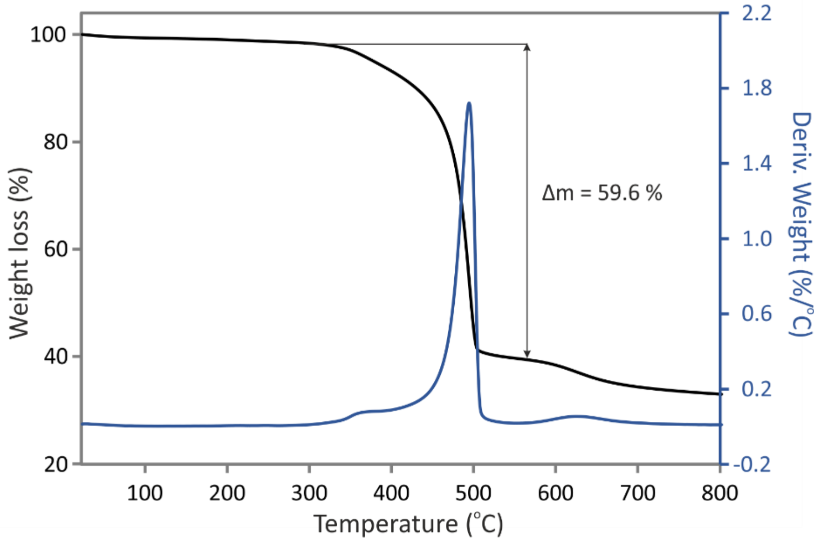

Figure S21. TGA and DTG profiles of the activated sample of **WUT-1'(Ni)** under argon atmosphere.

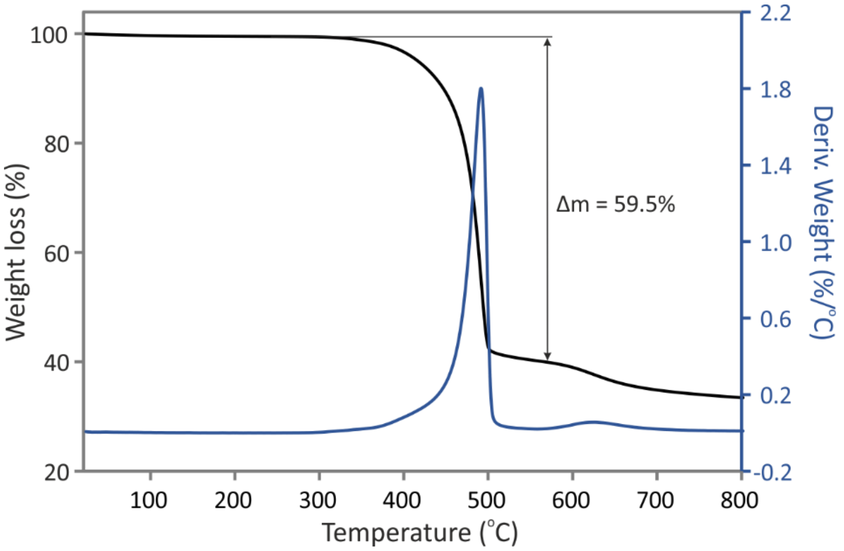

Figure S22. TGA and DTG profiles of the activated sample of **WUT-2’(Ni)** under argon atmosphere.

7. Differential scanning calorimetry of WUT-1’(Ni)

Differential Scanning Calorimetry (DSC) heating and cooling traces were obtained using a Mettler-Toledo DSC 3 instrument. The measurements were performed between 100 and 310°C with the ramp rate of 50 deg/min. .

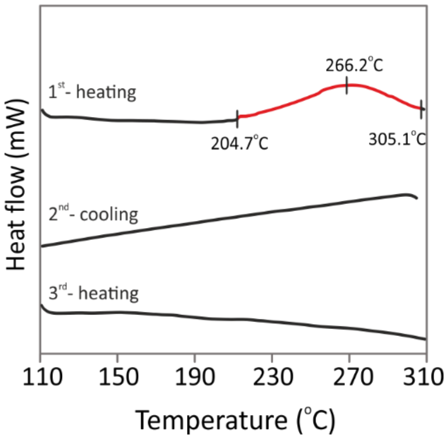

Figure S23. DSC traces, two heating and one cooling for **WUT-1’(Ni)** (sample mass, m = 0.70 mg). The broad peak in the range 204.7 – 305.1°C corresponds to a crystal phase transition.

8. Optical Microscope images

The optical microscope images were collected using the Olympus BX53 microscope.

(a)

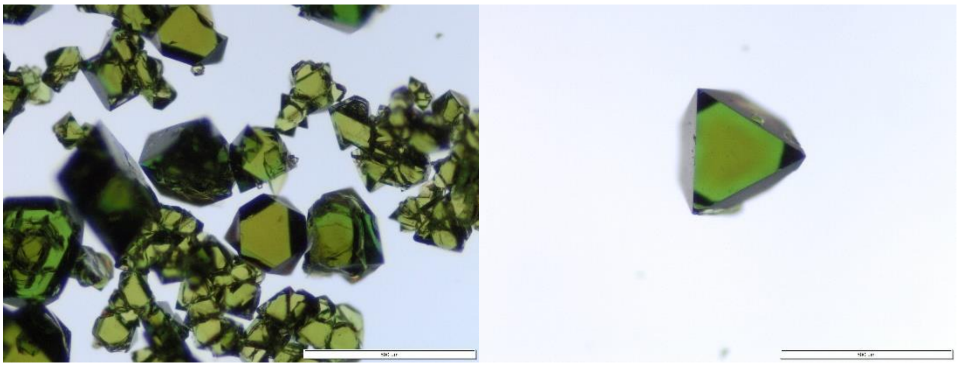

(b)

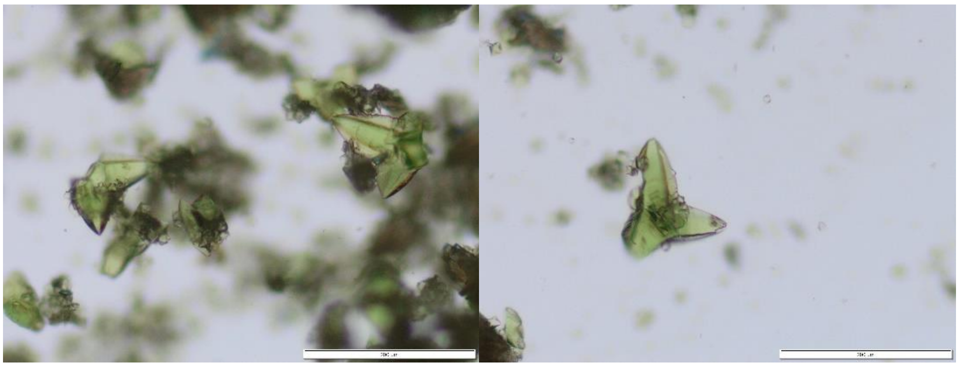

(c)

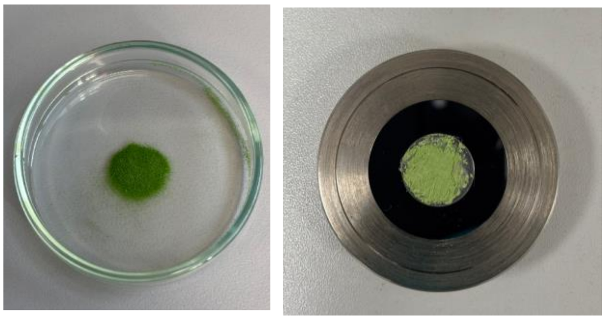

(d)

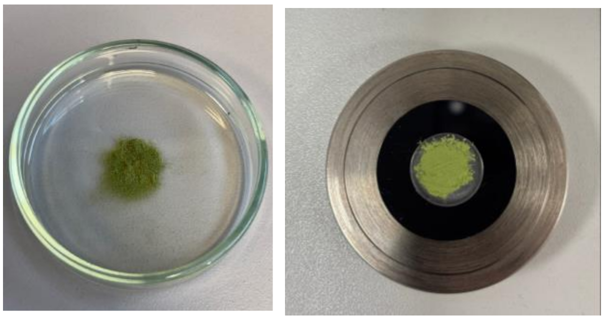

Figure S24. Optical microscope recording the crystals of: (a) **WUT-1(Ni)** and (b) **WUT-2(Ni)** selected from a sample containing a mixture of **WUT-1(Ni)** and **WUT-2(Ni)**. Photography of primitive and grinded samples of: (c) **WUT-1(Ni)**, (d) **WUT-2(Ni)** obtained by thermal transition from **WUT-1(Ni)**.

9. Scanning Electron Microscopy

SEM imaging was performed using FEI Nova NanoSEM 450 system equipped with a field emission electron gun operating at 1 and 2 kV.

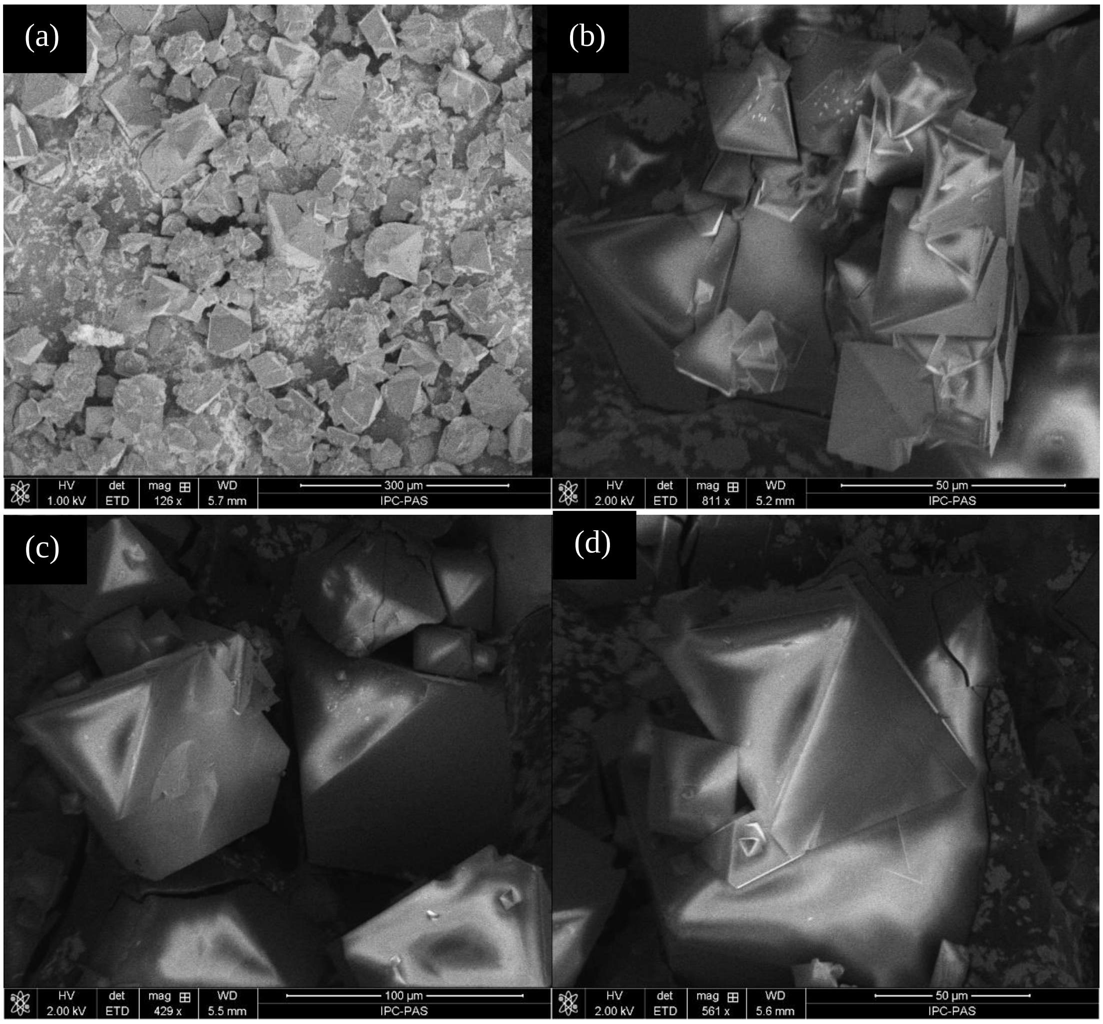

Figure S25. (a)-(d) SEM images of the microcrystalline powder of **WUT-1(Ni)**.

10. Gas adsorption experiments and adsorption simulations

* *Gas adsorption measurements up to 1 bar*

Volumetric gas sorption studies up to 1 atm were undertaken using an ASAP 2020 system, Micromeritics Instrument Corporation (Norcross, Georgia, USA).

Approximately 150 mg of microporous materials were transferred to a sample tube and evacuated under vacuum at 25°C on the gas adsorption apparatus until the outgas rate was <5 µmHg to give fully activated material. Used gases: N_2, H_2, CO_2, CH_4 were of 99.999% purity. Helium was used for the free-space determination after sorption analysis. Adsorption isotherms at 77 K were measured using a liquid nitrogen bath, and at 87 K using a liquid argon bath. The temperatures of 273 K, 293 K and 298 K for CO_2, CH_4, N_2, H_2 measurements were maintained by chilled water/ethylene glycol circulating bath. Adsorption isotherm in 195 K was measured using ethanol/dry ice cooling bath.

** *Gas adsorption measurements up to 10 bar*

Volumetric gas sorption studies up to 10 atm were undertaken using an ASAP 2050 system, Micromeritics Instrument Corporation (Norcross, Georgia, USA).

In gas adsorption experiment, 100 mg of sample was degassing at 25°C and high vacuum of about 10 µmHg for 12 hours to give fully desolvated material. Used gases: N_2, H_2, CO_2, CH_4 were of 99.999% purity and O_2 was of 99.99% purity. Helium was used for the free-space determination after sorption analysis. Adsorption isotherms at 77 K were measured using a liquid nitrogen bath, measurements at 273 K and 298 K were maintained by chilled water/ethylene glycol circulating bath.

*** *Gas adsorption simulations*

The simulated adsorption isotherms were investigated using grand canonical Monte Carlo (GCMC) simulations performed in the multi-purpose code RASPA.[5] We used an atomistic model of WUT where the framework atoms were kept fixed at their crystallographic positions. We used the standard Lennard-Jones (LJ) 12-6 potential to model the interactions between the framework and fluid atoms. In addition, a Coulomb potential was used for fluid-fluid interactions. The parameters for the framework atoms were derived from a mix of the Dreiding Force Field (DFF)[6] and the Universal Force Field (UFF)[7], whereas all the guest molecules were modelled using the TraPPE potential with charges placed on each atom and at the centre of mass (Supporting Table 1).[8] Quantum diffraction effects were taken into account for H_2 at 77 K and 87 K using Feynman-Hibbs corrections.[9] EQeq was used to assign the partial charges of the framework.[10] The Lorentz-Berthelot mixing rules were employed to calculate fluid-solid LJ parameters, and LJ interactions beyond 12.8 Å were neglected. The Ewald sum method was used to compute the electrostatic interactions. Up to 200,000 Monte Carlo cycles were performed, the first 10% of which were used for equilibration and the remaining steps were used to calculate the ensemble averages. Monte Carlo moves consisted of insertions, deletions, displacements, and rotations. In a cycle, *N* Monte Carlo moves are

attempted, where *N* is defined as the maximum of 20 or the number of adsorbates in the system. To calculate the gas-phase fugacity we used the Peng-Robinson equation of state.[11] The pore size distribution (PSD) was obtained using RASPA with a probe distance of sigma and 10,000 cycles.

Table S5. Forcefield parameters for the mix of UFF and DFF used to model **WUT-1(Ni)** and **WUT-2(Ni)**, and TraPPE parameters used to model the gases considered here. The 3rd parameter used for the Feynman-Hibbs corrected LJ parameters in the case of hydrogen is 1.00782505.

	**DFFUFF**	
	**σ (Å)**	**ε (K)**
**C**	3.47299	47.8562
**H (MOF)**	2.84642	7.64893
**N**	3.26256	38.9492
**O**	3.03315	48.1581
**Ni**	2.52481	7.54829
	**TraPPE**	
**N_N_2**	3.310	36.00
**H_com**	2.958	36.70
**C_CO_2**	2.800	27.00
**O_CO_2**	3.050	79.00
**CH_4**	3.720	158.5
**CH_3_sp3**	3.760	108.0
**CH_2_sp3**	3.960	56.00
**CH_2_sp2**	3.675	85.00
**CH_sp3**	4.670	17.00
**CH_sp2**	3.730	47.00

Gas sorption measurements for WUT-1'(Ni) up to 1 bar

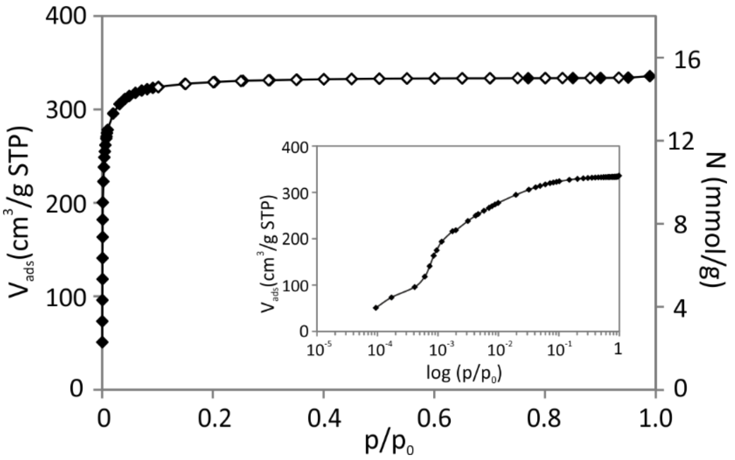

Figure S26. Experimentally measured N_2 uptakes for **WUT-1'(Ni)** at 77 K. Adsorption (closed symbols) and desorption (open symbols).The inset shows N_2 isotherms of **WUT-1'(Ni)** obtained at 77 K using semi-logarithmic scale.

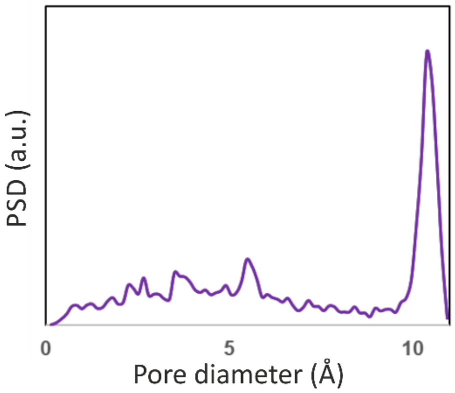

Figure S27. Pore size distribution of **WUT-1'(Ni)** obtained with RASPA.

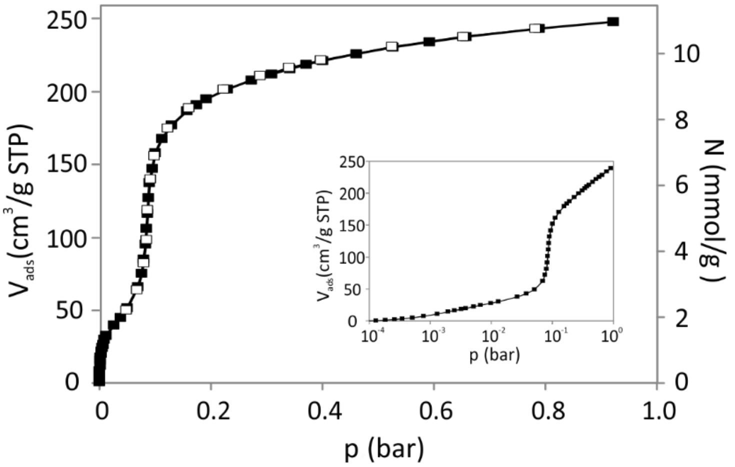

Figure S28. Experimentally measured CO_2 uptakes for **WUT-1'(Ni)** at 195 K up to 1 bar. Adsorption (closed symbols) and desorption (open symbols).The inset shows CO_2 isotherm of **WUT-1'(Ni)** obtained at 195 K using a semi-logarithmic scale.

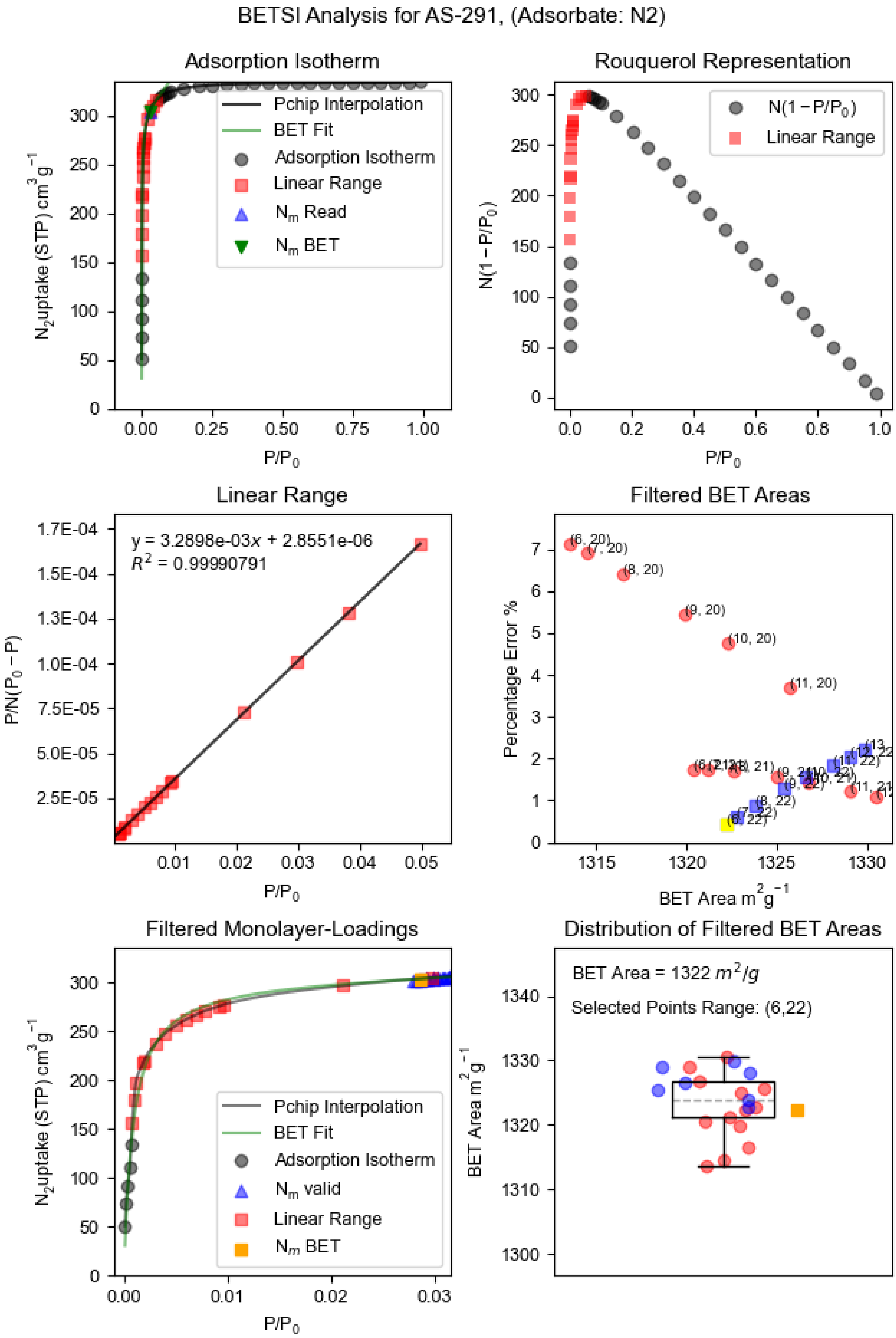

Figure S29. BETSI[12] fitting and BET area calculations for **WUT-1'(Ni)**.

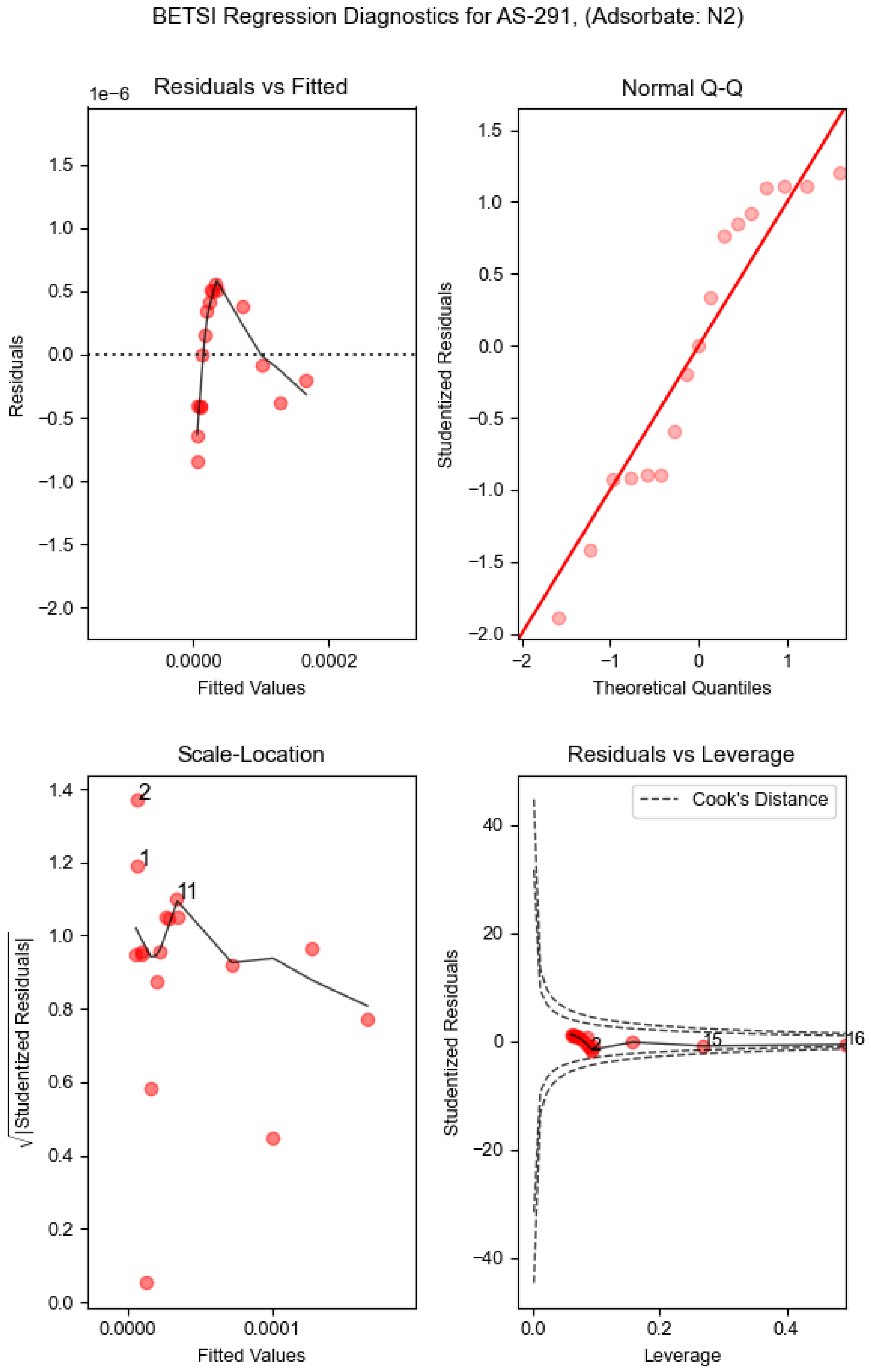

Figure S30. Regression analysis for BETSI fitting and BET area calculations for **WUT-1'(Ni)**.

The experimentally measured values are excess amounts adsorbed (N_{exc}), which are transformed into absolute uptakes (N_{abs}) by using equation: $N_{abs} = N_{exc} + \rho V_{pore}$ where ρ is the density of the gas at the given adsorption pressure and temperature, obtained from the National Institute of Standards and Technology (NIST)[13], and V_{pore} is the pore volume of the adsorbent 20 .[14]

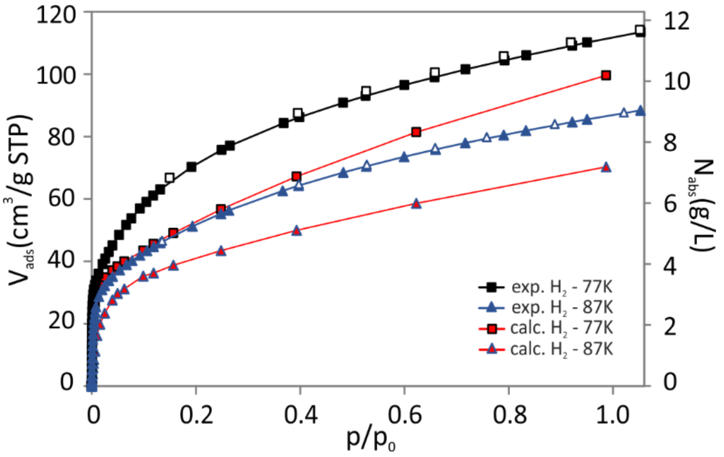

Figure S31. Experimentally measured uptakes (V_{ads}), absolute uptakes (N_{abs}) and simulated (closed red symbols) of H_2 for **WUT-1'(Ni)** at 77 K and 87 K up to 1 bar. Adsorption (closed symbols) and desorption (open symbols).

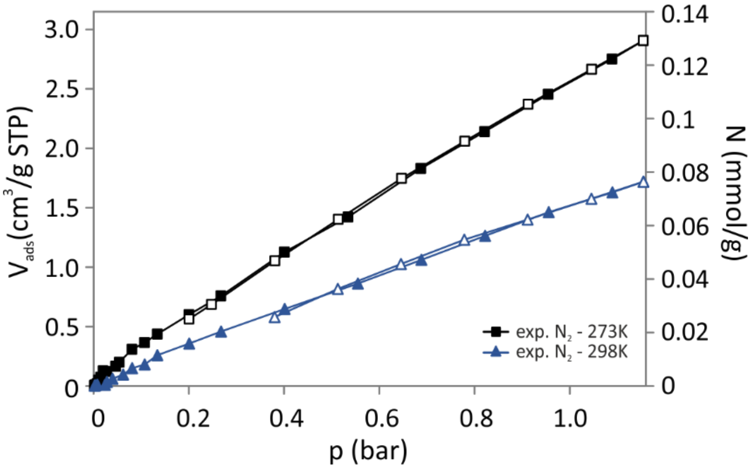

Figure S32. Experimentally measured N_2 uptakes for **WUT-1'(Ni)** at 273 K and 298 K up to 1 bar. Adsorption (closed symbols) and desorption (open symbols).

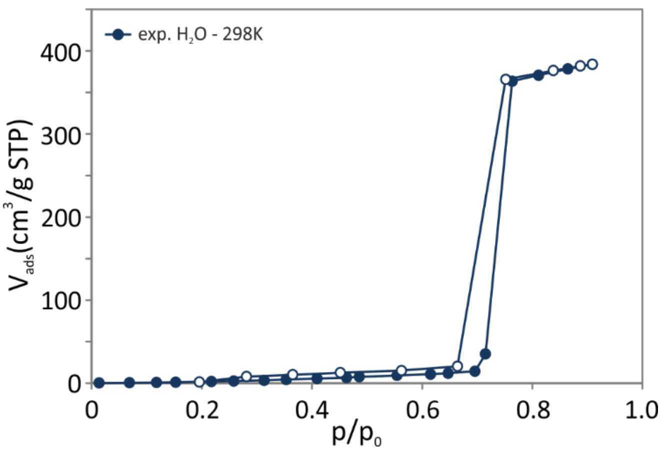

Figure S33. Experimentally measured H_2O vapor uptakes for **WUT-1'(Ni)** at 298 K up to 1 bar. Adsorption (closed symbols) and desorption (open symbols).

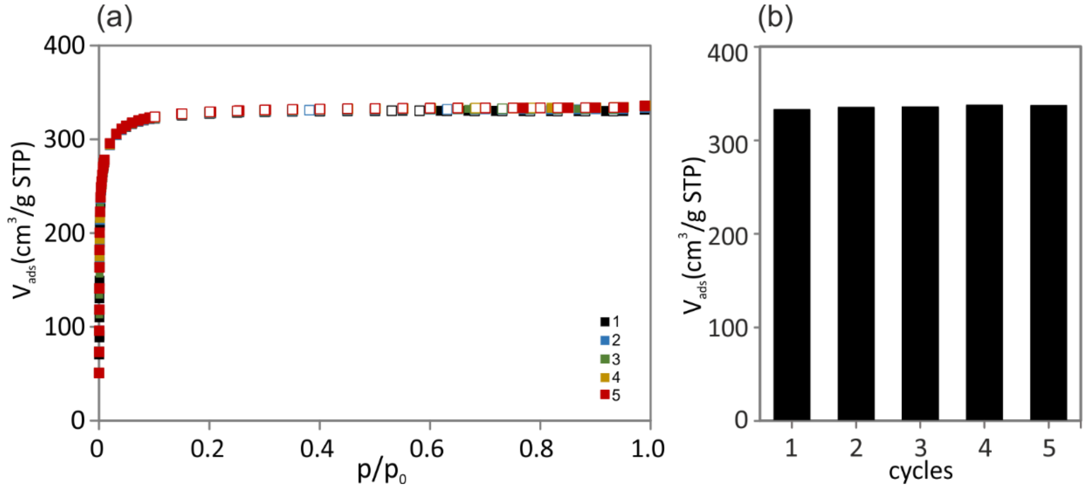

Figure S34. (a),(b) Series of five experimentally measured N_2 uptakes for **WUT-1'(Ni)** at 77 K. Adsorption (closed symbols) and desorption (open symbols).

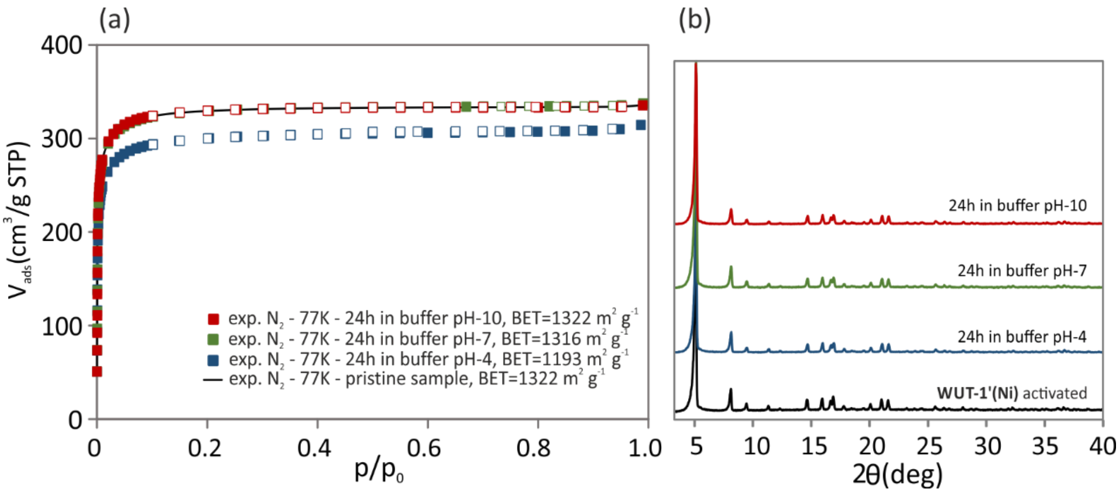

Figure S35. (a) Experimentally measured N_2 adsorption isotherms and (b) PXRD patterns of activated **WUT-1'(Ni)** at 77 K before and after immersion in buffers pH4, pH7 and pH10 for 24 h. Adsorption (closed symbols) and desorption (open symbols).

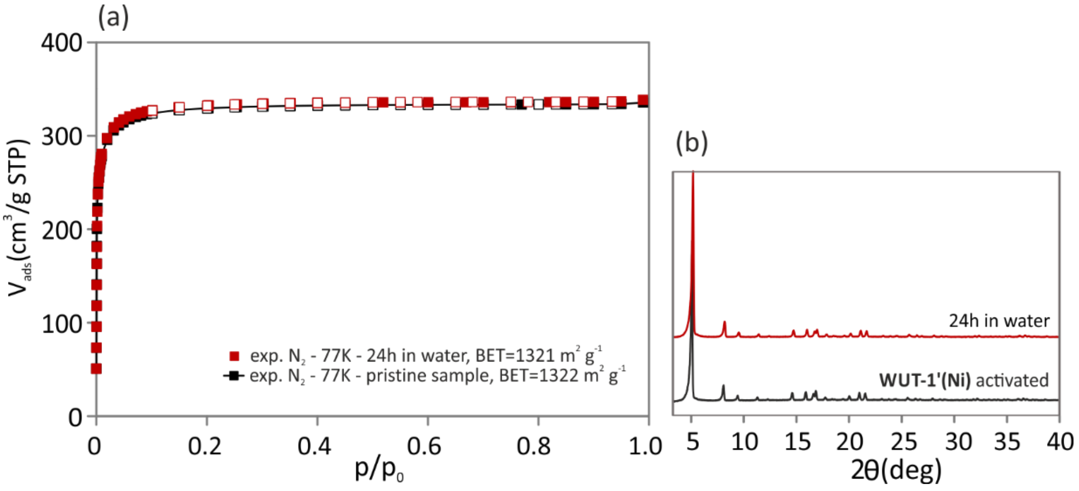

Figure S36. (a) Experimentally measured N_2 adsorption isotherms and (b) PXRD pattern of activated **WUT-1'(Ni)** at 77 K before and after immersion in water for 24 h. Adsorption (closed symbols) and desorption (open symbols).

Gas sorption measurements for WUT-1'(Ni) up to 10 bar

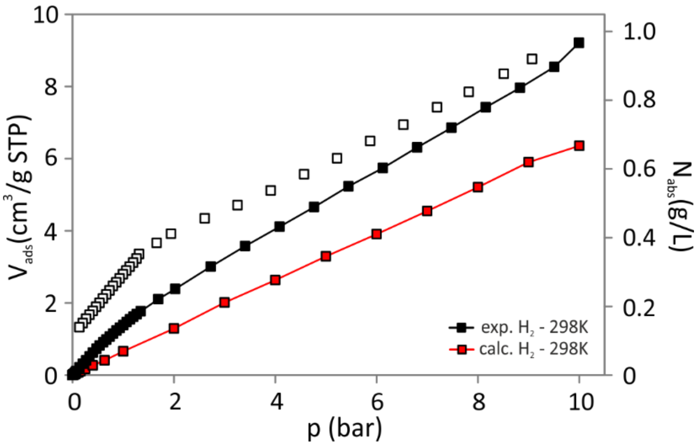

Figure S37. Experimentally measured uptakes (V_{ads}), absolute uptakes (N_{abs}) and simulated (closed red symbols) of H_2 for **WUT-1'(Ni)** at 298 K up to 10 bar. Adsorption (closed symbols) and desorption (open symbols).

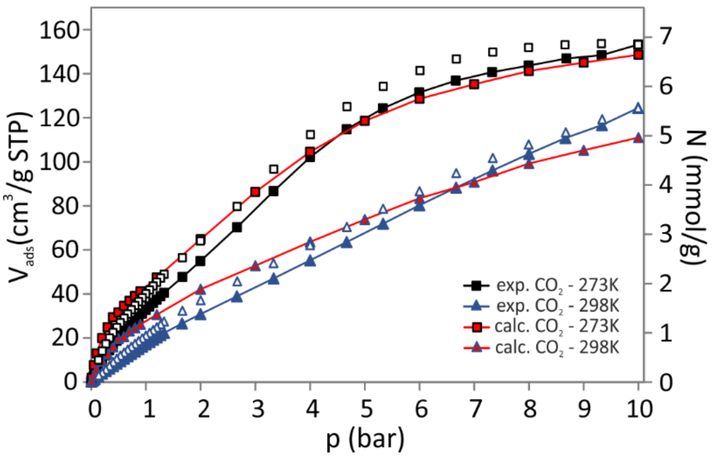

Figure S38. Experimentally measured uptakes and simulated (closed red symbols) of CO_2 for **WUT-1'(Ni)** at 273 K and 298 K up to 10 bar. Adsorption (closed symbols) and desorption (open symbols).

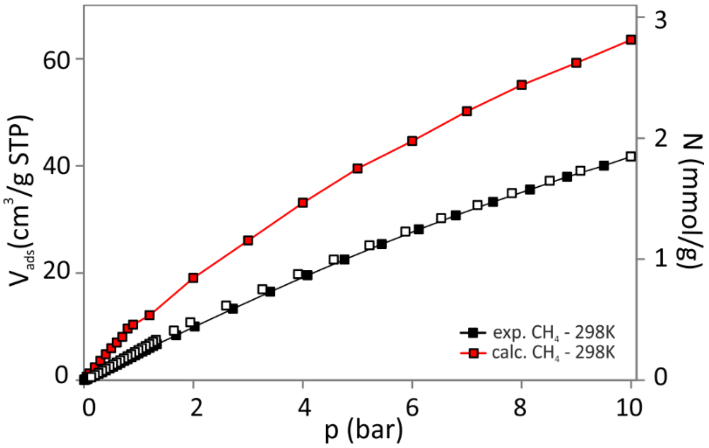

Figure S39. Experimentally measured uptakes and simulated (closed red symbols) of CH_4 for **WUT-1'(Ni)** at 298 K up to 10 bar. Adsorption (closed symbols) and desorption (open symbols).

Gas sorption measurements for WUT-2'(Ni)

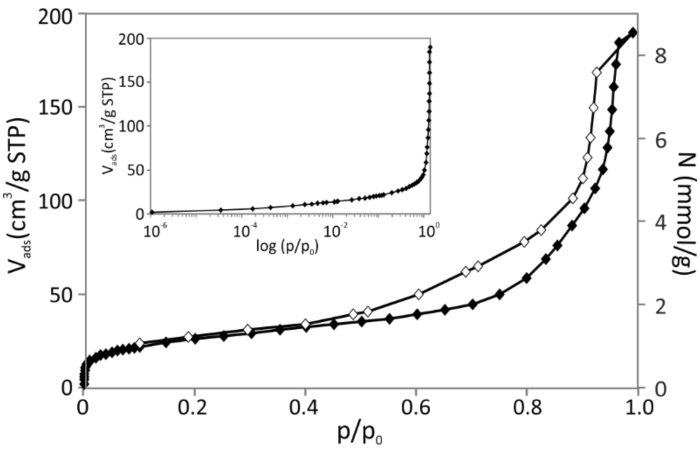

Figure S40. Experimentally measured N_2 uptakes for **WUT-2'(Ni)** at 77 K. Adsorption (closed symbols) and desorption (open symbols).The inset shows N_2 isotherms of **WUT-2'(Ni)** obtained at 77 K using semi-logarithmic scale.

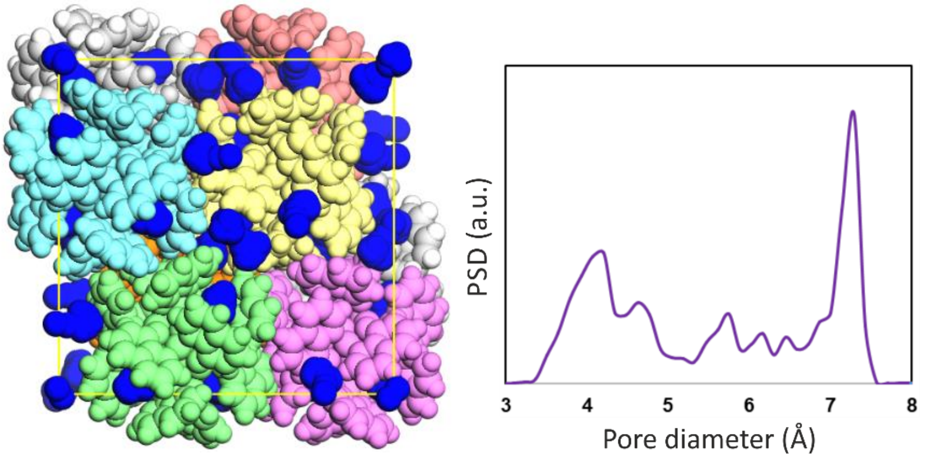

Figure S41. Snapshot of a unit cell of **WUT-2-(Ni)** with N_2 molecules (blue spheres) and pore size distribution of **WUT-2'(Ni)** obtained with RASPA.

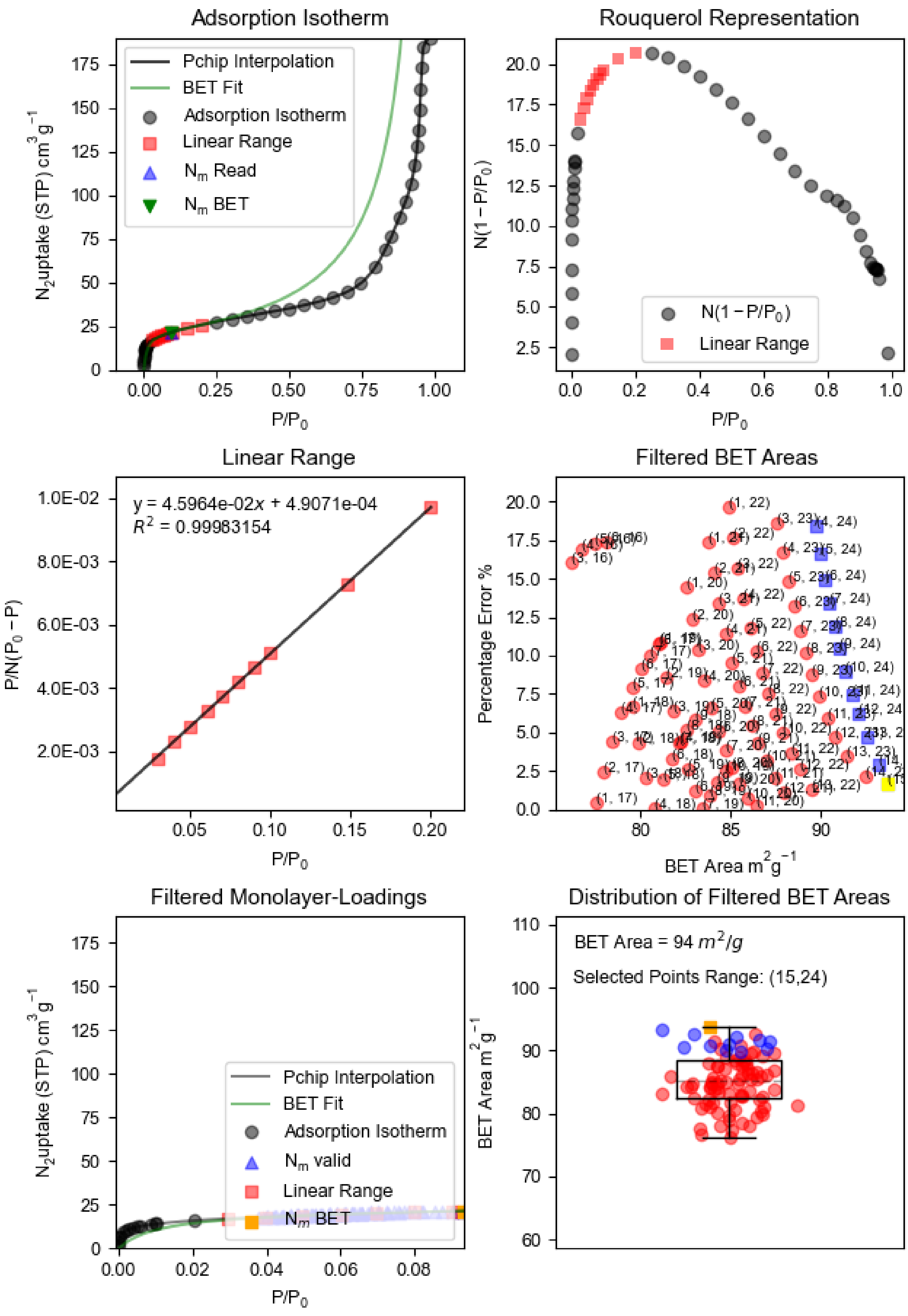

Figure S42. BETSI[12] fitting and BET area calculations for **WUT-2’(Ni)**.

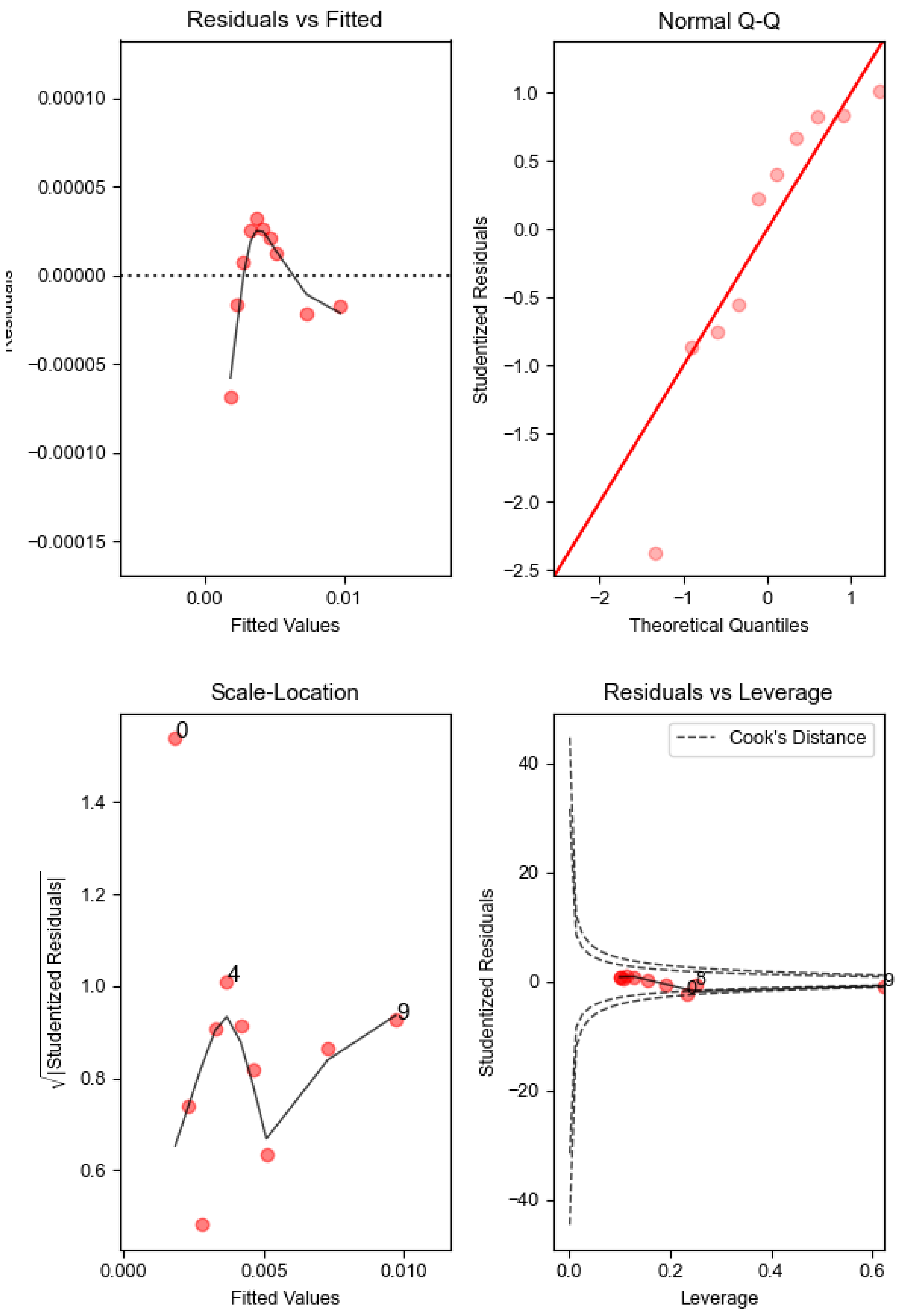

Figure S43. Regression analysis for BETSI fitting and BET area calculations for **WUT-2’(Ni)**.

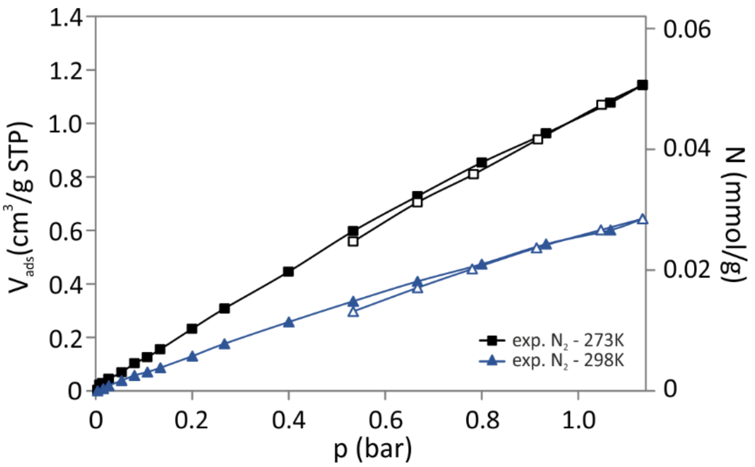

Figure S44. Experimental N_2 uptakes for **WUT-2'(Ni)** at 273 K and 298 K up to 1 bar. Adsorption (closed symbols) and desorption (open symbols).

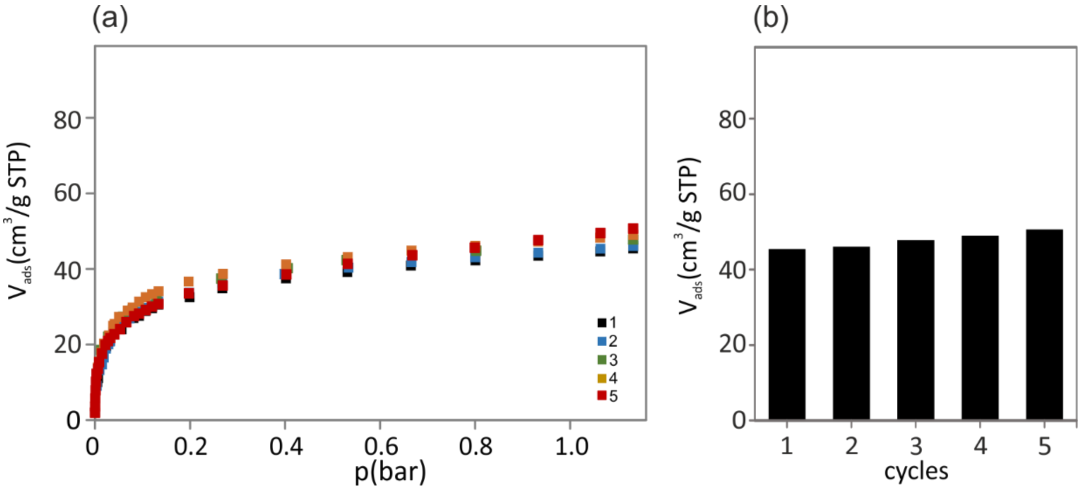

Figure S45. (a),(b) Series of five experimentally measured H_2 uptakes for **WUT-2'(Ni)** at 77 K. Adsorption (closed symbols) and desorption (open symbols).

Comparison of experimental pore volume calculation

The value of BET surface area and experimental pore volume was obtained from the N_2 sorption isotherms (77 K).

Table S6. N_2 sorption data and pore volumes for **WUT-1'(Ni)** and **WUT-2'(Ni).**

Sample	N_2 uptake [cm^3 g^{-1}]	BET [m^2 g^{-1}]	$V_{p(experiment)}$ [cm^3 g^{-1}]	Density [g cm^{-3}]
**WUT-1'(Ni)**	312	1228	0.48	1.17
**WUT-2'(Ni)**	190	94	0.29	1.59

11. The isosteric heats of adsorption

Isosteric heat of adsorption (Q_{st}) values were calculated from experimental isotherms for H_2, CO_2, CH_4 and N_2. Isotherms were fit to the virial equation (1), where n is the amount of gas adsorbed at the pressure p, T is the temperature in K, a_i and b_j are virial coefficients, *m* and *n* are the number of coefficients require to adequately describe the isotherm.

$$\ln \mathrm{p} = \ln \mathrm{n} + \frac{1}{T}\sum_{i=0}^{m} a_i n^i + \sum_{j=0}^{n} b_j\, n^j \qquad (1)$$

To calculate isosteric heat of adsorption (Q_{st}), the fitting parameters a_i from the above equation were input in to equation (2). R is the universal gas constant.

$$\mathrm{Q}_{st} = -\mathrm{R}\sum_{i=0}^{m} a_i n^i \qquad (2)$$

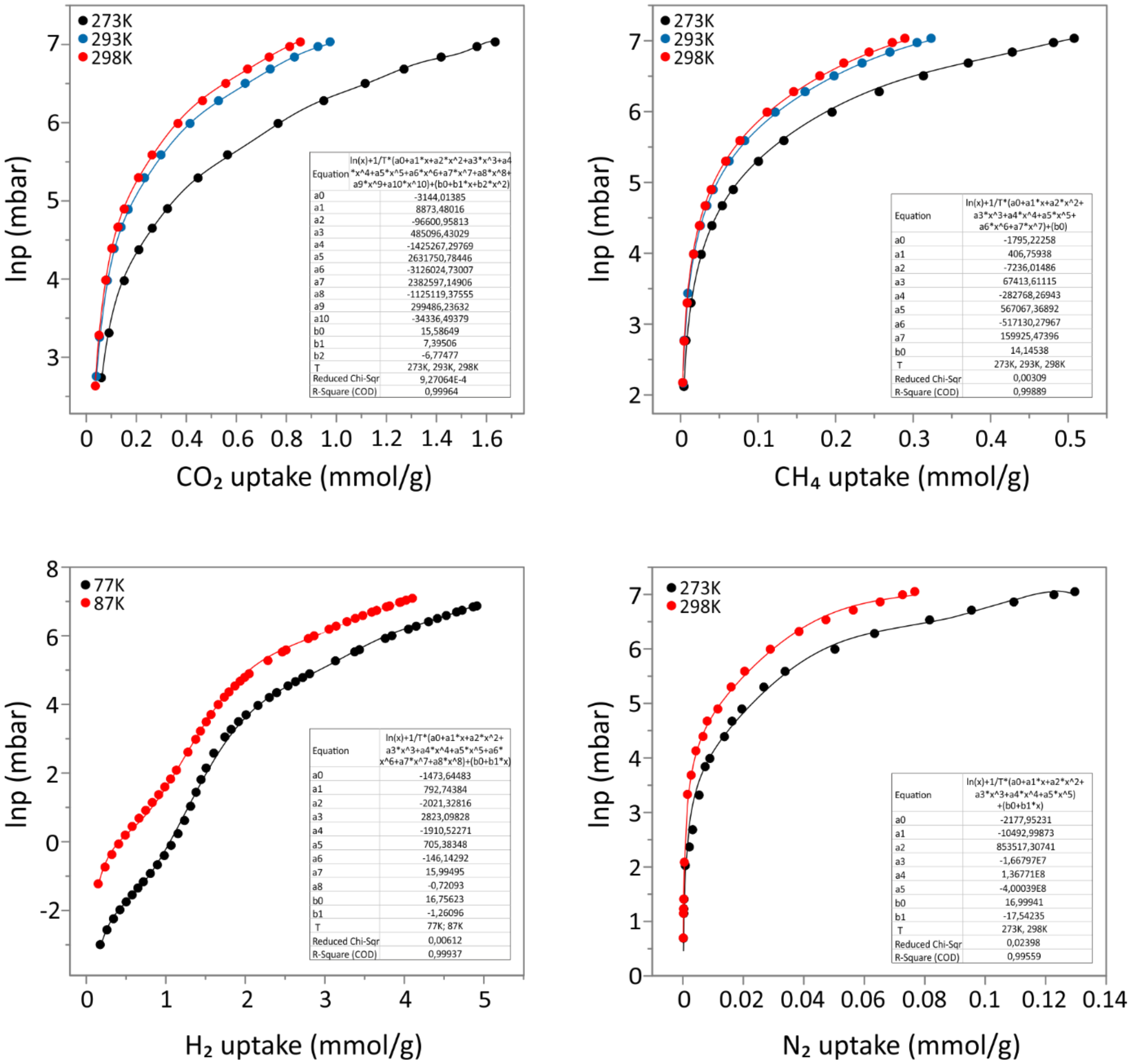

Figure S46. View of the virial fitting graph for CO_2, CH_4, H_2 and N_2 adsorption isotherms for **WUT-1’(Ni)**.

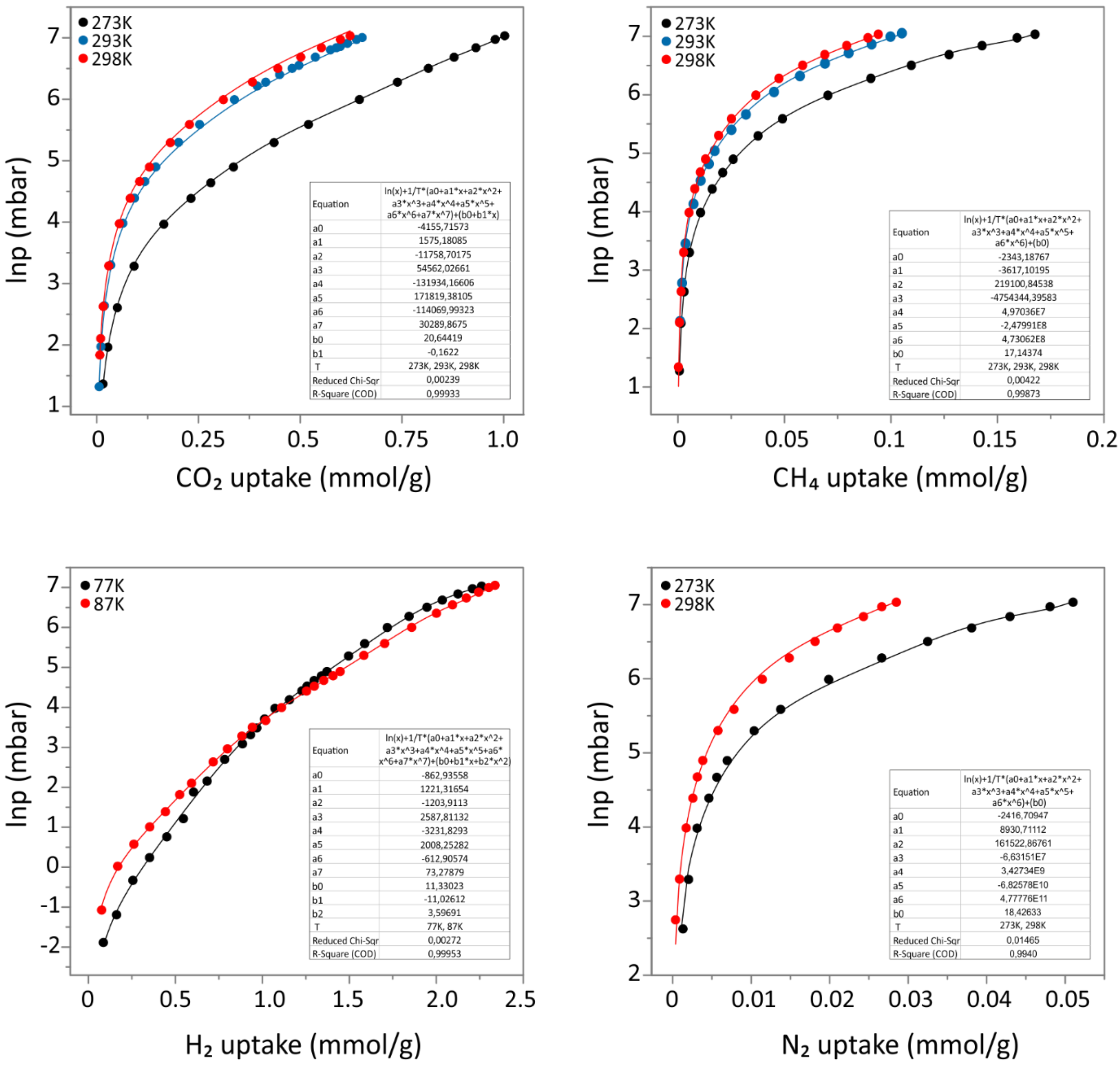

Figure S47. View of the virial fitting graph for CO_2, CH_4, H_2 and N_2 adsorption isotherms for **WUT-2'(Ni)**.

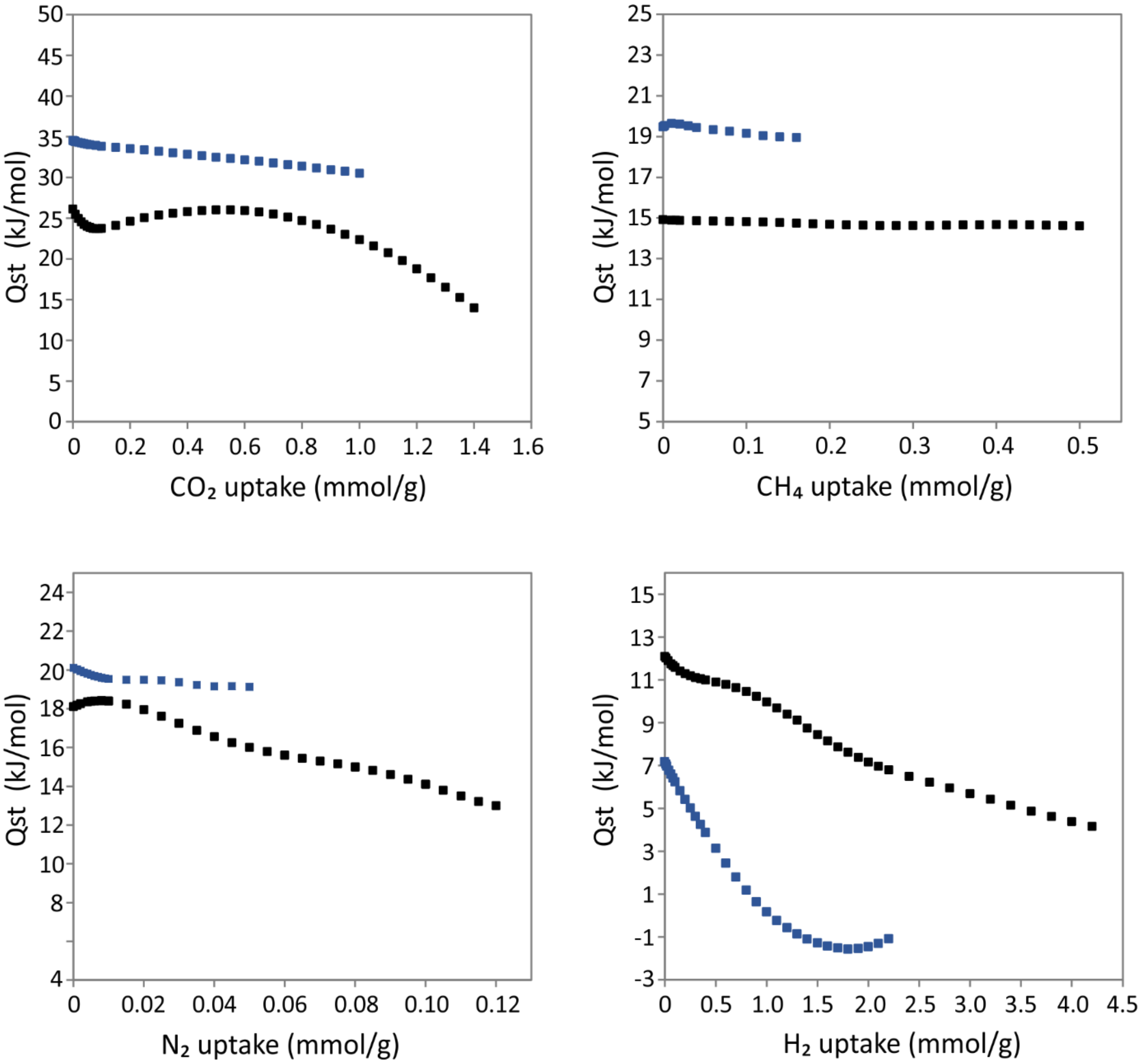

Figure S48. The isosteric heats of adsorption of CO_2, CH_4, H_2 and N_2 for **WUT-1'(Ni)** (black symbols) and **WUT-2'(Ni)** (blue symbols).

12. Ideal Adsorbed Solution Theory calculations

The adsorption selectivities of gas mixtures were calculated based on the ideal adsorbed solution theory (IAST) proposed by Myers and Prausnitz.[15]

$$S = \frac{q_1/q_2}{p_1/p_2} \quad (3)$$

q_i – molar loading amount [mmol/g]
p_i – mole fraction of each adsorptive

First, the pure component isotherm data for CO_2, CH_4 and N_2 were fitted with the single-site (4) or dual-site Langmuir-Freundlich isotherm model (5). The fitted isotherm parameters were then applied to predict the adsorption selectivity.

$$q = q_{A,sat} \frac{b_A p^{\nu}}{1+b_A p^{\nu}} \quad (4)$$

$$q = q_{A,sat} \frac{b_A p^{\nu_A}}{1+b_A p^{\nu_A}} + q_{B,sat} \frac{b_B p^{\nu_B}}{1+b_B p^{\nu_B}} \quad (5)$$

q – component molar loading, [mmol/g]
$q_{A,sat}$, $q_{B,sat}$ – saturation loading of site A and site B, [mmol/g]
b_A, b_B – dual-Langmuir-Freundlich constant at adsorption site A and B, [$\mathrm{bar}^{-\nu}$]
ν_A, ν_B – exponents in dual-Langmuir-Freundlich isotherm

Table S7. IAST selectivity for **WUT-1'(Ni)** and **WUT-2'(Ni)** at 273 and 298K under 1 bar.

mixture	mixture proportion	IAST selectivity **WUT-1'(Ni)** (298K)	**WUT-1'(Ni)** (273K)	**WUT-2'(Ni)** (298K)	**WUT-2'(Ni)** (273K)
CO_2/N_2	50:50	21.2	34.8	158.9	466.2
	20:80	4.5	6.0	15.9	41.2
	15:85	3.1	4.0	9.7	24.0
CO_2/CH_4	50:50	3.9	4.7	12.8	29.6

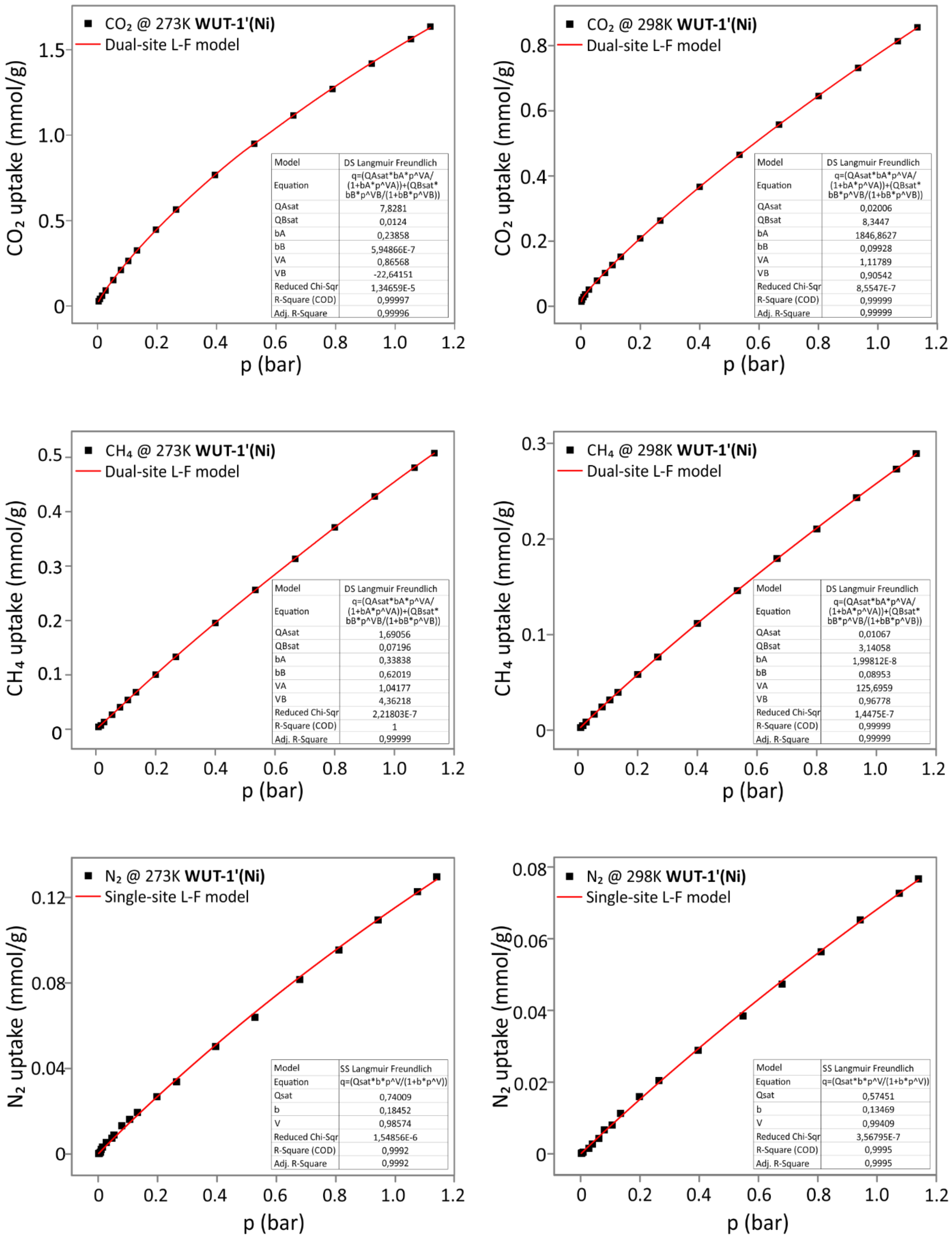

Figure S49. The single-site and dual-site Langmuir-Freundlich fitting of CO_2, CH_4, N_2 experimental adsorption data at 273K and 298K for **WUT-1’(Ni)**.

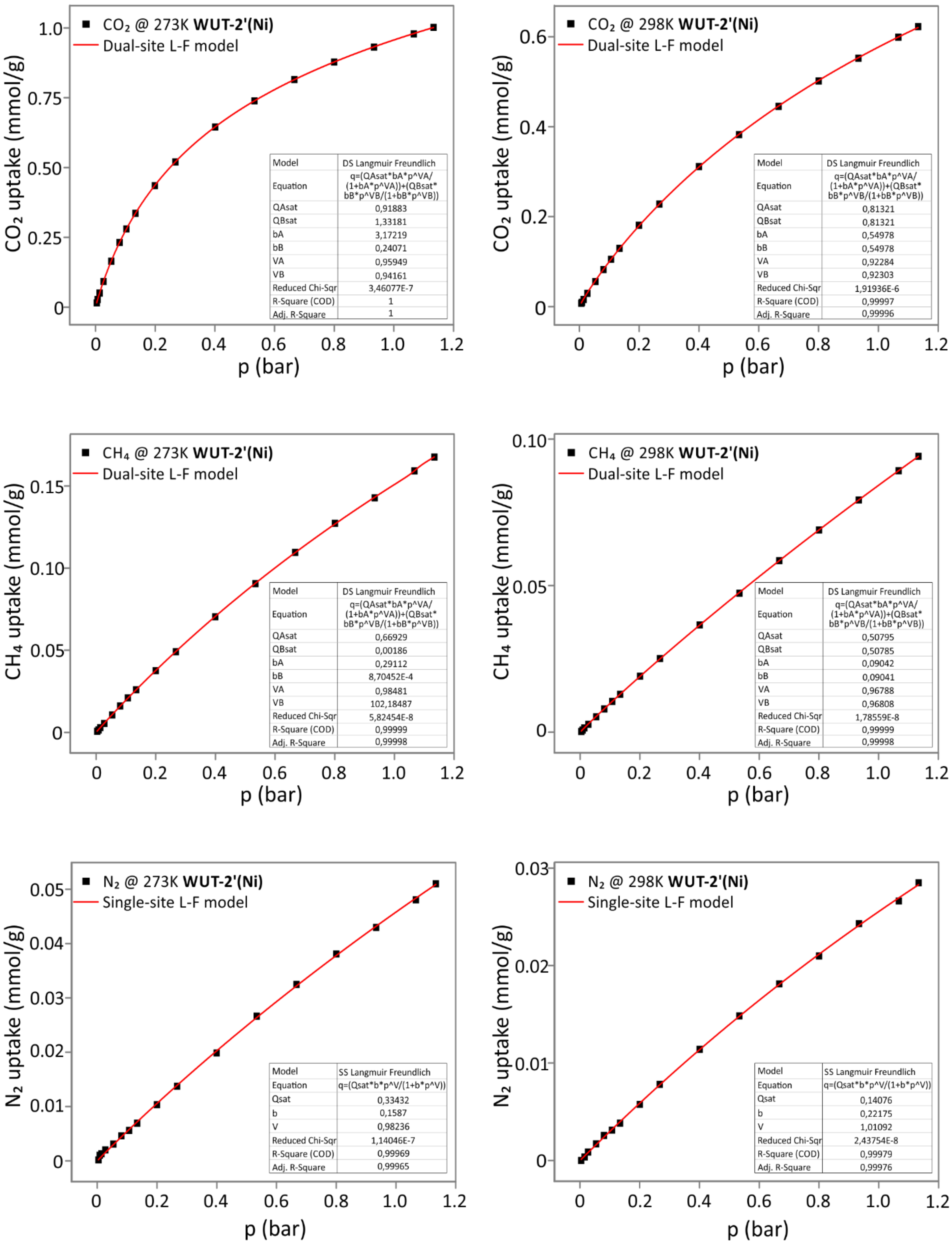

Figure S50. The single-site and dual-site Langmuir-Freundlich fitting of CO_2, CH_4, N_2 experimental adsorption data at 273K and 298K for **WUT-2'(Ni)**.

13. Breakthrough simulations

Breakthrough simulations were carried out for the gas mixture $CO_2/CH_4/He$ (5/5/90) and $CO_2/N_2/He$ (5/5/90) with He as the carrier gas, using the software of Ruptura.[16] The simulation type was set as Breakthrough at 273 K and operating at a total pressure of 100 kPa. The following parameter values were used: column length 0.3 m; column void fraction 0.4; column entrance velocity 0.1 m s^{-1}.

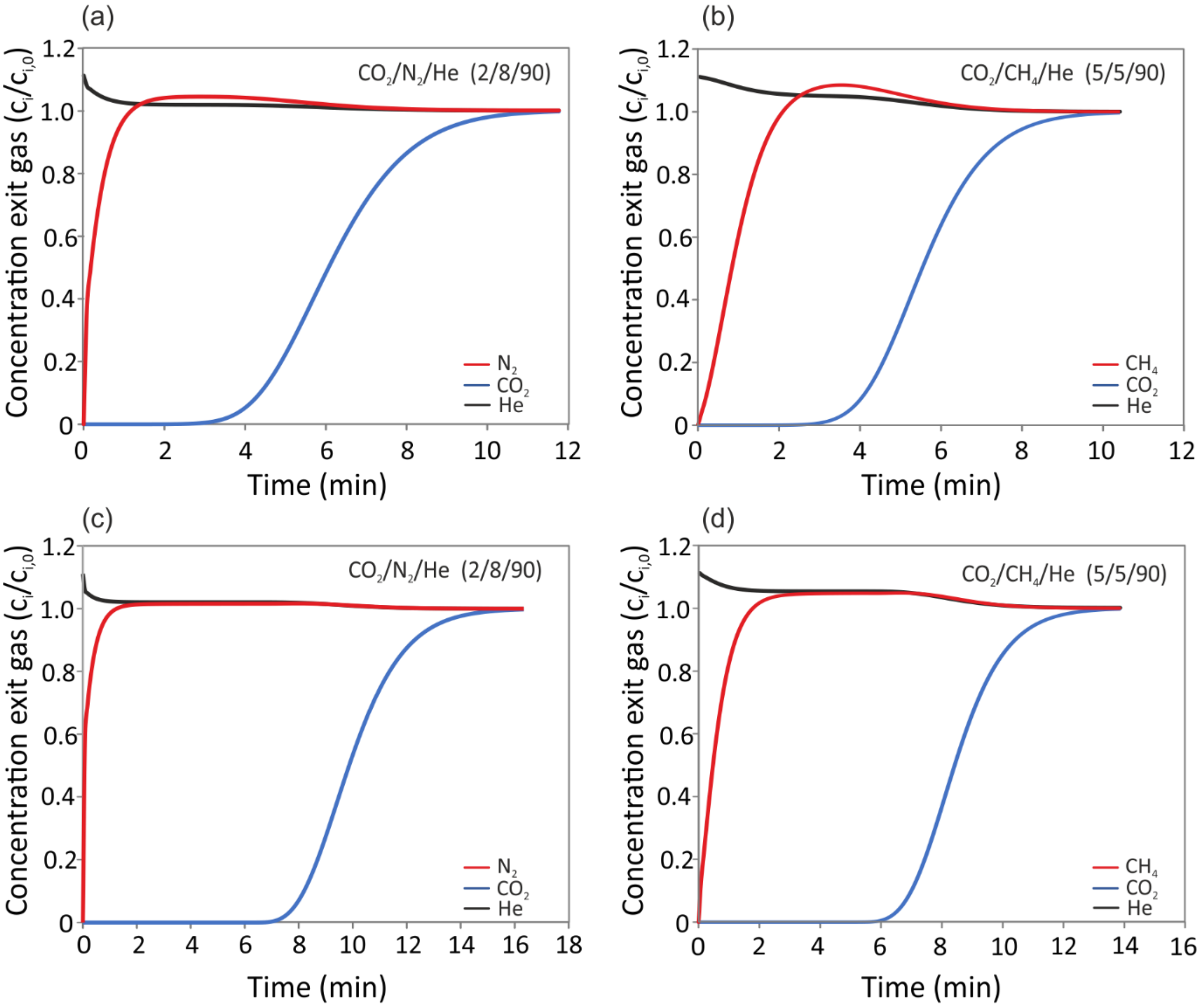

Figure S51. Simulated breakthrough curves of $CO_2/CH_4/He$ (5/5/90) and $CO_2/N_2/He$ (5/5/90) CO_2/N_2 on (a,b) **WUT-1’(Ni)** and (c,d) **WUT-2’(Ni).**

Table S8. IAST selectivity of CO_2 over N_2 and CH_4 at 1 bar for various non-covalent porous materials reported in the literature under different conditions.

sample	CO_2/N_2	CO_2/CH_4 (50:50)	Reference
CuTEI	-	3.5 (298K)	[17]
MPM-1-TIFSIX	74.1 (298K) (10:90)	20.3 (298K)	[18]
MPM-1-Cl	12.5 (298K) (10:90)	4 (298K)	[18]
SMOF-SIFSIX-1	131.7 (273K) (15:85)<br>~55 (298K) (15:85)	21.1 (273K)<br>11 (298K)	[19]
$[Fe_2L_3](BF_4)_4$	40(294,5K) (50:50)	20 (294,5K)	[20]
ZSA-10	-	25.9 (298K)	[21]
HOF-5	22.4 (296K) (10:90)<br>31.4 (273K) (10:90)	5 (296K)<br>6.1 (273K)	[22]
HOF-9	17.8 (296K) (10:90)<br>25.1 (273K) (10:90)	2.9 (296K)<br>4.6 (273K)	[23]
HOF-11	13.1 (298K) (15:85)<br>15.1 (273K) (15:85)	4,2 (298K)<br>4.0 (273K)	[24]
HOF-12	-	5.3 (298K)<br>3 (273K)	[25]
BTBA-1	2051 (298K) (20:80)	14 (298K)	[26]
PTBA-1	2340 (298K) (20:80)	6 (298K)	[26]
SOF-1	-	4.24 (298K)*<br>5.60 (273K)*	[27]
SOF-7	-	9.13 (298K)<br>14.2 (273K)	[28]
SOF-9	-	6.75 (298K)<br>7.19 (273K)	[29]
JLU-SOF1-2	30.0 (15:85)	-	[30]
JLU-SOF1-3	22.8 (15:85)	-	[30]

*-calc. form Henry law

14. Adsorption Information Files

WUT-1'(Ni)_ H2-10 bar_77K.aif

```
data_raw2aif
_exptl_operator 'PW'
_exptl_date 2018-03-30T08:24:52
_exptl_instrument 'ASAP 2050 V1.01 E'
_exptl_adsorptive 'H2'
_exptl_temperature -196.0
_adsnt_sample_mass 0.1
_adsnt_sample_id 'Sorpcja H2_WOT-Ni_10 bar_-196C_30032018'
_adsnt_material_id 'unknown'
_units_temperature '°C'
_units_pressure 'mmHg'
_units_mass 'g'
_units_loading 'cm³/g STP'
_audit_aif_version d546195

loop_
_adsorp_pressure
_adsorp_amount
2.470627546310425 30.52972062477977
5.379136562347412 33.84892815984476
11.527505874633789 37.84480706929776
21.75311279296875 42.75065837421669
29.515926361083984 45.851560408245334
40.065818786621094 49.517551719318135
50.18803787231445 52.62784061330336
96.46958923339844 63.30431861457016
150.3330078125 71.71627035619886
199.95626831054688 77.54739716130018
249.3429718017578 84.30824637035842
305.31353759765625 89.541596941476
350.6034240722656 93.37655520657941
401.50421142578125 97.31598088825109
451.73931884765625 100.93309135497458
502.2469787597656 104.31156015324845
553.2017822265625 107.56687411441358
603.1634521484375 110.5895234610151
651.1832275390625 113.39274200607132
701.6611328125 116.20738548937815
754.8541259765625 119.06840387750603
804.7396850585938 121.63809133539175
852.1994018554688 124.02926200430623
904.9981689453125 126.56598870221572
965.3820190429688 129.3770900639575
1006.7764892578125 131.29864312307262
1262.064208984375 141.81308964622352
1525.365234375 151.26609371016067
2050.3583984375 167.11096483839955
2562.298583984375 180.03983046357078
3075.56298828125 190.4866846346002
3583.319091796875 199.4942137690068
4108.12548828125 208.02990718098405
4601.3818359375 215.04295359803552
5121.55322265625 222.38566123616525
5638.3935546875 227.9879494220719
6121.26806640625 232.55532229696092
6603.56103515625 236.55579550437665
7044.31640625 240.71254135406963
7496.04052734375 245.60306952710945

loop_
_desorp_pressure
```

_desorp_amount
6774.8916015625 241.81746798623644
6380.07177734375 238.4322776433732
5849.052734375 233.51388243147755
5367.1552734375 228.4383380657772
4879.69921875 222.7405456234564
4379.24365234375 216.0423881195967
3913.31689453125 208.77400382175625
3406.85498046875 200.40223489147854
2927.00927734375 191.48801044232948
2423.31494140625 179.9965256512821
1953.966552734375 167.47813980964702
1448.7008056640625 151.27320119373286
1234.9249267578125 143.00397126621513
985.0938720703125 132.4594363896466
934.2908935546875 130.00535725256466
893.5021362304688 127.90812536471303
848.2106323242188 125.62305995310261
784.7897338867188 122.45094922201639
744.3565673828125 120.24056577148112
696.0838623046875 117.54402471187049
646.2906494140625 114.70739454237952
597.2987060546875 111.83646674521097
550.7069702148438 108.99670575979198
498.0780334472656 105.65319530284712
446.90386962890625 102.16460449788174
398.9427795410156 98.72831779394681
349.63677978515625 94.93267351900083
299.8075256347656 90.77349065791573
249.64846801757812 86.07732214657258
198.99432373046875 80.56876267278791
150.33692932128906 74.2929853806197
104.59977722167969 66.84461162476104

WUT-1'(Ni)_CH4-273K.aif

data_raw2aif
_exptl_operator 'KSB'
_exptl_date 2017-10-25T20:34:26
_exptl_instrument 'MicroActive 5.02'
_exptl_adsorptive 'CH4'
_exptl_temperature 273.15
_adsnt_sample_mass 0.1592
_adsnt_sample_id 'WUT-1'(Ni)_CH4-273K'
_adsnt_material_id 'unknown'
_units_temperature 'K'
_units_pressure 'mbar'
_units_mass 'g'
_units_loading 'cm³/g STP'
_audit_aif_version d546195

loop_
_adsorp_pressure
_adsorp_p0
_adsorp_amount
8.31555837896347 1013.25024 0.09642500878454253
15.93376463356018 1013.25024 0.15524405968255253
27.046223596801756 1013.2502399999998 0.2977520560258091
53.68769014389038 1013.2502400000001 0.5983086803640703
80.49816952349853 1013.25024 0.9072534107727533
106.73793052844238 1013.25024 1.2077644610807188
133.96152808850098 1013.25024 1.5237090677534515
199.80394757775878 1013.25024 2.252574752142219
267.32154300512695 1013.2502400000001 2.9860272773390006
399.837775392334 1013.25024 4.380326014645897
533.8314663706054 1013.25024 5.746246164928337

667.3257272768554 1013.25024 7.025969786032116
800.030258024414 1013.2502400000001 8.321705855550269
933.7415421489258 1013.25024 9.59545860377521
1066.987451025879 1013.2502400000001 10.787168528101041
1133.3230755117188 1013.2502400000001 11.383795008175658

loop_
_desorp_pressure
_desorp_p0
_desorp_amount
1048.7356112109376 1013.2502400000002 10.706018189357314
916.923748144043 1013.2502400000001 9.547821772522013
783.7485528691406 1013.25024 8.326557908536518
667.3483491196289 1013.25024 7.184054165806146
533.5850673061524 1013.2502400000002 5.867609599508351
400.6778350825195 1013.25024 4.486911099661355
267.3080960284424 1013.2502400000001 3.0412857964355995
200.47951066662597 1013.2502400000001 2.257036472068256
133.66071047241212 1013.2502400000001 1.5062060552450236
67.49541096734619 1013.2502399999998 0.7281430916621989

WUT-1'(Ni)_CO2-10 bar_273K.aif

data_raw2aif
_exptl_operator 'PW'
_exptl_date 2018-04-13T06:55:52
_exptl_instrument 'ASAP 2050 V1.01 E'
_exptl_adsorptive 'CO2'
_exptl_temperature 0.0
_adsnt_sample_mass 0.1
_adsnt_sample_id 'Sorpcja CO2_WOT-Ni_10 bar_0C_12042018'
_adsnt_material_id 'unknown'
_units_temperature '°C'
_units_pressure 'mmHg'
_units_mass 'g'
_units_loading 'cm³/g STP'
_audit_aif_version d546195

loop_
_adsorp_pressure
_adsorp_amount
2.992321014404297 0.4535988267034259
6.492245674133301 0.7354009361498811
10.235002517700195 1.0213943237212413
19.70391273498535 1.7012943798509221
30.134754180908203 2.423217013549309
40.7488899230957 3.146093996661272
50.61886215209961 3.8049838499548394
98.41851043701172 6.725213079687133
150.26171875 9.57671770300356
200.5962371826172 12.106877219686952
250.83834838867188 14.452192938931168
300.73187255859375 16.63280040069628
350.82037353515625 18.68289485982949
400.82354736328125 20.658944906972298
450.51727294921875 22.535916662014454
500.5455017089844 24.348173106440587
550.8228149414062 26.109310839091243
600.2275390625 27.801646386347112
650.8778076171875 29.497351482820324
700.0888671875 31.10831632915182
749.8124389648438 32.71633837389912
800.2667236328125 34.29826996767403
850.0789794921875 35.86796490323309
900.4464111328125 37.42783113095625
950.3297119140625 38.96713668135776

1000.7213134765625 40.51861641830186
1251.4578857421875 47.672252502181486
1498.2227783203125 54.911818984793506
1999.2109375 70.30491947824716
2500.0126953125 86.64315079254274
3002.816650390625 102.13756461689788
3502.302001953125 114.77630220961252
3999.975830078125 124.30292535250364
4496.4384765625 131.45977627465044
4996.7216796875 136.85492712835017
5495.44189453125 140.72536820545568
5995.81298828125 143.70046765704623
6512.2734375 146.89907699265777
6997.2841796875 148.4468106695762
7495.42041015625 153.21141494642748

loop_
_desorp_pressure
_desorp_amount
6992.2490234375 153.6974205195874
6493.21484375 153.19631634198964
5994.29736328125 151.92456571176118
5504.94091796875 149.85675612361968
5004.80517578125 146.68058912633805
4504.904296875 141.42335549365325
4003.075439453125 134.27248722139132
3504.666748046875 125.03478958030324
3005.226806640625 112.40180238928407
2502.49072265625 96.69368461219563
1999.1461181640625 79.79292715044673
1498.0421142578125 64.04161863745706
1250.505126953125 56.47187715879107
1000.3297119140625 48.936514683029486
953.1339111328125 47.25094979964293
898.8615112304688 45.327709717576646
849.7889404296875 43.62306542626129
799.9442138671875 41.86998201943015
749.1700439453125 40.09736416343957
700.2901611328125 38.357293832839666
649.9243774414062 36.54673469962335
600.3787841796875 34.742197334741746
549.8123779296875 32.84846832256669
500.21966552734375 30.94899771259931
450.5862731933594 28.972699636326436
400.59716796875 26.930571556560423
350.564208984375 24.762503215950808
300.31585693359375 22.495163347787813
250.71298217773438 20.03838805794244
200.65184020996094 17.333225347512105
151.16567993164062 14.093129605317415
100.97138214111328 10.024761059231926

WUT-1'(Ni)_CO2-273K.aif

data_raw2aif
_exptl_operator 'KSB'
_exptl_date 2017-10-23T11:58:37
_exptl_instrument 'MicroActive 5.02'
_exptl_adsorptive 'CO2'
_exptl_temperature 273.15
_adsnt_sample_mass 0.1592
_adsnt_sample_id 'WUT-1'(Ni)_CO2-273K'
_adsnt_material_id 'unknown'
_units_temperature 'K'
_units_pressure 'mbar'
_units_mass 'g'

_units_loading 'cm³/g STP'
_audit_aif_version d546195

loop_
_adsorp_pressure
_adsorp_p0
_adsorp_amount
3.602061199573517 1013.25024 0.6253670417472585
8.15573630580139 1013.2502399999997 0.9335972465368819
15.463943085182189 1013.25024 1.3572603709515185
27.40647949447632 1013.2502400000001 2.038098072343976
53.48653476498413 1013.2502399999998 3.4085852268038073
79.49510847070313 1013.2502400000001 4.719922353544082
104.62269668389892 1013.2502400000001 5.925051195291229
134.17683229083252 1013.25024 7.289164287897093
198.79515986242674 1013.25024 10.02019184835666
267.29401840686035 1013.2502399999998 12.66159434873328
399.5903591586914 1013.25024 17.198891183361415
533.6329963183593 1013.2502399999998 21.28648578135982
666.6760409721679 1013.2502399999998 24.995634807711852
800.0813606044921 1013.25024 28.474363691668177
933.5481986293945 1013.25024 31.80294298283093
1067.1438509604493 1013.2502400000001 35.0121251237262
1133.1561783911131 1013.2502399999998 36.654308446718666

loop_
_desorp_pressure
_desorp_p0
_desorp_amount
1052.0149646777343 1013.25024 34.86628958462953
921.8116122802734 1013.25024 31.91861963909174
789.0698124536133 1013.2502400000001 28.751716814399664
667.2391051486816 1013.25024 25.615561260061913
533.9049059860839 1013.25024 21.915692529804154
400.8859885854492 1013.2502399999998 17.823701918251555
267.4502962811279 1013.25024 13.199624184144346
200.21856608276366 1013.25024 10.576722854806313
133.8608283387451 1013.2502399999998 7.711115338022468
67.7875063576355 1013.2502400000001 4.500218683529714

WUT-1'(Ni)_H2-77K.aif

data_raw2aif
_exptl_operator 'KSB'
_exptl_date 2017-10-19T13:21:37
_exptl_instrument 'MicroActive 5.02'
_exptl_adsorptive 'H2'
_exptl_temperature 77.3
_adsnt_sample_mass 0.1592
_adsnt_sample_id 'WUT-1'(Ni)_H2-77K'
_adsnt_material_id 'unknown'
_units_temperature 'K'
_units_pressure 'mbar'
_units_mass 'g'
_units_loading 'cm³/g STP'
_audit_aif_version d546195

loop_
_adsorp_pressure
_adsorp_amount
0.024673710773383012 1.9797983399321133
0.050020599365234376 3.9265639973965665
0.07704824865129678 5.8309691942631945
0.10605293303119527 7.688869836540708
0.13792514598839375 9.49692013772691
0.1732552826046281 11.248951529939914

0.21300027276270458 12.938423602048603
0.26027496072363854 14.552861212760055
0.31115533304035664 16.080329988957313
0.39603391005063054 18.074840458793314
0.5097953836605549 20.0539427493808
0.6685527270269394 22.006652062600622
0.9007639500803947 23.919773490841273
1.261121087875843 25.85033002047008
1.852809120577812 27.698413071971512
2.8113952452278137 29.411962306369237
4.2301272552738185 30.952335621296886
6.122738683032989 32.36580489105611
8.54342652797699 33.777566889563055
13.178689410621642 35.961366517782665
21.169613792655944 39.06834207662361
26.364285400039673 40.8439516449723
33.0368059371643 42.94596016110015
40.2424273894043 45.055828235664634
52.99192098907471 48.40626500245751
66.81 51.63270297541829
76.85906874362182 53.71816227862592
93.72494942193603 56.88422801408029
106.3216336942749 58.99314378408428
119.52397101708983 61.047276010989506
132.9887481624756 62.97774115074305
193.9206613293457 70.23876100394341
252.34580171557616 75.68447373212247
268.6185354385986 77.01891946076876
373.18306021655275 84.30092269707785
403.79427873193356 86.18
488.67582698876953 90.82700138244947
532.5171210307617 93.025040711827
607.585020947754 96.51386621670603
666.6664388950195 98.98999977002472
726.7127028486328 101.50119281305653
802.65813496875 104.40071800485151
844.831271540039 105.99244779990714
934.100969053711 109.10599042747562
962.685132121582 110.1332571965832
1067.239139370117 113.43443218147053
1081.0636892578125 114.0008575004193
1135.605277678711 115.69973985557179
1199.6871807348632 117.75075586880175

loop_
_desorp_pressure
_desorp_amount
1065.4149888325196 114.15796568059012
931.2017113696289 110.1562265577987
801.0243171298828 105.72143257940313
666.9569424155274 100.48780680104126
533.25 94.52935343355567
400.5637493862305 87.45729886889487
151.4935172680664 66.74044119297281
133.71265730291748 64.40407983683191

WUT-1'(Ni)_N2-77K.aif

data_raw2aif
_exptl_operator 'KSB'
_exptl_date 2025-02-21T21:11:31
_exptl_instrument 'MicroActive 5.02'
_exptl_adsorptive 'N2'
_exptl_temperature 77.421
_adsnt_sample_mass 0.0566
_adsnt_sample_id 'WUT-1'(Ni)_N2-77K'

_adsnt_material_id 'unknown'
_units_temperature 'K'
_units_pressure 'mbar'
_units_mass 'g'
_units_loading 'cm³/g STP'
_audit_aif_version d546195

loop_
_adsorp_pressure
_adsorp_p0
_adsorp_amount
0.10020869067836907 1022.7850173799627 50.934849203143955
0.19375833384132385 1022.4202287793887 73.4921966954623
0.3884365303351879 1021.9803121688341 92.31329358522989
0.6028169761633873 1021.151483772137 110.92434862935968
0.7003911557192802 1020.9984692989007 133.66259272004712
0.8045484860558509 1020.9336454757262 156.40507658357163
0.9897753927869797 1020.8928445703513 179.42866476995957
1.155915675942421 1020.8618358822662 197.7690615589784
1.888972504983902 1020.8291951579661 217.31397552099003
1.9700244140625 1020.8080738083468 219.79916167510794
3.132419245130539 1020.7867622163764 237.65906709886744
4.02123852973938 1020.7557535282913 247.65539462925722
5.137089023838043 1020.7329050212813 255.99534503183366
6.054070862514496 1020.7116885504862 261.8579605077465
7.060437715175628 1020.7018963331961 266.6915674357193
7.9794055766944885 1020.6921041159063 271.00244132501086
9.393027265159606 1020.6823118986164 275.75216408034294
9.754266002563476 1020.6741517175413 277.24344858020214
21.51386071875 1020.6464071018863 296.66389050980837
30.37057662854004 1020.6382469208114 304.6728544915714
38.87517653709411 1020.6300867397364 309.87084706650205
50.77717748272705 1020.6186624862313 314.77452323345057
60.88647621917725 1020.6105023051564 317.65852247207806
72.56078129882812 1020.6039741602964 320.13237280243317
82.46829401147461 1020.5958139792215 321.7025674178392
92.81162303503417 1020.5892858343615 323.0963320587537
103.34296561157227 1020.5827576895016 324.2568365050809
152.70706114123536 1020.5745975084266 327.64844600621905
206.52385548449706 1020.5680693635666 329.62941244019686
258.0661984899902 1020.5615412187063 330.7008687697657
307.97594425964354 1020.5550130738463 331.54944092756716
358.33446507568357 1020.5484849289863 331.97517160919017
408.8258078444824 1020.5354286392667 332.3723484128096
459.7408626108398 1020.5289004944065 332.590858680541
510.7835517670898 1020.5223723495466 332.74839302747694
561.8559422636719 1020.5158442046865 332.7881453126467
612.9423290083008 1020.5011558787517 332.85836162154544
663.9977124360352 1020.4946277338915 332.88326032946236
714.9765233671875 1020.4848355166016 332.7391662110405
765.511685784668 1020.4848355166016 332.90012144740484
816.7226554116211 1020.4848355166016 332.87311860067393
867.5927512998047 1020.4848355166016 332.988638772923
918.5604954301757 1020.4848355166014 333.11294976447994
969.3626444165038 1020.4848355166015 333.55610127283387
1009.9042416079101 1020.4848355166016 335.26605762000946

loop_
_desorp_pressure
_desorp_p0
_desorp_amount
971.8284252788086 1020.4848355166016 333.9712475330718
919.0364492373046 1020.4848355166014 333.6939928998077
865.4916052485352 1020.4848355166016 333.6739326604505
811.6071894975586 1020.4848355166015 333.75366364993874
765.6135654506836 1020.4848355166016 333.563655110305

714.2806982680664 1020.4848355166015 333.60683834876824
663.5533315605469 1020.4848355166016 333.504106242858
612.4918451176758 1020.4848355166015 333.47313329116446
561.4527770837402 1020.4848355166016 333.40478761863307
510.55310191552735 1020.4848355166017 333.2833940929923
459.6548100974121 1020.4848355166016 332.9488603840912
408.3578693305664 1020.4848355166016 332.6290091155316
357.5881070112305 1020.4848355166016 332.1725634367588
306.3341314709473 1020.4848355166015 331.56832837097255
255.53529845617675 1020.4848355166015 330.80450450462195
204.5951602976074 1020.4848355166015 329.5563396617329
153.49248867425536 1020.4848355166014 327.6320228771688
103.10139810076905 1020.4848355166016 324.04081024314405

WUT-2'(Ni)_CH4-273K.aif

data_raw2aif
_exptl_operator 'KSB'
_exptl_date 2018-03-19T02:03:37
_exptl_instrument 'MicroActive 5.02'
_exptl_adsorptive 'CH4'
_exptl_temperature 273.15
_adsnt_sample_mass 0.1841
_adsnt_sample_id 'WUT-2'(Ni)_CH4-273K'
_adsnt_material_id 'unknown'
_units_temperature 'K'
_units_pressure 'mbar'
_units_mass 'g'
_units_loading 'cm³/g STP'
_audit_aif_version d546195

loop_
_adsorp_pressure
_adsorp_amount
3.587185735908508 0.015553101572243844
8.108715117095947 0.03379771948693815
13.883792364212036 0.06493662256794624
27.19 0.12193237319175308
53.6809463121643 0.23843494298642487
80.45355648284912 0.3602304171710334
106.43886244335937 0.47121612096672266
133.90164733831787 0.5818434273883208
199.65409821276856 0.8430004247390854
267.348253868042 1.1005987897250435
399.47965046411133 1.5782387117141152
533.7844324672851 2.030078125
667.0407571567382 2.4555233028823378
800.2907347104492 2.853494594961713
933.6846620478516 3.2009350623539787
1066.7204644570313 3.5682952552462632
1133.5552342075196 3.758326929089397

loop_
_desorp_pressure
_desorp_amount
1049.28374334375 3.5506409306998723
917.5453605791015 3.1992474343379715
784.4195590400391 2.8004968629989073
667.1408059182129 2.4365868766694936
533.8426145449218 2.008434502858018
400.4881126853027 1.5323377744287328
267.01582263354493 1.0246583991130818
200.81810594641112 0.7464320383780568
133.75960983270264 0.4656340473398648
67.16099116708374 0.17088026376964893
34.09362946746826 0.016524672361985557

WUT-2'(Ni)_CO2-273K.aif

data_raw2aif
_exptl_operator 'KSB'
_exptl_date 2018-03-19T08:47:19
_exptl_instrument 'MicroActive 5.02'
_exptl_adsorptive 'CO2'
_exptl_temperature 273.15
_adsnt_sample_mass 0.1841
_adsnt_sample_id 'WUT-2'(Ni)_CO2-273K'
_adsnt_material_id 'unknown'
_units_temperature 'K'
_units_pressure 'mbar'
_units_mass 'g'
_units_loading 'cm³/g STP'
_audit_aif_version d546195

loop_
_adsorp_pressure
_adsorp_amount
3.9209125896606443 0.34944785644516035
7.124563875263214 0.6170946233613323
13.547279738342285 1.1411562770278747
26.633751318786622 2.062832668856152
52.75752452105713 3.6966448625039408
80.25018876095581 5.194647690015726
103.47726245965576 6.284397386971167
133.71478318652345 7.537494283595903
198.95961577697753 9.766359970099598
267.3439410706787 11.66842763741844
401.08738808496093 14.473005842414617
532.8057936467285 16.570261646841153
666.1420678344726 18.273105642579107
799.8068876704101 19.684861665898183
933.1613488432617 20.88882708457339
1066.5086491450195 21.948241509973663
1132.94 22.471844122143633

loop_
_desorp_pressure
_desorp_amount
1050.1266917944336 22.016670128244293
919.3520971801757 21.07418087418817
786.6867884765625 19.931256698931623
667.0078822485351 18.686100299915008
533.7731215458984 17.016764989262526
400.9094648503418 14.89063328952045
267.75044256555174 12.043643888458417
199.56552311975096 10.134126458879939
133.66174798498534 7.831402198476781
67.87619842510986 4.788694215290939
33.259232866744995 2.653158491132728

WUT-2'(Ni)_H2-77K.aif

data_raw2aif
_exptl_operator 'KSB'
_exptl_date 2018-03-23T21:51:30
_exptl_instrument 'MicroActive 5.02'
_exptl_adsorptive 'H2'
_exptl_temperature 77.355
_adsnt_sample_mass 0.1841
_adsnt_sample_id 'WUT-2'(Ni)_H2-77K'
_adsnt_material_id 'unknown'
_units_temperature 'K'

_units_pressure 'mbar'
_units_mass 'g'
_units_loading 'cm³/g STP'
_audit_aif_version d546195

loop_
_adsorp_pressure
_adsorp_amount
0.15135866466536704 1.9358518772926732
0.3032284660005569 3.6299911375913703
0.7166208053340911 5.739252023926055
1.2706943982081413 7.89780655849503
2.132780330926895 10.104723978074984
3.359549628616333 12.243249564720662
6.5286127116622925 13.564532317496539
8.615171921024322 15.314080919598357
14.829802940093995 17.565595788359325
21.9073373621521 19.86398969988037
27.517376365356444 20.898060041887618
32.52669050280762 21.706324197658613
40.77539607252503 22.707941363793445
53.07536446353149 24.040978292442666
65.9221299527893 25.914157633038833
82.51999163552856 27.519972543655236
92.82773499499511 28.1882863187654
106.87810661444091 29.094795671608065
119.87686769567871 30.03404665555624
133.80044917565917 30.741791870233044
197.64415155102537 33.539642377561115
269.0290852668457 35.60790866732507
402.9350148874512 38.52747177456621
531.8837094331054 41.32341860528258
668.0558920078125 43.63533167154097
799.4247575493164 45.61570451533158
933.0255364130859 47.62849610280554
1063.8074546455077 49.49415448691485
1132.8011456572265 50.67537540399158

loop_
_desorp_pressure
_desorp_amount
1028.5352007231445 49.969927496941565
896.1756937646484 48.80410868106781
763.2127206694336 47.446004391210614
666.8872866694336 46.36382395000505
533.964308666748 44.65996928097831
400.56 42.6428673971883
267.80333536340333 40.01841908275736
133.96788539593504 36.07370514525247
67.77760930142212 32.307521766467715

WUT-2'(Ni)_N2-77K.aif

data_raw2aif
_exptl_operator 'KSB'
_exptl_date 2018-10-03T22:02:47
_exptl_instrument 'MicroActive 5.02'
_exptl_adsorptive 'N2'
_exptl_temperature 77.26
_adsnt_sample_mass 0.1841
_adsnt_sample_id 'WUT-2'(Ni)_N2-77K'
_adsnt_material_id 'unknown'
_units_temperature 'K'
_units_pressure 'mbar'
_units_mass 'g'
_units_loading 'cm³/g STP'

_audit_aif_version d546195

loop_
_adsorp_pressure
_adsorp_p0
_adsorp_amount
0.0010868307228476168 1003.6240305401253 2.0402594787163792
0.03247127807403802 1003.3351011141418 4.021319878252499
0.15456845389831067 1003.0079349058415 5.864467613804278
0.3780202807703018 1002.7302576071351 7.324646915101654
1.1296500629467965 1002.5914189577818 9.182767718754
2.0242936742277147 1002.4690526905551 10.363778603725393
2.8755449273529052 1002.4008099646018 11.12418029842139
3.828403912668228 1002.3443332258818 11.783023469010722
4.99916851051712 1002.3019756718419 12.411021370144343
5.955392736522675 1002.2690309075887 12.845303644905757
8.721893312381745 1002.2598357725533 13.673083742919578
9.94834315537262 1002.257142528468 14.086824322432859
10.36025853325653 1002.254449284383 14.203161607054223
20.625498206680298 1002.2436763080423 16.062006835326198
29.645413827072144 1002.2382898198717 17.112023390551197
39.71506076559448 1002.2338010797296 17.972655885801803
49.81791573522949 1002.2311078356445 18.798706419198354
60.60350483651733 1002.2275168435308 19.52900234842565
69.75191435476684 1002.2248235994458 20.1
80.36541368673706 1002.2221303553606 20.74352253503995
90.49273157537841 1002.2194371112753 21.290525811128425
100.4807939161377 1002.2167438671901 21.79952498403363
148.15315399328614 1002.214050623105 23.88103444500682
200.91384191052245 1002.2104596309912 25.882284908328792
251.46218684069822 1002.2077663869062 27.61202363359557
302.34056248608397 1002.2059708908491 29.18820834391554
353.0743983896484 1002.2032776467643 30.698583717192072
401.0191970625 1002.2005844026788 32.11708602555731
451.2254476508789 1002.1987889066222 33.57453490834977
501.02784138427734 1002.196095662537 35.17800642695345
551.3976120915527 1002.1934024184516 36.939467423052
601.3607185568848 1002.1924225304492 38.93354480600437
651.3206107675782 1002.1880159302814 41.36338925691564
701.068077364746 1002.1844249381679 44.59180908409688
749.9883006035157 1002.1808339460542 49.66828298476261
798.7773497036133 1002.1745497098552 58.71946162903248
832.6311815346679 1002.164674481543 68.4888156669984
854.6589161279296 1002.1583902453441 76.1915167737525
881.1427468798828 1002.1476172690034 86.81923838293416
903.2109241201172 1002.1413330328046 96.0446651171719
922.9569447875976 1002.1323555525205 106.54283853012906
935.5328177783202 1002.124275820265 116.82502092190704
944.0280521015625 1002.1152983399811 128.13226722891847
948.309764774414 1002.1090141037822 137.01853406679174
952.9653074677734 1002.1000366234981 148.7028533068816
956.0526193916015 1002.0515176818476 160.6335832866016
959.6944919575195 1002.0001218916944 172.7453363454383
965.401055230957 1001.9487261015413 184.56667556881095
990.3155973574219 1001.9253643787445 190.02072228537045

loop_
_desorp_pressure
_desorp_p0
_desorp_amount
924.2092021201172 1001.8786409331506 168.4823391424481
919.0174078300781 1001.822572798438 149.6434597288767
913.936525668457 1001.7665046637254 133.4292452626604
908.1891128803711 1001.7057641844536 122.74515222617791
898.9628187172851 1001.649696049741 111.78098416332772
882.0931083969726 1001.5936279150285 101.17875673923669

824.3055296586914 1001.5609215031128 84.01633961371559
794.0389687514648 1001.5328874357566 78.09085152830237
710.3927521318359 1001.5001810238409 64.84417203928344
688.349312446289 1001.4814916456032 61.716338704682336
603.2 1001.4533148884882 49.93739331671414
511.7772040048828 1001.317959586025 40.812385152022735
484.7591559946289 1001.3039425523469 39.25133394595897
399.1772257207031 1001.28058082955 33.74522762620532
294.75044599438473 1001.2665637958718 30.741365624746646
187.7677235288086 1001.2525467621939 27.256010894672407
99.67582656298828 1001.2385297285157 23.563906156659005

References

1 Technologies, A. CrysAlisPro,Data Collection and Processing Software for Agilent X-Ray Diffractometers, Version 1.171.35.21b. 2012.

2 Sheldrick, G. M. A Short History of SHELX. *Acta Crystallogr. Sect. A Found. Crystallogr.* **2008**, *64*, 112–122, DOI: 10.1107/S0108767307043930.

3 Dolomanov, O. V.; Bourhis, L. J.; Gildea, R. J.; Howard, J. A. K.; Puschmann, H. OLEX2: A Complete Structure Solution, Refinement and Analysis Program. *J. Appl. Crystallogr.* **2009**, *42*, 339–341, DOI: 10.1107/S0021889808042726.

4 Farrugia, L. J. WinGX and ORTEP for Windows : An Update. *J. Appl. Crystallogr.* **2012**, *45*, 849–854, DOI: 10.1107/S0021889812029111.

5 Dubbeldam, D.; Calero, S.; Ellis, D. E.; Snurr, R. Q. RASPA: Molecular Simulation Software for Adsorption and Diffusion in Flexible Nanoporous Materials. *Mol. Simul.* **2016**, *42*, 81–101, DOI: 10.1080/08927022.2015.1010082.

6 Mayo, S. L.; Olafson, B. D.; Goddard III, W. A. DREIDING: A Generic Force Field for Molecular Simulations. *J. Phys. Chem.* **1990**, *94*, 8897–8909, DOI: 10.1021/j100389a010.

7 Casewit, C. J.; Colwell, K. S.; Rappé, A. K. Application of a Universal Force Field to Organic Molecules. *J. Am. Chem. Soc.* **1992**, *114*, 10035–10046, DOI: 10.1038/220833b0.

8 Potoff, J. J.; Siepmann, J. I. Vapor-Liquid Equilibria of Mixtures Containing Nitrogen, Oxygen, Carbon Dioxide, and Ethane. *AIChE J.* **2003**, *49*, 2187–2198, DOI: 10.1002/aic.690490826.

9 Feynman, R. A.; Hibbs, A. R. *Quantum Mechanics and Path Integrals*; McGraw-Hill: New York, 1965.

10 Rappe, A. K.; Goddard III, W. A. Charge Equilibration for Molecular Dynamics Simulations. *J. Phys. Chem.* **1991**, *95*, 3358–3363, DOI: https://doi.org/10.1021/j100161a070.

11 Reid, R. C.; Prausnitz, J. M.; Poling, B. E. *The Properties of Gases and Liquids*; McGraw Hill Book Co.: New York, NY, United States, 1987.

12 Osterrieth, J. W. M.; Rampersad, J.; Madden, D.; Rampal, N.; Skoric, L.; Connolly, B.; Allendorf, M. D.; Stavila, V.; Snider, J. L.; Ameloot, R.; et al. How Reproducible Are Surface Areas Calculated from the BET Equation? *Adv. Mater.* **2022**, *34*, DOI: 10.1002/adma.202201502.

13 Lemmon, E. W.; McLinden, M. O.; Friend, D. G. Thermophysical Properties of Fluid Systems. NIST Chemistry WebBook, NIST Standard Reference Database Number 69. National Institute of Standards and Technology: Gaithersburg MD. 2005, p 20899.

14 Madden, D. G.; O'Nolan, D.; Rampal, N.; Babu, R.; Çamur, C.; Al Shakhs, A. N.; Zhang, S. Y.; Rance, G. A.; Perez, J.; Maria Casati, N. Pietro; et al. Densified HKUST-1 Monoliths as a Route to High Volumetric and Gravimetric Hydrogen Storage Capacity. *J. Am. Chem. Soc.* **2022**, *144*, 13729–13739, DOI: 10.1021/jacs.2c04608.

15 Myers, A. L.; Prausnitz, J. M. Thermodynamics of Mixed-Gas Adsorption. *AIChE J.* **1965**, *11*, 121–127, DOI: 10.1002/aic.690110125.

16 Sharma, S.; Balestra, S. R. G.; Baur, R.; Agarwal, U.; Zuidema, E.; Rigutto, M. S.; Calero, S.; Vlugt, T. J. H.; Dubbeldam, D. RUPTURA: Simulation Code for Breakthrough, Ideal Adsorption Solution Theory Computations, and Fitting of Isotherm Models. *Mol. Simul.* **2023**, *49*, 893–953, DOI: 10.1080/08927022.2023.2202757.

17 Zhao, D.; Yuan, D.; Krishna, R.; van Baten, J. M.; Zhou, H.-C. Thermosensitive Gating Effect and Selective Gas Adsorption in a Porous Coordination Nanocage. *Chem. Commun.* **2010**, *46*, 7352–7354, DOI: 10.1039/c0cc02771e.

18 Nugent, P. S.; Rhodus, V. Lou; Pham, T.; Forrest, K.; Wojtas, L.; Space, B.; Zaworotko, M. J. A Robust Molecular Porous Material with High CO_2 Uptake and Selectivity. *J. Am. Chem. Soc.* **2013**, *135*, 10950–10953, DOI: 10.1021/ja4054948.

19 Dai, J.; Xie, D.; Liu, Y.; Zhang, Z.; Yang, Y.; Yang, Q.; Ren, Q.; Bao, Z. Supramolecular Metal−Organic Framework for CO_2/CH_4 and CO_2/N_2 Separation. *Ind. Eng. Chem. Res.* **2020**, *59*, 7866–7874, DOI: 10.1021/acs.iecr.0c00447.

20 Wilson, B. H.; Scott, H. S.; Qazvini, O. T.; Telfer, S. G.; Mathonière, C.; Clérac, R.; Kruger, P. E. A Supramolecular Porous Material Comprising Fe(Ii) Mesocates. *Chem. Commun.* **2018**, *54*, 13391–13394, DOI: 10.1039/c8cc07227b.

21 Li, J.; Kan, L.; Li, J.; Liu, Y.; Eddaoudi, M. Quest for Zeolite-like Supramolecular Assemblies: Self-Assembly of Metal–Organic Squares via Directed Hydrogen Bonding. *Angew. Chem. Int. Ed.* **2020**, *59*, 19659–19662, DOI: 10.1002/anie.202006978.

22 Wang, H.; Li, B.; Wu, H.; Hu, T.-L.; Yao, Z.; Zhou, W.; Xiang, S.; Chen, B. A Flexible Microporous

Hydrogen-Bonded Organic Framework for Gas Sorption and Separation. *J. Am. Chem. Soc.* **2015**, *137*, 9963–9970, DOI: 10.1021/jacs.5b05644.
23 Wang, H.; Wu, H.; Kan, J.; Chang, G.; Yao, Z.; Li, B.; Zhou, W.; Xiang, S.; Zhaoa, J. C.-G.; Chen, B. A Microporous Hydrogen-Bonded Organic Framework with Amine Sites for Selective Recognition of Small Molecules. *J. Mater. Chem. A* **2017**, *5*, 8292–8296, DOI: 10.1039/C7TA01364G.
24 Yang, W.; Wang, J.; Wang, H.; Bao, Z.; Zhao, J. C.-G.; Chen, B. Highly Interpenetrated Robust Microporous Hydrogen-Bonded Organic Framework for Gas Separation. *Cryst. Growth Des.* **2017**, *17*, 6132–6137, DOI: 10.1021/acs.cgd.7b01322.
25 Yang, W.; Zhou, W.; Chen, B. A Flexible Microporous Hydrogen-Bonded Organic Framework. *Cryst. Growth Des.* **2019**, *19*, 5184–5188, DOI: 10.1021/acs.cgd.9b00582.
26 Ding, X.; Liu, Z.; Zhang, Y.; Ye, G.; Jia, J.; Chen, J. Binary Solvent Regulated Architecture of Ultra-Microporous Hydrogen-Bonded Organic Frameworks with Tunable Polarization for Highly-Selective Gas Separation. *Angew. Chem. Int. Ed.* **2022**, *61*, e202116483, DOI: 10.1002/anie.202116483.
27 Yang, W.; Greenaway, A.; Lin, X.; Matsuda, R.; Blake, A. J.; Wilson, C.; Lewis, W.; Hubberstey, P.; Kitagawa, S.; Champness, N. R.; et al. Exceptional Thermal Stability in a Supramolecular Organic Framework: Porosity and Gas Storage. *J. Am. Chem. Soc.* **2010**, *132*, 14457–14469, DOI: 10.1021/ja1042935.
28 Lu, J.; Perez-krap, C.; Suyetin, M.; Alsmail, N. H.; Yan, Y.; Yang, S.; Lewis, W.; Bichoutskaia, E.; Tang, C. C.; Blake, A. J.; et al. A Robust Binary Supramolecular Organic Framework (SOF) with High CO_2 Adsorption and Selectivity. *J. Am. Chem. Soc.* **2014**, 5–8, DOI: 10.1021/ja506577g.
29 Lu, J.; Perez-Krap, C.; Trousselet, F.; Yan, Y.; Alsmail, N. H.; Karadeniz, B.; Jacques, N. M.; Lewis, W.; Blake, A. J.; Coudert, F.-X.; et al. Polycatenated 2D Hydrogen-Bonded Binary Supramolecular Organic Frameworks (SOFs) with Enhanced Gas Adsorption and Selectivity. *Cryst. Growth Des.* **2018**, *18*, 2555–2562, DOI: 10.1021/acs.cgd.8b00153.
30 Zhou, Y.; Kan, L.; Eubank, J. F.; Li, G.; Zhang, L.; Liu, Y. Self-Assembly of Two Robust 3D Supramolecular Organic Frameworks From a Geometrically Non- Planar Molecule for High Gas Selectivity Performance. *Chem. Sci.* **2019**, *10*, 6565–6571, DOI: 10.1039/c9sc00290a.
